# Physiologically Based Pharmacokinetic Modeling and Simulations in Lieu of Clinical Pharmacology Studies to Support the New Drug Application of Asciminib

**DOI:** 10.3390/pharmaceutics17101266

**Published:** 2025-09-26

**Authors:** Ioannis Loisios-Konstantinidis, Felix Huth, Matthias Hoch, Heidi J. Einolf

**Affiliations:** 1 Pharmacokinetic Sciences, Translational Medicine, Biomedical Research, Novartis, 4056 Basel, Switzerland; felix.huth@novartis.com (F.H.); matthias.hoch@novartis.com (M.H.); 2 Pharmacokinetic Sciences, Translational Medicine, Biomedical Research, Novartis, East Hanover, NJ 07936, USA; heidi.einolf@novartis.com

**Keywords:** asciminib, PBPK, modeling and simulation, drug–drug interactions, clinical pharmacology, chronic myeloid leukemia

## Abstract

**Background**: Asciminib (Scemblix^®^) is approved for the first-line treatment of adult patients with chronic myeloid leukemia in the chronic phase at 40 mg twice daily (BID) and 80 mg once daily (QD) or 200 mg BID for patients harboring the T315I mutation. **Objectives**: (1) Extrapolate the DDI magnitude as the perpetrator or victim of other drugs and the effect of organ impairment to untested doses; (2) Predict clinically untested DDI scenarios. **Methods**: Asciminib is primarily cleared by cytochrome P450 (CYP)3A4, UDP-glucuronosyltransferases (UGT)2B7, UGT2B17, UGT1A3/4, and the breast-cancer-resistance protein (BCRP). In vitro asciminib is an inhibitor of several CYP, UGT enzymes, and transporters and is an inducer of CYP1A2 and CYP3A4. Clinical DDI studies assessed asciminib 40 mg BID as a perpetrator on CYP-sensitive substrates. Additional studies evaluated the impact of strong CYP3A4 perpetrators and imatinib on a single 40 mg dose of asciminib. Hepatic and renal impairment studies were also conducted at the 40 mg dose. A nonlinear whole-body physiologically based pharmacokinetic (PBPK) model was developed and verified for asciminib as a CYP3A4, UGT, and BCRP substrate and a perpetrator of several CYP and UGT enzymes. **Results**: This PBPK model was applied in lieu of clinical pharmacology studies to support the new drug application of Scemblix^®^ and to bridge data from 40 mg BID to the 80 mg QD and 200 mg BID dose regimens. **Conclusions**: The PBPK predictions informed the drug product label and are estimated to have replaced at least 10 clinical studies.

## 1. Introduction

Asciminib (Scemblix) is a novel first-in-class BCR::ABL1 selective allosteric inhibitor that specifically targets the myristoyl pocket of the BCR::ABL1 tyrosine kinase. By binding to this distinct site, asciminib inhibits the kinase activity of BCR::ABL1, including its mutant form T315I (substitution of the threonine-315 residue with isoleucine).

The ASCEMBL (NCT03106779) trial, a randomized Phase III study, demonstrated that asciminib 40 mg twice daily (BID) administered orally had superior efficacy and a better safety and tolerability profile than bosutinib in patients with chronic myeloid leukemia (CML) in the chronic phase (CML-CP), newly diagnosed, or with prior treatment with ATP-competitive tyrosine kinase inhibitors (TKIs) [1]. Population pharmacokinetic (PopPK) and exposure–response analyses supported the comparability of 80 mg once daily (QD) and 40 mg BID regimens in terms of efficacy and safety, enabling a more patient-centric QD dosing option, considering that asciminib is administered under fasting conditions [2]. Additionally, based on the available clinical data and the results of Combes et al., 2024, the 200 mg BID regimen was shown to have a positive risk–benefit profile in patients with CML harboring the T315I mutation [2].

The pharmacokinetics (PK) of asciminib was characterized in a Phase I dose-escalation study (NCT02081378), with exposures increasing slightly more than proportionally across doses from 10 to 200 mg BID and 80 to 200 mg QD. Furthermore, the clinical pharmacology package included a human absorption, distribution, metabolism, and excretion (hADME) mass balance study, one drug–drug interaction (DDI) to assess the perpetrator potential of asciminib 40 mg BID on cytochrome P450 (CYP)-sensitive substrates (midazolam, warfarin, and repaglinide), as well as two victim DDI studies, which evaluated the effects of strong CYP3A4 inhibitors, clarithromycin and itraconazole, and inducer, rifampicin;strong P-glycoprotein (P-gp) inhibitor, quinidine, and imatinib, a CYP3A4, uridine 5′-diphospho-glucuronosyltransferase (UGT)1A3/4, UGT2B17, and breast-cancer-resistance protein (BCRP) inhibitor on a single 40 mg dose of asciminib [3,4,5,6]. Dedicated food and organ impairment (hepatic and renal) studies with the 40 mg dose complimented the asciminib clinical pharmacology package [6,7].

Based on in vitro phenotyping, hADME. and clinical DDI studies, asciminib is primarily cleared by CYP3A4 (relative contribution to a total clearance of 35.1%), UGT2B7 (13.1%), UGT2B17 (7.6%), UGT1A3/4 (6.6%) and is biliary excreted by BCRP (31.1%). Renal excretion was a minor (~4.4%) pathway [3,8].

In vitro, asciminib is a reversible inhibitor of CYP1A2, CYP2B6, CYP2C8, CYP2C9, and CYP3A4/5. According to the static Net Effect Model [9], AUC increases of sensitive-probe substrates of CYP3A4/5, CYP2C9, and CYP2C8 by oral administration of asciminib at a therapeutic dose of 40 mg BID were flagged with AUC ratio (AUCR) values of 1.93-fold, 1.26-fold, and 1.17-fold, respectively. Based on these estimates, a dedicated clinical DDI study in healthy volunteers (HVs) was performed. Asciminib showed no in vitro apparent time-dependent inhibition (TDI) of CYP1A2, CYP2C9, CYP2D6, or CYP3A4/5 at asciminib concentrations of up to 50 μM (in-house data). In addition, asciminib was found to be an in vitro inducer of CYP3A4/5 and CYP1A2 mRNA (in-house data). No induction potential for CYP2C9, CYP2C19, CYP2B6, and UGT1A1 was anticipated based on in vitro data [8].

The purpose of this modeling and simulation study was to (1) develop and verify a physiologically-based pharmacokinetic (PBPK) model for asciminib, including the evaluation of its predictive performance against observed PK, DDI, and organ impairment (OI) data and to (2) apply the established PBPK model to predict various DDI or OI scenarios at different asciminib doses (40 mg BID, 80 mg QD, and 200 mg BID) and/or regimens (single vs. multiple dosing), which had not been tested clinically. The PBPK model supported the new drug application (NDA) of asciminib (NDA 215358) by predicting the impacts of untested DDI and organ impairment scenarios and bridging clinical pharmacology data from the 40 mg BID dose to the 80 mg QD and 200 mg BID regimens. The regulatory review as well as the impacts of the PBPK simulations on the drug product label are also discussed.

## 2. Materials and Methods

### 2.1. PBPK Platform

Simcyp Population-based Simulator, Version 19, Release 1 (Certara Inc., Princeton, NJ, USA) was used for all the simulations. The population files used were the Simcyp North European Caucasian (“Sim-NEurCaucasian”) population for healthy subject simulations and the Simcyp Cancer (“Sim-Cancer”) population for the cancer patient simulations. In addition, the Simcyp population files of Child–Pugh (CP) classes A, B, and C for simulations in healthy subjects with mildly, moderately, and severely impaired hepatic function were used. The HV control cohorts of the HI and RI studies were simulated using “Sim-NEurCaucasian” populations with matched demographics to the Simcyp hepatic and renal impairment populations, respectively. Simulations with a modified CP-C population accounting for the reduced abundance of UGT1A4 by 85% compared to the “Sim-NEurCaucasian” population (from 52 to 7.8 pmol UGT enzyme/mg liver microsomal protein) and UGT2B7 by 85% (from 71 to 10.65 pmol UGT enzyme/mg liver microsomal protein) in CP-C subjects, according to Prasad et al., were also incorporated [10]. Simulations in healthy subjects with moderately (glomerular filtration rate (GFR) = 30–60 mL/min) and severely (GFR < 30 mL/min) impaired renal function were performed using the “Sim-RenalGFR_less_30” and “Sim-RenalGFR_30-60” population, respectively. To predict the PK in healthy subjects with mild renal impairment, a customized population model file named “Mild RI_RenalGFR_60-90” was used according to the publication on PBPK modeling for HI and RI by the International Consortium for Innovation and Quality (IQ) [11].

### 2.2. Asciminib (As a Victim and a Perpetrator Compound)

**Figure 1:**
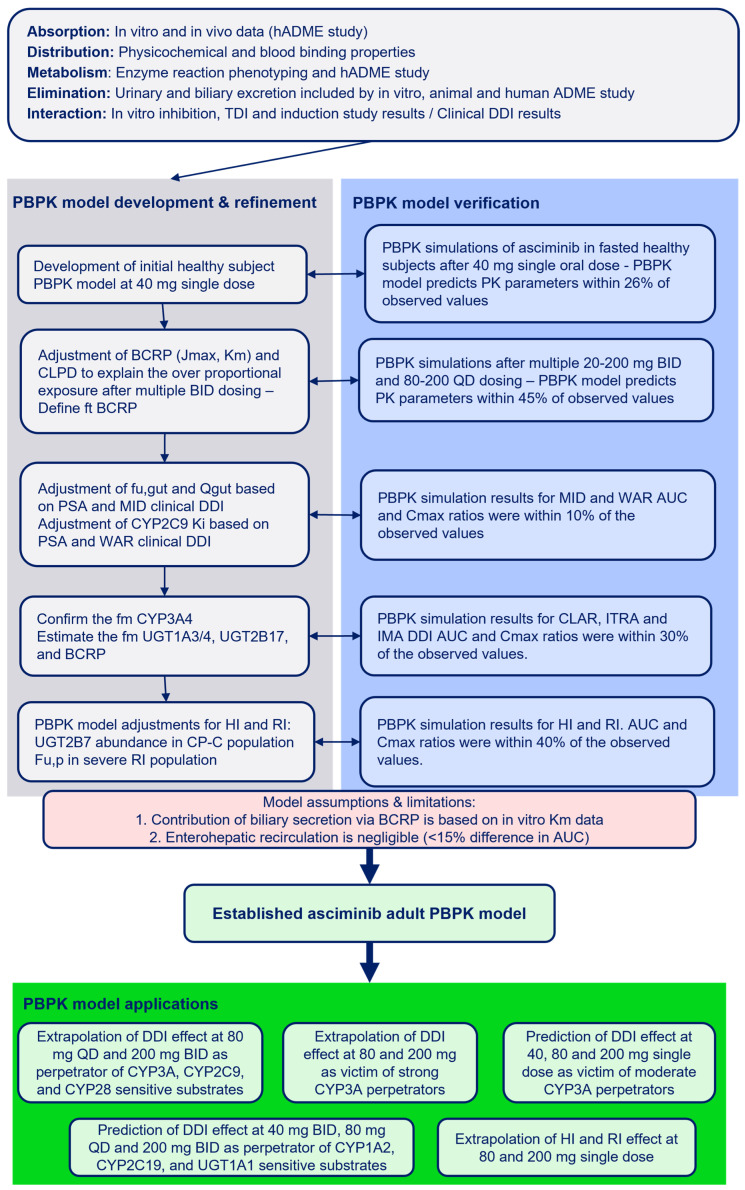
Development, verification, and applications of the asciminib PBPK model.

The input parameters and assumptions used in the development of the PBPK model for asciminib are summarized in Table 1. The overall process of PBPK model development, refinement, and performance verification is outlined in Figure 1.

#### 2.2.1. Physicochemical and Blood-Binding Properties

The molecular weight of asciminib is 449.8 g/mol. Its LogP/LogD_pH=6_._8_, measured using the shake flask method, is 3.9 [8]. Asciminib is a monoprotic weak base with a measured-by-titration pK_a_ value of 4.0 [8]. The mean fraction unbound in plasma (f_u,p_) is 0.027, and the blood-to-plasma (B/P) ratio is 0.80, as determined in vitro [8]. Asciminib plasma protein and blood binding values are concentration independent.

#### 2.2.2. Absorption

The apparent passive permeability of asciminib in the Madin–Darby Canine Kidney Low Efflux (MDCK-LE) cell line is high (22.1 × 10^−6^ cm/s). This was converted by an in-house calibration curve to a human effective permeability of 3.73 × 10^−4^ cm/s, corresponding to a predicted fraction absorbed (f_a_) of 93%, with a percentage recovery of 88% [12].

Although asciminib is a substrate of P-gp and BCRP, intestinal efflux is not expected to limit its absorption at clinically relevant doses. In in vitro transport studies (up to 89.3 μM asciminib) across LLC-PK1/MDR1 cells, a polarized cell line that overexpresses P-gp on the apical membrane, it was concluded that the apparent *K*_m_ value of P-gp-mediated efflux could not be estimated, indicating low P-gp affinity. This was confirmed in a clinical DDI study with the strong P-gp inhibitor, quinidine, where AUC and C_max_ decreased by 13% and 11%, respectively [5].

BCRP-mediated transport was assessed in vitro using C2BBel cells, a Caco-2 subclone lacking P-gp and multidrug resistance protein 2 (MRP2). Kinetic analysis of [^14^C]asciminib (1.1–73 μM) transport across these cells in the apical-to-basolateral direction yielded an apparent mean (±SD) *K_m,app_* of 1.83 ± 2.83 μM. Provided that the estimated gastrointestinal luminal concentration at the lowest clinical dose of asciminib (20 mg) is ~178 μM (which is over 100-fold higher than the *K_m,app_*), BCRP-mediated intestinal efflux is expected to be saturated in vivo.

Asciminib is classified as a biopharmaceutics classification system (BCS) class II compound. As a poorly soluble weak base formulated as a hydrochloride salt, it exhibits, in theory, risk for precipitation during its transfer from the gastric to the intestinal environment. In vitro two-stage dissolution experiments, during which the drug/formulation is pre-exposed to fasted-state simulated gastric fluid (FaSSGF) for a 30 min period, at the end of which, properly concentrated fasted-state simulated intestinal fluid (FaSSIF) is immediately added to FaSSGF [13], were performed to assess the risk of precipitation in vitro. These experiments showed sustained supersaturation for at least 1 h and little precipitation in vitro (<20%) in doses of up to 200 mg. Considering the high passive permeability, this indicates low risk for compromised absorption in vivo, further supporting the assumption of the complete absorption of asciminib in humans in the fasted state.

As the intended application of this work was to primarily support the asciminib NDA and regulatory review regarding DDI and OI predictions, the first-order absorption model within Simcyp was used to reduce the complexity and computation time. Considering the totality of the above presented in vivo and in vitro evidence, the Simcyp user-defined f_a_ was set at 1.00, with a predicted output of 0.96. The absorption rate constant (*k*_a_) in the fasted state was set at 1.3 h^−1^ (% coefficient of variance (CV) = 9.0) consistent with the popPK model [14]. The fraction unbound in the enterocytes (fu_gut_) and the intestinal flow term (Q_gut_) were set at 0.25 and 5.3 (L/h), respectively, based on parameter sensitivity analysis (PSA) across a range of values (0.01–1.00 for fu_gut_ and 0.53–30.0 for Q_gut_). These parameters were optimized to best match the DDI magnitude on C_max_ with midazolam, clarithromycin, and itraconazole. The predicted F_g_ and F_h_ values were 0.82–0.84 and 0.92, respectively, resulting in bioavailability (F) (F = f_a_ × F_g_ × F_h_) value of 0.73.

#### 2.2.3. Distribution

Due to the inclusion of hepatic efflux transporters in Simcyp, the whole-body PBPK model was employed. The permeability liver model (PerL) was activated within Simcyp to incorporate the active transport of asciminib to the bile via BCRP. The volume of distribution at the steady state (V_ss_) was initially predicted using the Rodgers–Rowland method (Method 2). However, the passive diffusion clearance (CL_PD_) in the PerL increases the V_ss_ by moving the drug into the liver. A top-down approach was applied to adjust the K_p_ scalar to visually best fit the model to the distribution phase of the observed clinical PK data. The actual V_ss_ value, considering both the permeability-limited liver and the perfusion-limited liver, is 0.8 L/kg. This is in agreement with the V_ss_ value from preclinical data showing low-to-moderate V_ss_ values (0.5–2.2 L/kg) across animal species.

#### 2.2.4. Metabolism and Excretion

##### In Vitro Studies

In vitro studies demonstrated that asciminib is primarily metabolized by CYP3A4, UGT2B7, and UGT2B17. Kinetic analysis of [^14^C]asciminib metabolism in human liver microsomes (HLMs) showed that glucuronidation accounted for ~65% of the metabolism, followed by oxidative metabolism (~35%), consistent with findings in human hepatocyte incubations [3]. Using enzyme kinetics in recombinant CYP enzymes and scaling based on CYP abundance in HLMs, the relative contributions to oxidative metabolism were estimated as follows: CYP3A4 (96.0%), CYP2J2 (2.08%), CYP2C8 (1.36%), and CYP2D6 (0.574%) [3].

##### Human ADME Study

In the hADME study, the major circulating and urinary metabolite was the direct O-glucuronide of asciminib (M30.5), accounting for 4.93% of the total AUC and 5.4–8.4% of the administered dose. However, M30.5 was not present in feces, likely due to back-conversion to parent asciminib while residing in the gastrointestinal lumen. This is consistent with the in vitro stability study in human feces, in which M30.5 was found to be almost completely hydrolyzed back to asciminib after 20 h.

In the cumulative feces (pooled from 0 to96 h or 0 to 168 h), asciminib accounted for an average value of 56.7% [3]. Due to the unstable nature of the glucuronide in the gastrointestinal tract, the extent to which asciminib was converted via metabolism to the glucuronide vs. direct biliary excretion as unchanged asciminib could not be readily assessed; hence, the percentage of unchanged asciminib (56.7% of the dose) in feces may overestimate the unabsorbed asciminib fraction. From the late-time-point feces profile (96–144 or 144–168 h) and the ratio of asciminib to metabolites, 24% of the parent asciminib detected in feces was estimated to be due conversion of the unstable glucuronide (M30.5) back to the parent asciminib. This suggests a maximum absorption of 56.7%, though this estimate assumes a constant metabolite-to-parent ratio and does not precisely account for direct biliary excretion [3]. In contrast, in vitro permeability studies in MDCK cells estimated *f_a_* to be ~93%, and two-stage dissolution experiments confirmed complete dissolution at doses of up to 200 mg, supporting near-complete absorption in vivo.

Based on alignment between clinical hADME data and in vitro clearance studies, the relative contributions to the total clearance were estimated as follows: UGT mediated (58.3%), CYP mediated (36.6%), and hydrolysis (0.71%), assuming no active biliary secretion. However, as discussed below, the in vivo contribution of UGTs was later adjusted downward to account for BCRP-mediated biliary excretion.

##### Biliary Excretion

Biliary excretion was assumed to contribute to the overall elimination of asciminib, as supported by rat, monkey, and human ADME studies. Active transport to the bile was modeled using the PerL with BCRP kinetic parameters (J_max_ and *K*_m_). Initial estimates of CL_PD_ and transporter kinetic parameters were derived from in vitro Caco-2 cell (C2BBe1) data. The intracellular unbound in vitro constant for BCRP (*K*_m,u,BCRP_) was calculated at 0.142 ± 0.219 μM [15].

The observed slightly more than dose-proportional increase in asciminib exposure was attributed to saturation of the BCRP-mediated hepatic efflux. This is supported by the fact that the in vitro determined *K*_m_ values of all the other major pathways (CYP and UGT, as reported in [3]) were at least 90-fold higher, suggesting a lower likelihood of saturation for those pathways (Table 1). The transport parameters (CL_PD_, J_max_, and *K*_m_) were optimized to visually best fit the training PK dataset following BID dosing in cancer patients (Table 2).

At the lowest clinically tested dose (20 mg BID), the contribution of BCRP to the overall clearance was estimated at approximately 31.1%, assuming no saturation of hepatic efflux at this dose. This was determined by comparing simulated CL/F values with and without BCRP: 4.96 L/h without BCRP versus 7.19 L/h with BCRP. At the highest tested dose (200 mg BID), BCRP saturation was nearly complete, with only an ~5% difference between simulated CL/F values with and without BCRP.

##### Renal Elimination

In urine, unchanged asciminib accounted for 4.4% of the administered radioactive dose, indicating minimal renal clearance of asciminib. The estimated mean renal clearance (CL_r_) for asciminib (1.8 mL/min/1.73 m^2^) was about 56% of the typical value of the glomerular filtration rate when multiplied by the plasma fu_p_ (fu_p_ × GFR = 0.027 × 120 mL/min/1.73 m^2^ = 3.24 mL/min/1.73 m^2^), suggesting no involvement of renal transporters in the elimination process [3].

##### Determination of the Final Fraction Metabolized (fm) and Fraction Transported (ft) to the Total Asciminib Clearance

As stated above, the fractional contribution of the BCRP to the overall elimination of asciminib was estimated at 31.1%. This value was subtracted from the total UGT-mediated clearance (58.3%), resulting in a revised UGT contribution of approximately 27.3%.

Within this adjusted UGT fraction, the relative contributions of UGT1A3/4, UGT2B7, and UGT2B17 were 24.2%, 47.9%, and 27.9%, respectively [3], corresponding to final f_m_ values of ~6.6% for UGT1A3/4, ~13.1% for UGT2B7, and ~7.6% for UGT2B17.

The contributions of CYP enzymes and renal elimination remained unchanged. CYP3A4 accounted for the majority of the CYP-mediated clearance (~35.1%), while minor contributions were attributed to CYP2C8 (0.5%), CYP2D6 (0.2%), CYP2J2 (0.76%), hydrolysis (0.71%), and renal elimination (~4.4%). A summary of the Simcyp output for elimination pathways, with and without BCRP, is provided in Supplementary Table S1.

**Figure 2:**
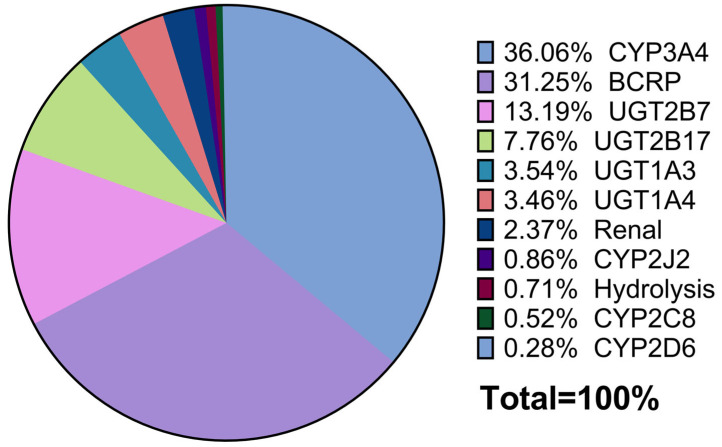
Pie chart of the relative contributions of asciminib clearance pathways.

Using the Simcyp retrograde model, initial estimates of intrinsic clearance (CL_int,u_) were derived based on fractional contributions from recombinant human CYP and UGT enzymes, as well as additional intrinsic clearance in HLMs attributed to hydrolysis. Enzyme kinetic parameters (*K*_m_ and V_max_) were used to establish the model, with V_max_ values back-calculated as the product of CL_int,u_ and *K*_m_ to match the observed f_m_ values to the total clearance. The hydrolysis pathway was assigned a fixed contribution of 0.71% to the total clearance. A schematic representation of the final pathway contributions, as captured by the final PBPK model, is illustrated in Figure 2.

#### 2.2.5. Interaction

##### Inhibition Effects of Asciminib on CYP and UGT Enzymes

The potential of asciminib for the reversible inhibition of CYP and UGT enzymes was studied in vitro in HLM and recombinant enzymes, respectively. In addition, asciminib exhibited no apparent TDI for CYP1A2, CYP2C9, CYP2D6, or CYP3A4/5 at concentrations of up to 50 μM in vitro. All the in vitro inhibition constants, along with their associated variability, can be found in Table 1.

According to the Net Effect Model [9], the static assessment results indicated that asciminib had the potential to be a weak inducer of CYP1A2 and a weak inhibitor of CYP3A, CYP2C9, and UGT1A1 at a dose of 40 mg BID [16]. Static assessment results for the 80 mg QD and 200 mg BID dose levels were also calculated accordingly. All the relevant asciminib compartmental concentrations for the static DDI risk assessments are summarized in Supplementary Table S2 [8,17,18].

##### Induction Effects of Asciminib on CYP Enzymes

Based upon in vitro mRNA induction data, asciminib has the potential to induce CYP1A2 and CYP3A4 (in-house data). With the calculation of the risk using the R_3_ algorithm (at the time of this work, based on FDA 2020 guidance), no induction risk for CYP2C9 and CYP2B6 was anticipated. The induction parameter values used in the PBPK model for CYP1A2 and CYP3A4 were from human hepatocytes. The in vitro measured induction parameters, IndC_50_ (or EC_50_) and Ind_max_ (=E_max_ + 1), for CYP3A4 induction were 2.7 μΜ and 5.4-fold, respectively. The IndC_50_ and Ind_max_ values for CYP3A4 were calibrated with the rifampicin in vitro induction parameters (rifampicin EC_50_ = 0.42 µM and E_max_ = 124-fold) using the respective built-in Simcyp calibrator. After calibration for the positive control, rifampicin (RIF), the IndC_50_ and Ind_max_ for CYP3A4 induction by asciminib were 2.057 µM and 1.53-fold, respectively. Table 1 shows the final calibrated values for asciminib. The CYP1A2 in vitro induction parameters (EC_50_ and Ind_max_) entered into the model were 0.59 µM and 4.5-fold, respectively. Calibration of the induction parameters for CYP1A2 was not conducted due to the lack of reference data.

##### Inhibition Effects of Asciminib on Transporters

In vitro *K*_i_ values for organic cation transporter (OCT)1, organic anion-transporting polypeptide (OATP)1B1, OATP1B3 (hepatic uptake), P-gp, BCRP (hepatic and intestinal efflux), OCT2, organic anion transporter (OAT)1, OAT3, and multidrug and toxic compound extrusion (MATE) transporters (MATE-1, MATE2K) were entered into the PBPK model, and static DDI risk assessments for 40 mg BID, 80 mg QD, and 200 mg BID dose levels were performed.

#### 2.2.6. Model Assumptions and Limitations

To ensure transparency and reproducibility, the key assumptions and limitations underlying the model are outlined below.

##### Contribution of Biliary Secretion via BCRP

The observed more-than-dose-proportional increase in asciminib exposure is attributed to saturation of the BCRP-mediated hepatic efflux. However, this assumption is supported only by in vitro data (e.g., significantly lower *K*_m_ values) and in vivo animal studies. Direct clinical evidence in humans is lacking, as confirmation would require bile duct cannulation—a highly invasive and impractical procedure.

Moreover, in the absence of absolute bioavailability data, the actual fraction of absorbed asciminib remains uncertain. This further complicates the distinction among directly secreted, unabsorbed, and glucuronide-back-converted asciminib.

The uncertainties in the fractional contributions of biliary secretion and glucuronidation and their potential implications for predicting DDIs in which asciminib acts as a victim drug are acknowledged.

##### Enterohepatic Circulation (EHC)

EHC of asciminib, whether via direct secretion or glucuronide back-conversion—was not included in the model: This exclusion is based on the sequestration of asciminib by bile salt micelles in the intestinal lumen, which limits its reabsorption. Supporting in vivo evidence comes from asciminib food-effect studies, where meal- and fat-dependent negative food effects were observed, with exposure reductions of 33% and 70% under low-fat and high-fat fed conditions, respectively.

This sequestration effect was further investigated using an in vitro flux assay, which demonstrated reduced asciminib flux in the presence of elevated bile acid concentrations (FeSSIF). These findings are detailed in Hoch et al. [6].

Consequently, asciminib becomes available for absorption only after bile salt reuptake in the terminal ileum—a region with low permeability due to reduced surface area and tighter epithelial junctions. Reabsorption in this region is, therefore, considered as minimal. Additionally, mean PK profiles from healthy volunteer DDI studies show no evidence of EHC, as reported in Hoch et al. [4,5].

To assess the potential impact of the EHC, a sensitivity analysis was performed by extending the model to simulate an extreme scenario of 100% reabsorption of biliary-secreted asciminib. This led to a less than 15% increase in AUC, which was deemed not clinically relevant and, therefore, excluded from the final model.

### 2.3. Other Compound Victim Drug Files

PBPK models of repaglinide (CYP2C8), *S*-warfarin (CYP2C9) and midazolam (CYP3A4), caffeine (CYP1A2), omeprazole (CYP2C19), and raltegravir (UGT1A1) are available in the Simcyp library (v19.1) with the respective model verification documents. These documents provide verification of the ability of these substrate files to be used for the purpose of predicting DDI with respect to CYP inhibition and, for some, induction and inactivation of the enzyme indicated above.

### 2.4. Other Compound Perpetrator Drug Files

#### 2.4.1. CYP3A4 Perpetrators

PBPK models of the strong (rifampicin) and moderate (efavirenz) CYP3A4 inducers as well as for the strong (clarithromycin) and moderate (fluconazole and erythromycin) CYP3A4 inhibitors are available in the Simcyp library (v19.1) with the respective model verification documents. For another strong CYP3A4 inhibitor, itraconazole, and its hydroxyl metabolite, the respective models verified by IQ were available [19], capturing the PK and DDI profiles after oral administration of the capsule formulation of itraconazole in the fasted state.

#### 2.4.2. Imatinib

A verified PBPK model (in Simcyp v11.0) for imatinib, a CYP3A4, UGT1A3/4, UGT2B17, and BCRP inhibitor, was made available by Filppula et al., 2013, capturing the PK after oral administrations of single and multiple doses of imatinib [20]. In addition to the interaction properties included in the published model, imatinib was found to inhibit the in vitro activities of recombinant UGT1A3 (IC_50_ = 20 μΜ), UGT1A4 (IC_50_ = 15 μΜ), and UGT2B17 (IC_50_ = 0.07 μΜ). The input parameters provided by Filppula et al., 2013 [20], including these UGT in vitro inhibition parameters, were used for the imatinib compound file in Simcyp v19.1, and a similar predictive performance of the imatinib PBPK model in this Simcyp version (compared to Simcyp v11.0) was confirmed.

### 2.5. Clinical Trial Simulation Designs

The following simulation trial designs were applied for PK (Supplementary Table S3 [5,7,21,22]), victim (Supplementary Table S4 [5,6,19,20]), and perpetrator DDI simulations (Supplementary Table S5 [7]), reproducing the trial design of previous clinical pharmacology studies at the asciminib 40 mg dose level in the fasted state. For the simulations of untested scenarios, an age range of 20–55 years old and a female proportion of 0.5 were used for both victim and perpetrator DDI assessments (Supplementary Tables S6 and S7). Simulations for hepatic and renal impairment were based on the designs outlined in Supplementary Tables S8 and S9.

All the simulations were conducted using the final market image (FMI) formulation of asciminib. When available, trial designs were aligned with those of the corresponding reference clinical studies. Virtual populations were selected to closely match the enrolled individuals in the respective clinical trials with regard to disease state, gender ratio, and age. DDI and PK predictions in healthy subjects were performed with the Simcyp North European Caucasian (“NEurCaucasian”) population, whereas for PK predictions in cancer patients, the Simcyp Cancer (“Sim-Cancer”) population was used. PBPK simulations showed minimal differences (<15%) in asciminib PK between these two populations. In this context, and since the clinical DDI trials were performed in HVs, the “NEurCaucasian” population was chosen for the simulation of DDI trials in this report. All the simulated trials were run with a 10 trial × 10 subject (*n* = 100) design.

### 2.6. Evaluation of Predictive Performance and PBPK Model Diagnostics

The predictive performance of the model was assessed by visual predictive checks as well as by comparing predicted and observed plasma concentration values and PK parameters. For this purpose, the ratio (R_pred/obs_) of model-predicted versus observed parameter values was determined (R_pred/obs_ = model-predicted/clinically observed). The predictive accuracy was evaluated on the basis of the ‘twofold’ rule (−0.301 < logR_pred/obs_ < 0.301) [23,24] as well as the more stringent deviation of 25% (−0.097 < logR_pred/obs_ < 0.097). Particularly for the predictions of DDI and OI, the Guest criteria were used to assess model accuracy [25].

As quantitative measures of model performance, the average fold error (AFE) and the absolute average fold error (AAFE) of PK parameters were calculated. For DDI and OI, the geometric mean fold error (GMFE) of PK parameters was used instead:

(1) $$AFE=10^{\frac{1}{n}\sum_{j}^{n}{log}_{10}(\frac{\hat{a_{j}}}{a_{j}})}$$

(2) $$AAFE/GMFE=10^{\frac{1}{n}\sum_{j}^{n}\left|{log}_{10}(\frac{\hat{a_{j}}}{a_{j}})\right|}$$

where *a*_*j*_ and $\hat{a_{j}}$ correspond the observed and the respective predicted PK parameter values of the *j*th clinical PK dataset, and *n* is the number of datasets, respectively. AFE deviation from unity is an indication of over- (AFE > 1) or underprediction (AFE < 1) of the observed data, whereas AAFE is a measure of the absolute error from the true value (or bias of the simulated profile). An AAFE of ≤ 2 is considered to be a successful prediction [26].

### 2.7. Identification of Uncertain Parameters in the Asciminib PBPK Models

In addition to optimizing the BCRP transporter kinetic parameters (J_max_ and *K*_m_) and CL_PD_, the fraction unbound in enterocytes (f_u,gut_) and intestinal blood flow (Q_gut_) were refined using PSA

The initial PBPK model prediction of the clinical DDI with midazolam [4], using a default f_u,gut_ value of 1 and the Simcyp-predicted Q_gut_ value (13.7 L/h), resulted in a geometric mean AUC_inf_ ratio of 1.39 (90% CI: 1.36, 1.43) and a geometric mean C_max_ ratio of 1.33 (1.30, 1.37) for midazolam. The initial PBPK-predicted midazolam DDI ratios slightly overpredicted the observed values (AUC_inf_ ratio = 1.28 and C_max_ ratio = 1.11), and the C_max_ ratio fell slightly outside of the Guest criteria. Although asciminib is both an inhibitor and an inducer of CYP3A4 in vitro, its induction potential was considered as negligible based on the relative induction score (RIS) and PBPK simulations, which predicted <15% and <2% decreases in the midazolam AUC, respectively (Supplementary Table S13 [4]). Thus, the induction potential of asciminib on the PK of midazolam was considered as negligible for the optimization of f_u,gut_ and Q_gut_.

Midazolam is subject to substantial first-pass metabolism by CYP3A after oral administration, showing a PBPK-model-predicted mean F_g_ of 0.62. Therefore, predictability of the midazolam AUC and C_max_ ratios can be affected by the estimation of f_u,gut_ and Q_gut_, which are compound specific. As mentioned in Section 2.2, sensitivity analysis was performed on the asciminib f_u,gut_ value as well as on Q_gut_, with the trial design of the midazolam study (Supplementary Figure S1). Despite that the PSA results showed relatively low impacts of asciminib f_u,gut_ and Q_gut_ values on the midazolam AUC and C_max_ ratios, the DDI was better predicted, with final values of f_u,gut_ (f_u,gut_ = 0.25 and Q_gut_ = 5.3 L/h), which were selected for the established asciminib PBPK model.

In addition, the initial PBPK model prediction of the clinical DDI with *S*-warfarin, using the in vitro determined CYP2C9 K_i,u_ of 0.407 μΜ, resulted in geometric mean AUC_inf_ ratio for *S*-warfarin of 1.04 (90% CI: 1.03, 1.04) and a geometric mean C_max_ ratio of 1.01 (1.01, 1.01). The initial PBPK model underpredicted the observed values (AUC_inf_ ratio = 1.41 and C_max_ ratio = 1.08), with the AUC ratio falling slightly outside the Guest criteria. PSA was performed to identify the CYP2C9 K_i_ value that would be more predictive of the *S*-warfarin clinical DDI, and a final value of 0.03 μM was used in the established asciminib PBPK model (Supplementary Figure S2).

These adjustments (f_u,gut_, Q_gut_, and CYP2C9 K_i_) were considered as important for the top-down optimization of asciminib perpetrator potential toward CYP3A and CYP2C9 and for projecting DDI effects at higher doses.

### 2.8. Applications of the Established Asciminib PBPK Model

Using the established asciminib PBPK model, the victim and perpetrator DDIs as well as the effects of HI and RI were predicted at therapeutic asciminib doses of 80 mg QD and 200 mg BID. PBPK predictions for DDI scenarios that were not tested clinically at the 40 mg dose level are also provided. Precisely, the established asciminib model was applied to predict the DDI effects of strong CYP3A perpetrators (rifampicin, clarithromycin, and itraconazole) and imatinib on the PK of asciminib after oral administration of a single 80 or 200 mg dose. It was also applied to predict the DDI effects of moderate or weak CYP3A perpetrators (efavirenz, fluconazole, and erythromycin) on the PK of asciminib after oral administration of a single 40, 80, or 200 mg dose. Furthermore, it was applied to predict the steady-state effects of asciminib after multiple administrations of 80 mg QD or 200 mg BID on the PKs of sensitive CYP3A4 (midazolam), CYP2C9 (warfarin), and CYP2C8 (repaglinide) substrates as well as of 40 mg BID, 80 mg QD, and 200 mg BID on the PK of sensitive CYP2C19 (omeprazole) and UGT1A1 (raltegravir) substrates. Lastly, the model was applied to extrapolate the effects of mild, moderate, and severe HIs or RIs on the PK of asciminib after a single asciminib oral administration of 80 or 200 mg.

### 2.9. Presentation of Output Parameters

All the systemic PK data were based on plasma concentrations. For PK simulations, the predicted AUC, C_max_, and T_max_ values are shown as arithmetic means and standard deviations (SDs), percentage coefficients of variance (CV%), and medians with ranges, respectively. For DDI and OI predictions, the AUC and C_max_ values and ratios were defined as the geometric mean and 90% confidence interval (CI).

## 3. Results

### 3.1. Performance Verification of the Asciminib Model to Predict the PK in Healthy Subjects and Cancer Patients

The calculated AUC, C_max_, and median T_max_ values, after a single asciminib dose (40 mg) in fasted healthy subjects or as multiple doses (20, 40, 80, 160, and 200 mg BID and 40, 80, and 200 mg QD) in cancer patients, using the established PBPK model, were in line with the observed values (Table 2). The percentage of prediction error (PE%) for AUC and C_max_ was equal to or less than 26% and 45% after single and multiple doses, respectively (AFE/AAFE for AUC 1.14/1.17 and for C_max_ 0.997/1.10). In addition, simulated SD and CV (%) values of C_max_ and AUC values in the healthy subjects and patients were also reasonably well predicted, indicating that the PBPK model captured adequately the population (inter-subject) variability.

**Table 2:** Summary of asciminib pharmacokinetics after a single dose in healthy volunteers or multiple once and twice daily oral administration (20–200 mg) in cancer patients.

Trial ^1^	Dose and Regimen	Mean C_max_ ± SD (CV%) ng/mL		Mean AUC ± SD (CV%) ng·h/mL ^3^		Mean C_trough_ ± SD (CV%) ng/mL ^4^		Median T_max_ [Min, Max] h	
		Observed	Simulated	Observed	Simulated	Observed	Simulated	Observed	Simulated
**Healthy volunteers**									
Clarithromycin DDI control arm [5]	40 mg single dose	567 ± 187 (33)	625 ± 139 (22)	6040 ± 2020 (33.5)	5490 ± 1964 (36)	NA	NA	2.02 [1.00, 3.00]	1.20 [0.86, 1.97]
			%PE ^2^ = 10.2		%PE = −9.11				%PE = −40.6
Rifampicin DDI control arm [5]	40 mg single dose	595 ± 207 (34.7)	627 ± 138 (22)	5870 ± 1720 (29.3)	5520 ± 1996 (36)	NA	NA	2.00 [1.98, 4.00]	1.20 [0.91, 1.97]
			%PE = 5.38		%PE = −5.96				%PE = −40.0
Itraconazole DDI control arm [5]	40 mg single dose	594 ± 225 (37.8)	623 ± 138 (22)	6000 ± 2210 (36.9)	5436 ± 1958 (36)	NA	NA	2.01 [1.93, 3.00]	1.20 [0.86, 1.97]
			%PE = 4.88		%PE = −9.40				%PE = −40.3
Fasted control arm [6]	40 mg single dose	589 ± 220 (37.3)	619 ± 137 (22)	6040 ± 1980 (32.7)	5299 ± 1868 (35)	NA	NA	2.01 [1.00, 5.00]	1.25 [0.95, 2.00]
			%PE = 5.09		%PE = −12.3				%PE = −37.8
HI control arm [7]	40 mg single dose	584 ± 89.0 (15.2)	659 ± 148 (23)	5000 ± 1020 (20.4)	6306 ± 2091 (33)	NA	NA	2.00 [1.00, 4.00]	1.22 [0.90, 2.02]
			%PE = 12.8		%PE = 26.1				%PE = −39.0
RI control arm [7]	40 mg single dose	584 ± 164 (28.0)	696 ± 140 (20)	5720 ± 1530 (26.7)	6904 ± 2279 (33)	NA	NA	2.03 [1.02, 2.05]	1.26 [0.90, 1.94]
			%PE = 19.2		%PE = 20.7				%PE = −37.9
**Cancer patients**									
First in human	20 mg BID Day 1	249 ± 92.6 (37.2)	305 ± 70 (23)	1053 ± 385 (36.5)	1529 ± 433 (28)	NA	NA	2.07 [1.83, 3.10]	1.20 [0.87, 1.86]
			%PE = 22.5		%PE = 45.2				%PE = −42.0
	20 mg BID Day 15	339 ± 108 (31.9)	445 ± 127 (29)	2515 ± 710 (28.2)	3216 ± 1277 (40)	114 ± 61.8 (54.0)	149 ± 90 (61)	2.98 [1.97, 4.07]	1.14 [0.89, 1.71]
			%PE = 31.3		%PE = 27.9		%PE = 30.7		%PE = −61.7
	20 mg BID Day 28	537 ± 544 (101)	445 ± 127 (29)	2977 ± 2165 (72.7)	3216 ± 1277 (40)	128 ± 93.1 (72.8)	149 ± 90 (61)	2.03 [1.25, 6.00]	1.15 [0.86, 1.73]
			%PE = −17.1		%PE = 8.03		%PE = 16.4		%PE = −43.4
	40 mg BID Day 1	653 ± 468 (71.6)	618 ± 145 (24)	2695 ± 1679 (62.3)	3187 ± 925 (29)	NA	NA	2.10 [1.95, 5.62]	1.24 [0.87, 1.90]
			%PE = −5.36		%PE = 18.3				%PE = −41.0
	40 mg BID Day 15	806 ± 365 (45.3)	980 ± 302 (31)	5519 ± 2782 (50.4)	7545 ± 3096 (41)	309 ± 218 (70.6)	385 ± 221 (58)	2.11 [1.97, 4.03]	1.18 [0.89, 1.71]
			%PE = 21.6		%PE = 36.7		%PE = 24.6		%PE = −44.1
	40 mg BID Day 28	873 ± 369 (42.3)	980 *±* 302 (31)	5777 ± 2439 (42.2)	7544 *±* 3097 (41)	308 ± 162 (52.5)	384 ± 221 (58)	2.01 [1.00, 6.00]	1.15 [0.86, 1.73]
			%PE = 12.3		%PE = 30.6		%PE = 24.7		%PE = −42.8
	80 mg BID Day 1	1365 ± 534 (39.1)	1206 *±* 283 (24)	5628 ± 2160 (38.4)	6308 *±* 1788 (28)	NA	NA	2.88 [1.00, 3.93]	1.24 [0.91, 1.90]
			%PE = −11.6		%PE = 12.1				%PE = −57.6
	80 mg BID Day 15	2127 ± 666 (31.3)	1939 ± 572 (29)	11,971 ± 3598 (30.1)	15,100 ± 5897 (39)	1087 ± 723 (66.5)	780 ± 422 (54)	2.13 [2.00, 3.00]	1.18 [0.89, 1.76]
			%PE = −8.84		%PE = 26.1		%PE = −28.2		%PE = −44.6
	80 mg BID Day 28	2165 ± 788 (36.4)	1939 *±* 572 (29)	14,327 ± 6400 (44.7)	15,096 *±* 5896 (39)	1020 ± 576 (56.5)	779 ± 422 (54)	2.02 [1.5, 3.97]	1.19 [0.90, 1.73]
			%PE = −10.4		%PE = 5.37		%PE = −23.6		%PE = −41.1
	160 mg BID Day 1	2923 ± 1545 (52.9)	2508 *±* 614 (24)	13,706 ± 4533 (33.1)	13,360 *±* 3976 (30)	NA	NA	2.10 [0.83, 5.98]	1.24 [0.87, 1.94]
			%PE = −14.2		%PE = −2.52				%PE = −41.0
	160 mg BID Day 15	4327 ± 1368 (31.6)	4373 *±* 1504 (34)	30,577 ± 13,410 (43.9)	35,506 *±* 15,925 (45)	2193 ± 1036 (47.2)	1962 ± 1161 (59)	2.17 [1.00, 3.92]	1.18 [0.85, 1.71]
			%PE = 1.06		%PE = 16.1		%PE = −10.5		%PE = −45.6
	160 mg BID Day 28	4809 ± 1587 (33.0)	4373 *±* 1505 (34)	32,768 ± 11,949 (36.5)	35,495 *±* 15,934 (45)	2559 ± 899 (35.1)	1960 ± 1162 (59)	2.02 [1.87, 3.03]	1.17 [0.86, 1.73]
			%PE = −9.07		%PE = 8.32		%PE = −23.4		%PE = −42.1
	200 mg BID Day 1	3646 ± 1161 (31.8)	3275 *±* 790 (24)	16,788 ± 4964 (29.6)	17,646 *±* 5329 (30)	NA	NA	2.03 [0.95, 7.28]	1.28 [0.91, 2.15]
			%PE = −10.2		%PE = 5.11				%PE = −36.9
	200 mg BID Day 15	5700 ± 1782 (31.3)	6052 *±* 2277 (38)	45,641 ± 13,252 (29.0)	50,649 *±* 24,819 (49)	3191 ± 1391 (43.6)	2910 ± 1852 (64)	2.10 [0.50, 4.00]	1.22 [0.89, 1.84]
			%PE = 6.18		%PE = 11.0		%PE = −8.81		%PE = −41.9
	200 mg BID Day 28	6069 ± 2447 (40.3)	6050 *±* 2278 (38)	40,639 ± 18,474 (45.5)	50,622 *±* 24,826 (49)	3137 ± 1899 (60.5)	2906 ± 1853 (64)	2.00 [0.90, 7.03]	1.19 [0.90, 1.81]
			%PE = −0.31		%PE = 24.6		%PE = −7.36		%PE = −40.5
	80 mg QD Day 1	1253 ± 448 (35.8)	1301 *±* 312 (24)	5780 ± 2043 (35.3)	6874 *±* 2054 (30)	NA	NA	2.06 [1.13, 6.00]	1.22 [0.88, 1.93]
			%PE = 3.83		%PE = 18.9				%PE = −40.8
	80 mg QD Day 15	1595 ± 551 (34.5)	1587 *±* 466 (29)	14,702 ± 4219 (28.7)	17,541 *±* 8323 (47)	227 ± 97.7 (43.1)	303 ± 262 (86)	2.15 [1.02, 4.37]	1.18 [0.88, 1.85]
			%PE = −0.50		%PE = 19.3		%PE = 33.5		%PE = −45.1
	80 mg QD Day 28	1826 ± 422 (23.1)	1587 ± 466 (29)	15,633 ± 4070 (26.0)	17,544 ± 8328 (47)	208 ± 84.4 (40.7)	303 ± 262 (86)	2.00 [0.95, 4.10]	1.19 [0.90, 1.87]
			%PE = −13.1		%PE = 12.2		%PE = 45.7		%PE = −40.5
	120 mg QD Day 1	2199 ± 619 (28.2)	1942 *±* 455 (23)	9543 ± 2795 (29.3)	10,333 *±* 3021 (29)	NA	NA	2.04 [1.13, 7.65]	1.22 [0.88, 1.97]
			%PE = 11.7		%PE = 8.28				%PE = −40.2
	120 mg QD Day 15	2405 ± 748 (31.1)	2396 *±* 715 (30)	21,924 ± 6222 (28.4)	26,948 *±* 13,403 (50)	342 ± 174 (50.9)	481 ± 454 (94)	2.03 [0.98, 4.00]	1.22 [0.88, 1.89]
			%PE = −0.37		%PE = 22.9		%PE = 40.6		%PE = −39.9
	120 mg QD Day 28	2547 ± 750 (29.5)	2396 *±* 717 (30)	21,829 ± 6703 (30.7)	26,956 *±* 13,457 (50)	332 ± 146 (43.8)	480 ± 457 (95)	2.00 [1.00, 3.17]	1.22 [0.90, 1.91]
			%PE = −5.93		%PE = 23.5		%PE = 44.6		%PE = −39.0
	200 mg QD Day 1	3963 ± 1323 (33.4)	3271 *±* 724 (22)	17,234 ± 5648 (32.8)	17,482 *±* 4867 (28)	NA	NA	2.00 [1.08, 4.02]	1.22 [0.88, 1.97]
			%PE = −17.5		%PE = 1.44				%PE = −39.0
	200 mg QD Day 15	4228 ± 1532 (36.3)	4152 *±* 1166 (28)	40,612 ± 16,291 (40.1)	48,302 *±* 22,663 (47)	787 ± 493 (62.7)	928 ± 774 (83)	2.05 [1.00, 4.00]	1.18 [0.88, 1.89]
			%PE = −1.80		%PE = 18.9		%PE = 17.9		%PE = −42.4
	200 mg QD Day 28	4502 ± 1768 (39.3)	4152 ± 1168 (28)	39,144 ± 16,171 (41.3)	48,309 ± 22,708 (47)	523 ± 318 (60.8)	927 ± 776 (84)	2.02 [2.00, 3.00]	1.19 [0.90, 1.91]
			%PE = −7.77		%PE = 23.4		%PE = 77.2		%PE = −41.1
Phase III	40 mg BID Day 15	1010 ± 419 (41.3)	1030 ± 358 (35)	6070 ± 2090 (34.5)	8062 ± 3813 (47)	324 ± 139 (43)	422 ± 284 (67)	1.97 [0.98, 3.33]	1.16 [0.87, 1.71]
			%PE = 1.98		%PE = 32.8		%PE = 30.2		%PE = −41.1
**AFE**			**1.00**		**1.14**		**1.13**		
**AAFE**			**1.10**		**1.17**		**1.29**		

AFE: average fold error; AAFE: absolute average fold error; HI: hepatic impairment; NA: not applicable; RI: renal impairment. ^1^ The actual trial demographics, including the number of subjects, age range, and proportion of females, were used. The simulated trials consisted of 10 trials of 10 subjects (*n* = 100), with the age range and proportion of females matching the actual demographics of the respective clinical studies. The virtual population model used was the North European Caucasian (NEurCaucasian) model. ^2^ %PE, (%) calculated prediction error = [(predicted value − observed value)/observed value] × 100. ^3^ AUC is reported as mean AUC_0–8 h_ for Day 1 and as mean AUC_tau_ (AUC_ss_) for Day 15 and Day 28. ^4^ C_trough_ is reported only for Day 15 and Day 28.

**Figure 3:**
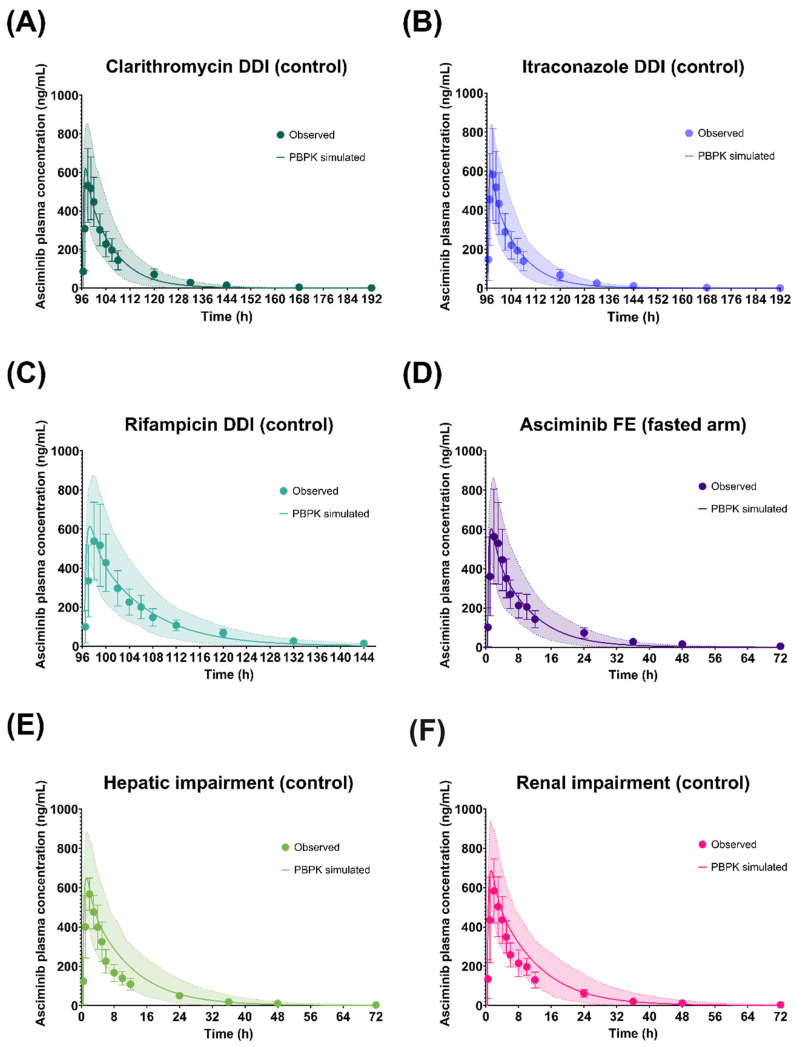
Simulated and observed plasma concentration–time profiles of asciminib after oral administration of a 40 mg single dose in the fasted state in the healthy volunteer control arms of the (**A**) Clarithromycin DDI, (**B**) Itraconazole DDI, (**C**) Rifampicin DDI, (**D**) food effect (fasted arm), (**E**) hepatic impairment, and (**F**) renal impairment studies. The solid lines and shaded areas are the arithmetic mean simulated population PK profiles and the 5th–95th percentiles. The symbols and error bars are the observed arithmetic mean asciminib plasma concentrations and standard deviations, respectively.

**Figure 4:**
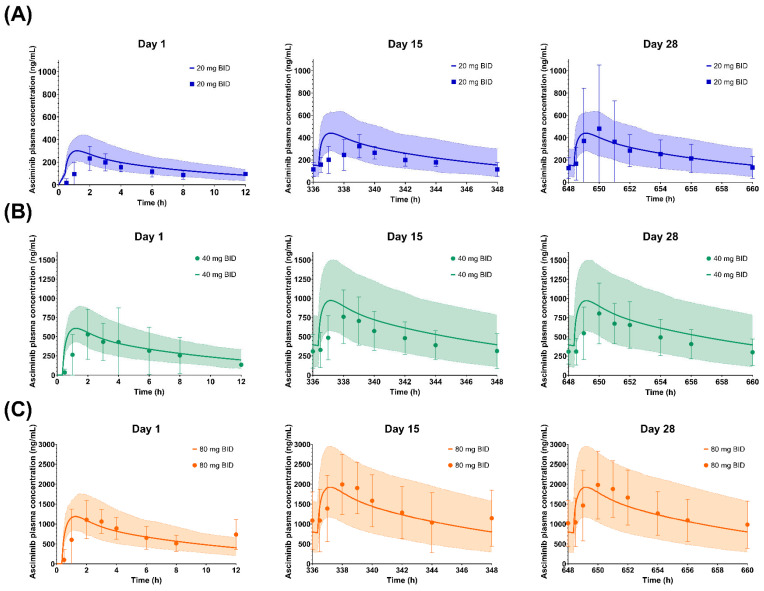

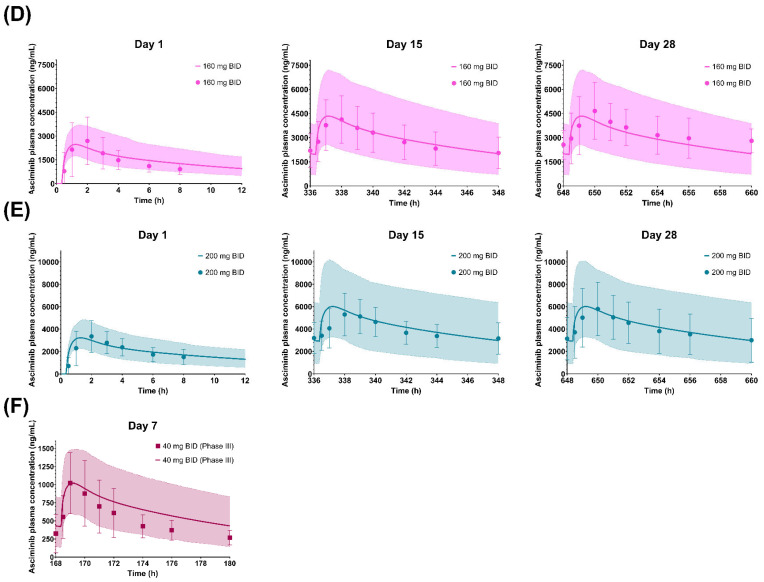
Simulated and observed plasma concentration–time profiles of asciminib on Days 1, 15, and 28 after dosing of (**A**) 20 mg BID, (**B**) 40 mg BID (Phase I data), (**C**) 80 mg BID, (**D**) 160 mg BID, and (**E**) 200 mg BID as well as on Day 7 after a dosing of (**F**) 40 mg BID (Phase III data). The solid lines and shaded areas are the arithmetic mean simulated population PK profiles and the 5th–95th percentiles. Only the days that full PK profiles were collected are presented in the figure. The symbols and error bars are the observed arithmetic mean asciminib plasma concentrations and standard deviations, respectively.

**Figure 5:**
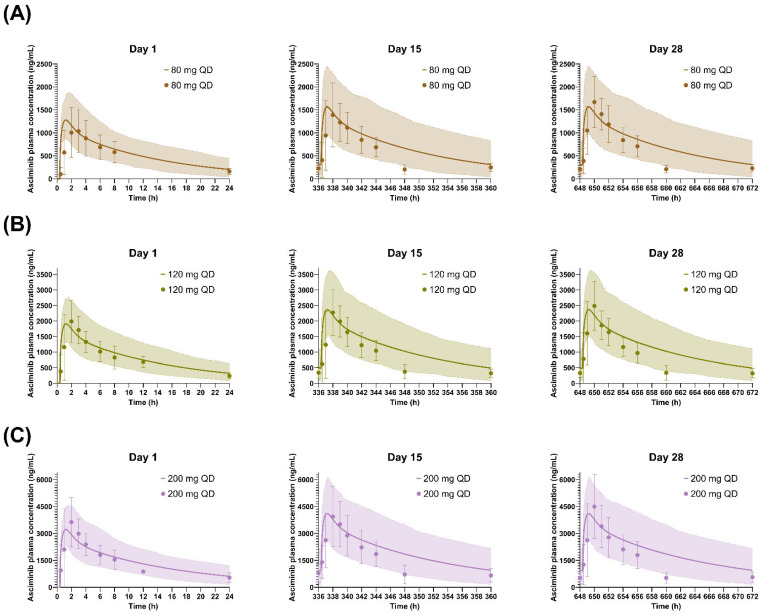
Simulated and observed plasma concentration–time profiles of asciminib on Days 1, 15, and 28 after QD dosing of (**A**) 80 mg, (**B**) 120 mg, and (**C**) 200 mg. The solid lines and shaded areas are the arithmetic mean simulated population PK profiles and the 5th–95th percentiles. Only the days that full PK profiles were collected are presented in the figure. The symbols and error bars are the observed arithmetic mean asciminib plasma concentrations and standard deviations, respectively.

As shown in Figure 3, Figure 4 and Figure 5, the respective simulations of the mean plasma concentration profiles of asciminib over time in healthy subjects (40 mg single dose) as well as in patients (BID and QD multiple doses) were comparable to the corresponding clinical observations.

### 3.2. Performance Verification of the Asciminib Model to Predict the Victim DDI Potential in Healthy Volunteers

#### 3.2.1. Clarithromycin (Strong CYP3A Inhibitor)

**Figure 6:**
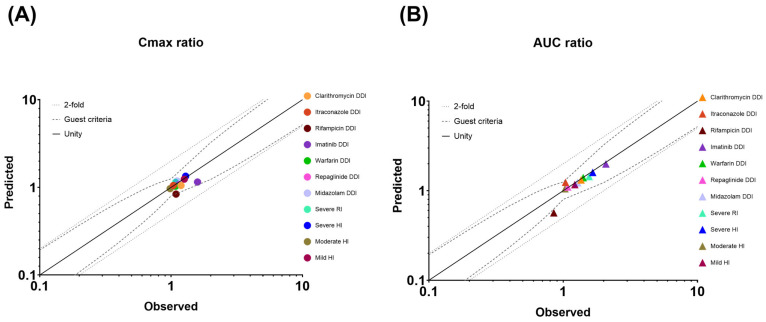
Plots of the predicted versus observed geometric mean (**A**) C_max_ and (**B**) AUC ratios of drug–drug interactions (DDIs) and organ impairment (OI). Solid, dotted, and dashed lines represent the unity line, the twofold deviation from unity, and the Guest criteria upper and lower bands, including 20% intra-subject variability, respectively. Solid colored circles represent a DDI or an OI study.

The geometric means of the AUC_inf_ and C_max_ ratios of asciminib (40 mg single dose on day 5), following oral administration of clarithromycin (500 mg BID for 8 days) in fasted HVs, were predicted to be 1.32 and 1.05, respectively (Table 3 and Figure 6). The PBPK model predicted adequately well the corresponding clinical study observations, in which the observed AUC_inf_ and C_max_ ratios of asciminib were 1.36 and 1.19, respectively [5]. The prediction errors of the geometric mean AUC_inf_ and C_max_ ratios were −2.94% and −11.8%, respectively, being within the Guest criteria. In addition, further in vitro work excluded the potential of clarithromycin inhibiting UGT2B7, an effect that would have been neglected by the current clarithromycin PBPK model, ensuring that the DDI magnitude is solely due to CYP3A4 inhibition.

#### 3.2.2. Itraconazole Capsule (Strong CYP3A Inhibitor)

The geometric mean AUC_inf_ ratio of asciminib (40 mg single dose on day 5), following oral administration of itraconazole capsule (200 mg QD for 8 days) in fasted HVs, was predicted to be 1.24 (Table 3 and Figure 6). The simulated results over-predicted the corresponding clinical study observations, in which the AUC_inf_ ratio was found to be 1.04 [5]. However, the prediction error of the interaction on the geometric mean AUC_inf_ ratio was only 19.2%. The predicted C_max_ ratio was 1.05 versus the observed value of 1.04, and the prediction error was less than 1% and within the Guest criteria. The predicted AUC_inf_ ratio fell slightly outside the Guest criteria when no variability was assumed but within the Guest criteria when 20% intra-subject variability was considered based upon the results of healthy volunteers’ studies (Figure 6).

#### 3.2.3. Rifampicin (Strong CYP3A Inducer)

The geometric mean AUC_inf_ and C_max_ ratios of asciminib (40 mg single dose on day 5), following oral administration of rifampicin (600 mg QD for 6 days) in fasted HVs, were predicted to be 0.566 and 0.838, respectively (Table 3 and Figure 6). The PBPK model overpredicted the induction effect of rifampicin, when compared to the corresponding clinical study observations, in which the AUC_inf_ and C_max_ ratios were found to be 0.851 and 1.09, respectively [5]. The prediction errors of the interaction on the geometric mean AUC_inf_ and C_max_ ratios were −33.5% and −23.1%, respectively. Both predicted AUC_inf_ and C_max_ ratios fell slightly outside the Guest criteria when no variability was assumed; however, the C_max_ ratio was within the Guest criteria when 20% intra-subject variability was considered based upon the results of healthy volunteers’ studies. Rifampicin, when given as single dose, has been identified as a BCRP inhibitor [27]. However, the in vitro inhibition constant for BCRP has only been included in the Simcyp ‘SV-Rifampicin-SD’ file but not in the ‘SV-Rifampicin-MD’ compound file. To explore whether the potential inhibition of BCRP by rifampicin could be an underlying mechanism for the slight overprediction of the induction effect, additional simulations were performed using the BCRP *K*_i_ (=12.54 μM) value from the ‘SV-Rifampicin-SD’ library compound file. However, the inclusion of BCRP inhibition had only increased the geometric mean AUC_inf_ ratio from 0.566 to 0.568. PSA on the BCRP *K*_i_ value indicated minimal impact of this parameter on the AUC_inf_ and C_max_ ratio even when a 100-fold lower value was used.

#### 3.2.4. Imatinib (a CYP3A4, BCRP, UGT1A3/4, and UGT2B17 Inhibitor)

Since imatinib serves as both a substrate and an inhibitor of BCRP, the in vitro measured BCRP IC_50_ (IC_50,BCRP_ = 0.94 μM), which was determined using a cellular assay based on nominal concentrations, might underestimate its BCRP inhibition potency [28]. Using the initial imatinib compound file with the literature BCRP IC_50_ value (IC_50,BCRP_ = 0.94 μM), both the observed geometric mean AUC (AUC ratio = 2.08) and C_max_ ratios (C_max_ ratio = 1.59) of asciminib of the DDI study with imatinib [29] were underpredicted (PBPK-simulated AUC ratio = 1.56 and C_max_ ratio = 1.14). Based on PSA, it was shown that a 10-fold lower IC_50_ value for BCRP inhibition by imatinib could better capture the values observed in the clinical DDI and, thus, it was used in the final compound file (Supplementary Figure S3). Using vesicles carrying BCRP, lower IC_50_ values were confirmed, ranging from 0.08 to 0.29 µM, depending on the substrate used [30]. Using the updated imatinib compound file, the PBPK model predicted an AUC_inf_ ratio of 1.99 for asciminib, which was in line with the observed DDI (Table 3). However, the C_max_ ratio was still underpredicted (C_max_ ratio observed = 1.59 vs. predicted = 1.15). Nevertheless, both predicted AUC_inf_ and C_max_ ratios of asciminib, using either the initial or the updated imatinib compound file, were within the Guest criteria (Figure 6). PBPK predictions with both the initial (BCRP IC_50_ = 0.94 μM) and the updated (BCRP IC_50_ = 0.094 μM) imatinib compound file are summarized in Supplementary Table S12 [6,20].

### 3.3. Performance Verification of the Asciminib Model to Predict the Perpetrator DDI Potential in Healthy Volunteers

#### 3.3.1. Midazolam (CYP3A Substrate)

In vitro asciminib is both a reversible inhibitor and an inducer of CYP3A. Nevertheless, PBPK simulations with and without the CYP3A induction by asciminib showed that the predicted CYP3A interaction is almost exclusively driven by CYP3A inhibition, whereas induction contributed less than 2% to the midazolam AUC ratio (Supplementary Table S13). These results are consistent with the calculated relative induction score (RIS) of 0.070, which also indicated a low CYP3A4 induction potential by asciminib (corresponding to a ~14% CYP3A4-sensitive CYP3A substrate AUC reduction). Using the midazolam Simcyp library compound file (SV-Midazolam’) model, the geometric means of the AUC_inf_ and C_max_ ratios of midazolam (4 mg p.o., single dose on day 3), following oral administration of asciminib (40 mg BID for 5 days) in HVs, were predicted to be 1.23 and 1.18, respectively (Table 3 and Figure 6). PBPK predictions were in line with the corresponding clinical study observations, in which the ratios of AUC_inf_ and C_max_ were 1.28 and 1.11, respectively [4]. Both predicted AUC_inf_ and C_max_ ratios were within the Guest criteria (Figure 6) and with a prediction error of less than 7.5% (Table 3).

#### 3.3.2. S-Warfarin (CYP2C9 Substrate)

The CYP2C9 in vitro inhibition constant of asciminib had been previously optimized based on the PSA of *S*-warfarin DDI AUC_inf_ and C_max_ ratios (Supplementary Figure S2). The geometric mean of the AUC_inf_ and C_max_ ratios of *S-*warfarin (2.5 mg p.o., single dose on day 3), following oral administration of asciminib (40 mg BID for 8 days) in HVs, were predicted to be 1.40 and 1.03, respectively (Table 3 and Figure 6). PBPK predictions were in line with the corresponding clinical study observations, in which the AUC_inf_ and C_max_ ratios of warfarin were 1.41 and 1.08 [4]. Both predicted AUC_inf_ and C_max_ ratios were within the Guest criteria (Figure 6) and with a prediction error of less than 5% (Table 3).

#### 3.3.3. Repaglinide (a CYP2C8 and CYP3A4 Substrate)

The geometric mean of the AUC_inf_ and C_max_ ratios of repaglinide (0.5 mg single dose on day 3), following oral administration of asciminib (40 mg BID for 3 days) in HVs, were predicted to be 1.10 and 1.07, respectively (Table 3 and Figure 6). PBPK predictions were in line with the corresponding clinical study observations, in which the AUC_inf_ and C_max_ ratios of repaglinide were 1.08 and 1.14, respectively [4]. Both predicted AUC_inf_ and C_max_ ratios were within the Guest criteria (Figure 6).

#### 3.3.4. Performance Verification of the Asciminib Model to Predict the Effect of Impaired Hepatic Function

In the Simcyp version at that time (v19.1), changes in the activity of UGT enzymes due to HI were not considered. Since UGT enzymes contribute to the metabolism of asciminib, for severe HI, a ‘modified Sim-Cirrhosis CP-C’ population, accounting for the reduced activity of UGT2B7 and UGT1A4 in such patients, was also used. To the best of our knowledge, no literature sources with regards to UGT enzyme activity in CP-A and CP-B were available at the time this work was completed. Using the ‘Sim-Cirrhosis CP-A’, ‘CP-B’, and ‘CP-C’ library populations, the PBPK model predicted the geometric mean C_max_ and AUC_inf_ ratios in mild, moderate, and severe HI within 40% and 30% of the observed values (mild: AUC_inf_ ratio = 1.22, C_max_ ratio = 1.26; moderate: AUC_inf_ ratio = 1.03, C_max_ ratio = 0.98; severe: AUC_inf_ ratio = 1.66, C_max_ ratio = 1.29) [7], respectively (Supplementary Table S15 [7,10] and Figure S4 [10]).

#### 3.3.5. Performance Verification of the Asciminib Model to Predict the Effect of Impaired Renal Function on the PK

Using the ‘Sim-Renal GFR_less_30’ file, the PBPK model predicted the geometric mean C_max_ and AUC_inf_ ratios for severe renal impairment within 25% and 40% of the observed values, respectively (Supplementary Table S16 [7,11] and Figure S5). The PBPK model predicted no increase in the AUC in renal impairment (AUC_inf_ ratio ≅ 1.0). As the levels of plasma proteins and, thus, the asciminib fraction unbound in plasma (fu_p_) might be impacted in subjects with impaired renal functions, fu_p_ was identified as a sensitive parameter. Single ex vivo measurements of fu_p_ at 2 h post dose in subjects with normal and severely impaired functions showed no differences (ex vivo fu_p_ normal = 0.0125 vs. 0.0127 in severe impairment) in plasma protein binding. However, as this was only a single time-point measurement, it might not be reflective of the in vivo situation. Thus, sensitivity analysis of fu_p_ on the PK of asciminib was performed and showed that the observed renal impairment effect could be better predicted with a lower fu_p_ of 0.018 (Supplementary Figure S6). Indeed, PBPK simulations using an fu_p_ value of 0.018 predicted the geometric mean AUC_inf_ and C_max_ ratios to be 1.44 and 1.14, respectively, which were within 10% of the observed values (AUC_inf_ ratio = 1.55 and C_max_ ratio = 1.08) [7]. In addition, simulations in subjects with mild and moderate renal impairment are provided (Supplementary Table S16). As no clinical data were available for these populations and for consistency purposes, simulations were performed with both fu_p_ values of the established PBPK model (fu_p_ = 0.027) and the adjusted one (fu_p_ = 0.018) for severe renal impairment.

Overall, the PBPK model predicted well the DDI or OI C_max_ and AUC ratios with GMFEs of 1.10 and 1.09, respectively (Table 3).

### 3.4. PBPK Model Applications

#### 3.4.1. Predictions of Victim DDI Potential in Untested Clinical Scenarios with the Validated Asciminib PBPK Model

##### Clarithromycin and Itraconazole (Strong CYP3A Inhibitors)

**Figure 7:**
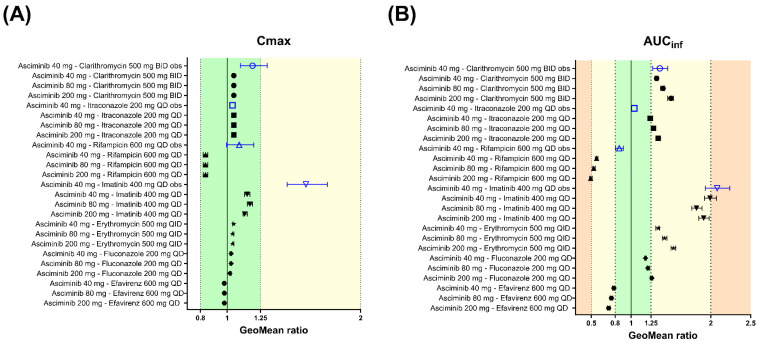
Forest plots to compare the DDI effects of perpetrators at the steady state on the PK of asciminib after 40, 80, and 200 mg oral single doses. Open blue triangles and solid black circles represent the observed and PBPK-predicted geometric mean DDI ratios, respectively, for (**A**) C_max_ and (**B**) AUC_inf_. The error bars represent the 90% confidence interval around the geometric mean ratio. The light-green-, yellow-, and orange-colored zones correspond to DDI ratio ranges of 0.80–1.25, 0.50–0.80, or 1.25–2.00 and 0.20–0.50 or 2.00–5.00, indicating not clinically relevant, weak, and moderate effects, respectively. The observed data are from Hoch et al., 2022 [5,6].

The geometric mean of the AUC_inf_ and C_max_ ratios of asciminib (80 or 200 mg single dose on day 5), following oral administration of clarithromycin (500 mg BID for 8 days) in HVs, were predicted to be 1.40, 1.50 and 1.05, 1.05, respectively (Figure 7 and Supplementary Table S10 [5]). In addition, the effect of oral co-administration of clarithromycin (500 mg BID) with asciminib (40 mg BID, 80 mg QD, or 200 mg BID), both starting at day 1 and for a total duration of 14 days was simulated and the predicted geometric mean AUC and C_max_ ratios were 1.57, 1.56, 1.77 and 1.34, 1.20, 1.49, respectively (Figure 7 and Supplementary Table S10). The PBPK model predicted slightly different AUC and C_max_ ratios between the single-dose and steady-state DDIs. The increased ratios in the steady state can be partly explained by the fact that asciminib itself has the potential for the reversible inhibition of CYP3A4, especially at 200 mg BID, which, in turn, would lead to higher exposure of clarithromycin and a stronger CYP3A4 inhibitory effect on asciminib. PBPK simulations to assess the effect of clarithromycin 500 mg BID on the PK of asciminib at 200 mg BID, without including CYP3A inhibition by asciminib, predicted the geometric mean AUC and C_max_ ratios to be 1.69 and 1.44, respectively. Moreover, the AUC of clarithromycin at steady state was ~20% lower when the asciminib CYP3A inhibitory effect was not included (geometric mean AUC_ss_ values of clarithromycin with and without CYP3A inhibition by asciminib were 39,103 ng·h/mL and 30,842 ng·h/mL, respectively). In addition, at 200 mg BID after multiple dosing, saturation of hepatic BCRP is expected, and thus the relative contribution of CYP3A4 to the clearance of asciminib is likely to become more important.

The geometric mean of the AUC_inf_ and C_max_ ratios of asciminib (80 and 200 mg single dose on day 5), following oral administration of itraconazole (200 mg QD for 8 days) in HVs, were predicted to be 1.28, 1.34 and 1.05, 1.05, respectively (Figure 7 and Supplementary Table S10). After oral co-administration of itraconazole 200 mg QD with asciminib (40 mg BID, 80 mg QD, or 200 mg BID), both starting at day 1 and for a total duration of 14 days, the predicted AUC and C_max_ ratios were 1.37, 1.48, 1.52 and 1.24, 1.20, 1.36, respectively (Figure 7 and Supplementary Table S10). The PBPK model predicted slightly different AUC and C_max_ ratios between the single dose and steady-state DDI. Like clarithromycin, the increased ratios in steady state can be assigned to a mutual CYP3A4 inhibition between asciminib and itraconazole and the saturation of the biliary secretion pathway via the BCRP efflux of asciminib, resulting in a higher fractional contribution of CYP3A4 to asciminib clearance.

##### Fluconazole and Erythromycin (Moderate CYP3A Inhibitors)

The geometric mean of the AUC_inf_ and C_max_ ratios of asciminib (40, 80 and 200 mg single dose on day 5), following oral administration of fluconazole (200 mg QD for 8 days) in HVs, were predicted to be 1.18, 1.21, 1.26 and 1.03, 1.03, 1.02, respectively (Figure 7 and Supplementary Table S11). After oral co-administration of fluconazole 200 mg QD with asciminib (40 mg BID, 80 mg QD, or 200 mg BID), both starting at day 1 and for a total duration of 14 days, the predicted geometric mean AUC and C_max_ ratios were 1.40, 1.41, 1.49 and 1.24, 1.15, 1.32, respectively (Figure 7 and Supplementary Table S11).

In addition to CYP3A, fluconazole also has the potential for reversible inhibition of UGT2B7, which is the main UGT enzyme contributing to the metabolism of asciminib. Exploratory simulations investigating the combined (CYP3A and UGT2B7) versus the individual CYP3A inhibition effect on the PK of asciminib are also provided (Supplementary Table S11). Based on the PBPK model estimates, the interaction between fluconazole and asciminib would be mainly driven by CYP3A inhibition, while inhibition of UGT2B7 would be expected to contribute less than 15% to the overall AUC increase.

The geometric mean of the AUC_inf_ and C_max_ ratios of asciminib (40, 80 and 200 mg single dose on day 5), following oral administration of erythromycin (500 mg QID for 8 days) in HVs, were predicted to be 1.34, 1.42, 1.53 and 1.05, 1.04, 1.04, respectively (Figure 7 and Supplementary Table S11). After oral co-administration of erythromycin 500 mg QID with asciminib (40 mg BID, 80 mg QD, or 200 mg BID), both starting at day 1 and for a total duration of 14 days, the predicted geometric mean AUC and C_max_ ratios were 1.60, 1.59, 1.77 and 1.36, 1.22, 1.50, respectively (Figure 7 and Supplementary Table S11).

##### Rifampicin (a Strong CYP3A Inducers)

The geometric mean of the AUC_inf_ and C_max_ ratios of asciminib (80 and 200 mg single dose on day 5), following oral administration of rifampicin (600 mg QD for 6 days) in HVs, were predicted to be 0.531, 0.492 and 0.837, 0.838, respectively (Figure 7 and Supplementary Table S10). After oral co-administration of rifampicin 600 mg QD with asciminib (40 mg BID, 80 mg QD, or 200 mg BID), both starting at day 1 and for a total duration of 14 days, the predicted geometric mean AUC and C_max_ ratios were 0.413, 0.480, 0.366 and 0.575, 0.765, 0.531, respectively (Figure 7 and Supplementary Table S10). The PBPK model predicted slightly different AUC and C_max_ ratios between the single-dose and steady-state DDIs. The increased induction effect in the steady state can be explained by the fact that hepatic BCRP is likely saturated at 200 mg BID, and, thus, the relative contribution of CYP3A4 is likely to become more important.

##### Efavirenz (a Moderate CYP3A Inducer)

The geometric mean of the AUC_inf_ and C_max_ ratios of asciminib (40, 80 and 200 mg p.o. single dose on day 5), following oral administration of efavirenz (600 mg QD for 8 days) in HVs, were predicted to be 0.783, 0.752, 0.718 and 0.978, 0.979, 0.979, respectively (Figure 7 and Supplementary Table S11). After oral co-administration of efavirenz 600 mg QD with asciminib (40 mg BID, 80 mg QD, or 200 mg BID), both starting at day 1 and for a total duration of 14 days, the predicted geometric mean AUC and C_max_ ratios were 0.674, 0.676, 0.620 and 0.821, 0.911, 0.772, respectively (Figure 7 and Supplementary Table S11).

##### Imatinib (a CYP3A4, BCRP, UGT1A3/4, and UGT2B17 Inhibitor)

Using the imatinib compound file based on the published Simcyp model by Filppula et al., 2013 [20], which was updated for BCRP IC_50_ (IC_50_ = 0.094 μM), the geometric means of the AUC_inf_ and C_max_ ratios of asciminib (80 and 200 mg p.o. single doses on Day 5), following oral administration of imatinib (400 mg QD for 8 days) in HVs, were predicted to be 1.17, 1.13, 1.82, and 1.91, respectively (Figure 7 and Supplementary Table S12). After oral co-administration of imatinib 400 mg QD with asciminib (40 mg BID, 80 mg QD, or 200 mg BID), both starting at day 1 and for a total duration of 14 days, the predicted geometric mean AUC and C_max_ ratios were 2.09, 2.08, 2.14 and 1.72, 1.46, 1.81, respectively (Figure 7 and Supplementary Table S12).

As imatinib is an inhibitor of CYP3A (reversible and time dependent), BCRP, UGT1A3/4, and UGT2B17, PBPK simulations exploring further the relative contributions of the individual pathways to the inhibitory effect of asciminib are provided (Supplementary Table S12). It was shown that at an asciminib dose of 40 mg, BCRP, CYP3A, and UGT enzymes contributed almost equally to the overall interaction effect with imatinib, which was in line with the respective fractions metabolized (f_m_) or transported (f_t_). At the same time, based on the PBPK estimates (when only BCRP inhibition was considered), there was a decrease in the contribution of BCRP with increasing doses of asciminib, which was even more pronounced at the 200 mg BID dose of asciminib at the steady state, with an AUC ratio of 1.01, indicating full saturation of BCRP at this dose level.

#### 3.4.2. Predictions of Perpetrator DDI Potential in Untested Clinical Scenarios with the Validated Asciminib PBPK Model

##### Midazolam (a CYP3A4 Substrate)

**Figure 8:**
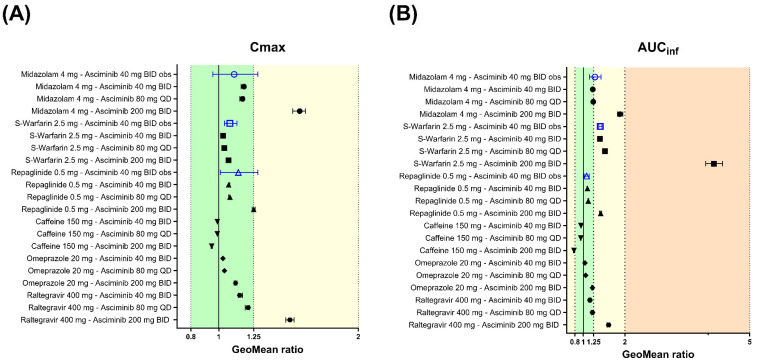
Forest plots to compare the DDI effects of asciminib 40 mg BID, 80 mg QD, or 200 mg BID at the steady state on the PK of CYP/UGT-sensitive substrates after a single oral dose. Open blue triangles and solid black circles represent the observed and PBPK predicted geometric mean DDI ratios, respectively, for (**A**) C_max_ and (**B**) AUC_inf_. The error bars represent the 90% confidence interval around the geometric mean ratio. The light-green-, yellow-, and orange-colored zones correspond to DDI ratio ranges of 0.80–1.25, 0.50–0.80, or 1.25–2.00 and 0.20–0.50 or 2.00–5.00, indicating not clinically relevant, weak, and moderate effects, respectively. The observed data are from Hoch et al. [4].

The geometric mean of the AUC_inf_ and C_max_ ratios of midazolam (4 mg p.o., single dose on day 3), following oral administration of asciminib (80 mg QD and 200 mg BID for 5 days) in HVs, were predicted to be 1.24, 1.88 and 1.17, 1.58, respectively (Figure 8 and Supplementary Table S13). After oral co-administration of midazolam 4 mg QD with asciminib (40 mg BID, 80 mg QD, or 200 mg BID), both starting at day 1 and for a total duration of 14 days, the predicted geometric mean AUC and C_max_ ratios were almost identical to those in the single dose design ones (Supplementary Table S13).

##### S-Warfarin (a CYP2C9 Substrate)

The geometric means of AUC_inf_ and C_max_ ratios for *S*-warfarin (2.5 mg p.o., single dose on day 3) with co-administration of asciminib at therapeutic oral doses of 80 mg QD and 200 mg BID were predicted to be 1.52 and 4.14 for the AUC_inf_ ratio and 1.04, 1.07 for the C_max_ ratio (Figure 8 and Supplementary Table S13). After oral co-administration of *S*-warfarin 2.5 mg QD with asciminib (40 mg BID, 80 mg QD, and 200 mg BID), both starting at day 1 and for a total duration of 35, 35 and 63 days (until the substrate reached the steady state), the predicted AUC ratios were 1.57, 1.57, and 4.41, and the C_max_ ratios were 1.39, 1.37, and 3.38, respectively (Figure 8 and Supplementary Table S13).

##### Repaglinide (a CYP2C8, CYP3A4 and OATP1B Substrate)

The geometric means of the AUC_inf_ and C_max_ ratios for repaglinide (0.5 mg p.o., single dose at Day 3) with co-administration of asciminib at therapeutic oral doses of 80 mg QD and 200 mg BID were predicted to be 1.12 and 1.42 for the AUC_inf_ ratio and 1.08 and 1.25 for the C_max_ ratio (Figure 8 and Supplementary Table S13). After oral co-administration of repaglinide 0.5 mg QD with asciminib (40 mg BID, 80 mg QD, and 200 mg BID), both starting at day 1 and for a total duration of 14 days, the predicted AUC and C_max_ ratios were identical to those in the single dose design trials (Supplementary Table S13). Exploratory PBPK predictions for the effects of asciminib on the individual metabolic pathways of repaglinide are also provided in Supplementary Table S13.

##### Caffeine (a CYP1A2 Substrate)

The PBPK platform Simcyp was verified for the use of DDI risk assessments due to the reversible inhibition potential on CYP1A2. Running simulations of the CYP1A2 induction effect of asciminib on the PK of caffeine is likely an extended use of the PBPK modeling platform. Irrespectively, DDI simulations were performed to explore the combined (inhibition and induction) effect of asciminib (p.o. 40 mg BID, 80 mg QD, and 200 mg BID) on caffeine (150 mg p.o., single dose at day 3) and the geometric mean AUC_inf_ and C_max_ ratios were predicted to be 0.945, 0.941, 0.777 and 0.989, 0.989, 0.949, respectively (Figure 8 and Supplementary Table S14). After oral co-administration of caffeine 150 mg QD with asciminib (40 mg BID, 80 mg QD, or 200 mg BID), both starting at day 1 and for a total duration of 14 days, the predicted geometric mean AUC and C_max_ ratios were 0.901, 0.901, 0.645 and 0.964, 0.965, 0.863, respectively (Figure 8 and Supplementary Table S14). Exploratory PBPK simulations differentiating between the asciminib induction and inhibition effects on caffeine showed that CYP1A2 inhibition has no effect, regardless of the asciminib dose or regimen examined, and that the interaction is solely driven by CYP1A2 induction (Supplementary Table S14).

##### Omeprazole (a CYP2C19 Substrate)

The geometric mean of the AUC_inf_ and C_max_ ratios of omeprazole (20 mg p.o., single dose on day 3), following oral administration of asciminib (40 mg BID, 80 mg QD and 200 mg BID for 5 days) in HVs, were predicted to be 1.04, 1.06, 1.22 and 1.03, 1.04, 1.12, respectively (Figure 8 and Supplementary Table S14). After oral co-administration of omeprazole 20 mg BID with asciminib (40 mg BID, 80 mg QD, or 200 mg BID), both starting at day 1 and for a total duration of 14 days, the predicted geometric mean AUC and C_max_ ratios were 1.07, 1.06, 1.41 and 1.04, 1.04, 1.23, respectively (Figure 8 and Supplementary Table S14).

##### Raltegravir (a UGT1A1 Substrate)

The geometric mean of the AUC_inf_ and C_max_ ratios of raltegravir (400 mg p.o., single dose on day 3), following oral administration of asciminib (40 mg BID, 80 mg QD and 200 mg BID for 5 days) in HVs, were predicted to be 1.16, 1.22, 1.61 and 1.15, 1.21, 1.51, respectively (Figure 8 and Supplementary Table S14). After oral co-administration of raltegravir 400 mg BID with asciminib (40 mg BID, 80 mg QD, or 200 mg BID), both starting at day 1 and for a total duration of 14 days, the predicted geometric mean AUC and C_max_ ratios were 1.16, 1.09, 1.62 and 1.16, 1.09, 1.54, respectively (Figure 8 and Supplementary Table S14). In both designs, raltegravir was dosed 1h after the administration of the perpetrator, asciminib, as this was found to maximize the inhibition effect on the substrate.

#### 3.4.3. Predictions of Hepatic Impairment Potential

The established PBPK model was used to predict the effects of mild, moderate, and severe HI on the PK of asciminib after oral administration of single doses of 80 and 200 mg. The PBPK model predicted less than 20% differences in the AUC_inf_ and C_max_ ratios (impaired vs. control) among the 40, 80, and 200 mg dose levels, regardless of the severity of the HI, indicating that the effect of impaired hepatic function on the PK of asciminib was dose independent. All the results are summarized in Supplementary Table S15.

#### 3.4.4. Predictions of Renal Impairment Potential

The established PBPK model was used to predict the effects of mild, moderate, and severe renal impairment on the PK of asciminib after oral administration of a single dose of 80 and 200 mg. The PBPK model predicted less than 15% differences in the AUC_inf_ and C_max_ ratios (impaired vs. control) among the 40, 80, and 200 mg dose levels, regardless of the severity of the renal impairment, indicating that the effect of impaired renal function on the PK of asciminib is dose independent. All the results are summarized in Supplementary Table S16.

## 4. Discussion

A nonlinear, whole-body, permeability-limited liver PBPK model was developed and validated for asciminib as a CYP3A4, UGT, and BCRP substrate and perpetrator of several CYP and UGT enzymes. The PBPK model for asciminib was developed and refined by applying a stepwise ‘middle out’ modeling approach by leveraging in vitro, in silico, and in vivo data. The established PBPK model was able to robustly reproduce the plasma concentration–time profiles and captured adequately the confirmed over-proportional increase in asciminib exposure with increasing doses. In addition, the model captured reasonably well the observed between-subject variability, as represented by the respective coefficients of variation (%CVs) of the PK parameters. Development of a PBPK model with this rigor to mechanistically explain nonlinearity in exposure, is a key factor in the acceptance of a PBPK model, particularly to bridge clinical pharmacology studies from one dose to all marketed doses.

The model was further verified to predict the interaction effects of strong CYP3A4 inhibitors (clarithromycin and itraconazole) and inducers (rifampicin) as well as the combined effect of imatinib, a CYP3A4, UGT1A3/4, UGT2B17, and BCRP inhibitor. The prediction of the DDI with clarithromycin confirms the f_mCYP3A4_, whereas the prediction of imatinib DDI further supported the proposed elimination pathways. The overprediction of the induction by rifampicin could not be fully explained, even though its potential to inhibit BCRP was explored. Additional simulations using the BCRP *K*_i_ value (=12.54 μM) from the ‘SV-Rifampicin-SD’ library compound file and PSA on the BCRP *K*_i_ value indicated minimal impacts of this parameter on the AUC_inf_ and C_max_ ratios, even when a 100-fold lower value was used.

The established asciminib model supported the NDA of Scemblix^®^ for DDI and OI, and it was the subject of the integrated clinical pharmacology review by the U.S. FDA. PBPK simulations informed the drug product label and were used in lieu of clinical pharmacology studies. The PBPK applications, FDA’s assessments, and impacts on the drug product label are summarized in Table 4.

The agency considered that the PBPK model could adequately describe the PK of asciminib, following oral administration of a 40 mg single dose in HVs and multiple dosing 20–200 mg in cancer patients [29].

### 4.1. Extrapolation of the Effects of Strong and Moderate CYP3A Inhibitors on Asciminib (80 and 200 mg Doses)

Based on PBPK estimates in victim DDI assessments, a weak interaction effect (less than twofold) is expected with strong and moderate CYP3A4 inhibitors, regardless of the dose or regimen.

However, due to the complexity of elimination pathways and uncertainties related to the potential for saturation, the lack of intravenous data, and the relative contributions of the different pathways, the predictive ability of the model as a substrate was questioned [29]. The PBPK model was not accepted for quantitative predictions of the magnitude of the effects of moderate and strong CYP3A inhibitors. Nevertheless, it was actually used to inform dosing recommendations in the prescribing information, when asciminib at 200 mg was co-administered with CYP3A inhibitors. In addition, on the drug product label of Scemblix^®^, there are no label restrictions or dosing adjustments required [31], indicating that the evidence provided by the PBPK model could have been supportive.

### 4.2. Extrapolation of the Effects of Strong and Moderate CYP3A Inducers on Asciminib (80 and 200 mg Doses)

The PBPK model overpredicted the induction effect of rifampicin on asciminib (40 mg) from the actual clinical DDI study, with prediction errors in the AUC_inf_ and C_max_ ratios of –33.5% and −23.1%, respectively. An AUC decrease of between 47 and 63%, depending on the dose or regimen examined, was predicted with the strong CYP3A4 inducer, rifampicin, whereas the respective AUC reduction due to the effect of the moderate inducer, efavirenz, ranged between 22% and 38%.

The overprediction of the effect of rifampin on asciminib after oral administration of a 40 mg single dose could be considered as a worst-case scenario in terms of efficacy. However, the agency had concerns about the potential induction of other enzymes (e.g., UGT enzymes) or transporters involved in the elimination of asciminib at doses higher than 40 mg. Considering the high regulatory impact application and the above-mentioned uncertainties, the model was considered as inadequate to extrapolate the effects of CYP3A inducers on the PK of asciminib at 80 or 200 mg [29].

This led to a post-marketing requirement by the FDA to characterize the effects of a strong CYP3A inducer at a higher asciminib dose, e.g., 200 mg. To fulfill this requirement, the effect of the strong CYP3A inducer, phenytoin, 100 mg three times daily (TID), on the PK of asciminib after oral administration of a single 200 mg dose was investigated in a dedicated clinical DDI study. In presence of phenytoin 100 mg TID, the C_max_ and AUC_inf_ of asciminib 200 mg single dose were reduced by 22% and 34%, with geometric mean C_max_ and AUC_inf_ ratios of 0.780 (90% CI: 0.718–0.847) and 0.662 (90% CI: 0.624–0.703), respectively. The established asciminib PBPK model predicted the geometric mean C_max_ and AUC_inf_ ratios of the phenytoin DDI to be 0.859 (90% CI: 0.842–0.876) and 0.624 (90% CI: 0.600–0.649), which are within 10% of the observed values. These results further support the proposed f_m_ and the robustness of the PBPK as a substrate of CYP3A.

### 4.3. Extrapolation of the Effects of Imatinib on Asciminib (80 and 200 mg Doses)

The PBPK model predicted a decreasing imatinib DDI magnitude with increasing asciminib dose due to the BCRP saturation at higher asciminib doses (AUC ratio range = 1.82–1.99). However, due to the above-discussed uncertainties, the PBPK model was not accepted by the agency, which is reflected on the drug product label by the statement: “*Concomitant use of imatinib with SCEMBLIX at 200 mg twice daily has not been fully characterized*.”

### 4.4. Extrapolation of Asciminib Effects at 80 mg QD and 200 mg BID on CYP3A-, CYP2C9-, and CYP2C8-Sensitive Substrates

The PBPK model, with asciminib as a perpetrator (40 mg BID), predicted accurately the effects on the PK of midazolam, warfarin, and repaglinide, after PSA and optimization of f_u,gut_ and CYP2C9 K_i,u_ for midazolam and warfarin DDIs, respectively. The robustness of the model to predict perpetrator DDI effects at the 40 mg BID dose, along with the accurate prediction of asciminib PK across doses and regimens, provides confidence to predict other marketed doses.

Based on the PBPK model’s estimates for asciminib at 40 mg BID or 80 mg QD and regardless of the trial design, a >1.25-fold interaction is expected only for CYP2C9 substrates, whereas no effect was predicted for CYP2C8 substrates. At both 40 mg BID and 80 mg QD doses of asciminib, a marginal effect on midazolam (a CYP3A4-sensitive substrate) was predicted. However, at 200 mg BID, a moderate interaction with CYP2C9 and weak interactions with CYP3A4 and CYP2C8 substrates were predicted. The robustness of the model to predict perpetrator DDI effects at the 40 mg BID dose provides confidence (along with the accurate prediction of asciminib PK) to predict other marketed doses.

The PBPK analysis was considered to be adequate to extrapolate the interactions of midazolam, *S*-warfarin, and repaglinide with asciminib 80 mg QD or 200 mg BID. The predicted increases in C_max_ and AUC for midazolam, *S*-warfarin, and repaglinide when co-administered with asciminib 80 mg QD or 200 mg BID were included in the prescribing information. Based on the PBPK simulations (Supplementary Table S13), close monitoring of adverse events and avoidance of co-administration were recommended when Scemblix 80 mg total daily dose and 200 mg BID is co-administered with CYP3A4 substrates, where minimal concentration changes may lead to serious adverse reactions [31]. A similar avoidance statement was proposed for all the asciminib therapeutic doses when administered with CYP2C9 substrates where minimal concentration changes may lead to serious adverse reactions [31].

Repaglinide is a substrate of CYP2C8 (f_m_ ≈ 0.7) and CYP3A (f_m_ ≈ 0.3) and is subject to OATP1B1-mediated hepatic uptake, whereas asciminib is an inhibitor of CYP2C8, CYP3A, and OATP1B1 in vitro. The interaction effect of asciminib on the PK of repaglinide may be caused by the net inhibition effects on CYP2C8, CYP3A, and OATP1B1. DDI simulations were conducted considering the interaction effect of asciminib in all these pathways and compared to observed data (Supplementary Table S13). A weak interaction effect (1.25 ≥ AUC ratio < 2) is predicted for asciminib 200 mg BID, but no inhibition potential (AUC ratio < 1.25) is predicted for asciminib at an 80 mg total daily dose. Exploratory predictions of the effects of asciminib on the individual pathways of repaglinide were also conducted. In addition to repaglinide, the effect of asciminib on rosiglitazone, which is a dual substrate of CYP2C8 (f_m_ ≈ 0.6) and CYP2C9 (f_m_ ≈ 0.3), was also explored. A weak interaction effect (1.25 ≥ AUC ratio < 2) is predicted for asciminib 200 mg BID, but no inhibition potential (AUC ratio < 1.25) is predicted at an 80 mg total daily dose. An exploratory prediction of the effect of asciminib only on the CYP2C8 pathway of rosiglitazone was also conducted. The non clinically meaningful and weak interactions of asciminib at 80 mg QD and 200 mg BID, respectively, predicted by the PBPK model with CYP2C8 substrates allowed their use without labeling restrictions (Supplementary Table S15) [31].

The PBPK predicted DDI ratios at 80 mg QD and 200 mg BID for midazolam, S-warfarin, repaglinide, and rosiglitazone appear on the drug product label of Scemblix^®^ in lieu of clinical data.

### 4.5. Prediction of Asciminib Effects at 40 mg BID, 80 mg QD, and 200 mg BID on CYP2C19-Sensitive Substrates

Omeprazole is a substrate of CYP2C19 (f_m_ ≈ 0.9) and CYP3A (f_m_ ≈ 0.1, CYP2C19 normal metabolizers). No clinical DDI study has been conducted with asciminib and a CYP2C19 substrate. A lower value for the inhibition constant than the in vitro value (≈14-fold lower) was needed to recover the clinical DDI effect with the CYP2C9 substrate S-warfarin. Therefore, a PSA for the in vitro CYP2C19 inhibition parameter as a part of the risk analysis was recommended by the FDA. Based upon the experimental mean and SD values of IC50 for the coefficient of variation (CV) did not exceed 42%, and, thus, a 50% variability as a worst-case scenario was selected. The CYP2C19 K_i,u_ (=IC50,u/2) value and the PSA range were 1.5 µM and 0.75–2.25 µM, respectively. A K_i,u_ value 10-fold lower than that of the in vitro CYP2C19 was also tested. Asciminib is predicted to be a weak inhibitor of CYP2C19 at 200 mg BID (1.25 ≥ AUC ratio < 2), but no inhibition potential is predicted at an 80 mg total daily dose. In the context of PSA using a 10-fold lower value for CYP2C19 K_i,u_, asciminib is predicted to be a weak inhibitor of CYP2C19 at an 80 mg total daily dose and a moderate inhibitor at 200 mg BID [29].

In summary, the DDI simulations for asciminib 40 mg BID or 80 mg QD, and regardless of single or multiple dosing of the substrate, predicted a weak interaction (1.25 ≥ AUC ratio < 2). Based on PBPK simulations, the use of CYP2C19 substrates as co-medications is allowed without any restrictions and a statement that asciminib may reversibly inhibit CYP2C19 at concentrations reached at 200 mg BID was included on the label [31].

### 4.6. Prediction of Asciminib Effects at 40 mg BID, 80 mg QD, and 200 mg BID on UGT1A1-Sensitive Substrates

Raltegravir is a substrate of UGT1A1 (f_m_ ≈ 0.9). No clinical DDI study has been conducted with asciminib and a UGT1A1 substrate. The raltegravir model assumes the drug is only metabolized by UGT1A1 and 9% eliminated by renal clearance and has been partially verified by Simcyp, as clinical data on UGT1A1 poor metabolizers are not available. Additionally, all the non-renal clearance of raltegravir was assumed to be mediated by UGT1A1, omitting the involvement of UGT1A9 in the metabolism of raltegravir. Nevertheless, this means that the raltegravir model used is more conservative to evaluate the effect of a UGT1A1 inhibitor.

After oral co-administration of raltegravir 400 mg BID with asciminib 40 mg BID, 80 mg QD or 200 mg BID (both starting at day 1 and for a total duration of 14 days), the predicted geometric mean AUC and Cmax ratios were 1.16, 1.09, 1.62 and 1.16, 1.09, 1.54, respectively (Supplementary Table S14). The IVIVE of the UGT1A1 inhibition effect has not been established, and, as a result, PSA of the UGT1A1 inhibition constant for the predicted DDI effect with raltegravir was conducted. In a manner similar to that for omeprazole, 50% variability of the UGT1A1 Ki value was the worst-case scenario. The UGT1A1 K_i,u_ value and the sensitivity analysis range were 0.35 µM and 0.175–0.525 µM, respectively. The PBPK analysis indicated that asciminib has the potential for a positive interaction with a UGT1A1 substrate (AUC ratio > 1.25).

In summary, the DDI risk assessment indicated that the potential for the interaction of asciminib with a UGT1A1 substrate cannot be excluded. Based on PBPK simulations, the use of UGT1A1 substrates as co-medications is allowed without any restrictions and a statement that asciminib may reversibly inhibit UGT1A1 at total daily dose of 80 mg and 200 mg BID was included on the label [31].

### 4.7. Prediction of Asciminib Effects at 40 mg BID, 80 mg QD, and 200 mg BID on CYP1A2-Sensitive Substrates

Caffeine is a substrate of CYP1A2 (f_m_ ≈ 0.9). No clinical DDI study has been conducted with asciminib and a CYP1A2 substrate. The caffeine model has been validated, by Simcyp, for the use of DDI predictions caused by the reversible inhibition of CYP1A2 but not by induction. However, asciminib is both a reversible inhibitor and an inducer of CYP1A2. DDI simulations were conducted considering the combined (inhibition and induction) effect of asciminib (40 mg BID, 80 mg QD, and 200 mg BID) on the PK of caffeine (single 150 mg oral dose at day 3 or multiple dosing for 14 days). Simulations, conducted to differentiate between asciminib’s induction and inhibition effects, showed that the interaction would be the result of CYP1A2 induction, with a dose dependency, but a minimal CYP1A2 inhibition effect (regardless of the asciminib or caffeine dosing regimen). The PBPK analysis was considered as adequate to estimate the interaction potential of asciminib with sensitive CYP1A2 substrates, such as caffeine, allowing their use without any restriction on the drug product label [29], since the in vitro-to-in vivo extrapolation (IVIVE) for CYP1A2 induction has not been established and limited positive correlations have been reported so far [32]. PSA on the CYP1A2 induction parameters was requested to support the DDI risk assessment. The PSA range was defined as the mean experimental value ±1 x SD of the respective parameter. The in vitro determined values were IndC50 = 0.59 µM (SE = 0.13 µM) and Emax 3.5-fold (SD = 0.47), and the PSA ranges for IndC50 and Indmax were 0.36–0.82 µM and 3.9–5.1-fold, respectively. A 10-fold reduction in IndC50 was also tested by the FDA’s reviewer, indicating that the potential for the interaction (assumed as an AUCR of < 0.8) of asciminib with a sensitive CYP1A2 substrate cannot be excluded. However, this is considered as an overly conservative estimate, and no mention for CYP1A2 substrates is presented on the drug product label [31].

### 4.8. Extrapolation of the Effect of Hepatic and Renal Impairment on Asciminib (80 mg and 200 mg oses)

Simulations to predict the PK of asciminib in subjects with impaired liver or renal functions were also performed, and the model was able to describe the observed ratios with a prediction error of less than 40%. The PBPK model was applied to predict the effects of OI on the PK of asciminib after single doses at 80 and 200 mg. The PBPK simulations indicated that the magnitude of the OI would be dose independent, showing less than 20% differences across doses for the same degree of impairment severity. The maximal effect was predicted in severely impaired subjects at 200 mg, and it was a weak effect, with 92% and 60% increases in exposure in HI and RI, respectively (Supplementary Tables S15 and S16).

For both HI and RI, the PBPK model was deemed to be inadequate for the agency to extrapolate the effects of OI on the PK of asciminib at 80 mg or 200 mg due to the underprediction of the OI effects at 40 mg (Supplementary Tables S16 and S17) and uncertainties in the relative contributions of elimination pathways. Nonetheless, according to the drug product label [31], no dose adjustments are required for either HI or RI, even at 200 mg BID.

Overall, the PBPK analyses were mainly intended to predict DDIs in cancer patients. However, DDI simulations were performed using the HV population model because the clinical DDI studies were performed in HVs. This assumes that (1) the PK of asciminib in HVs is similar to that in cancer patients and (2) there are no impactful differences in the enzyme activity or abundance (related to asciminib clearance) between healthy and cancer subjects. The first assumption may be supported by comparable C_max_ (601 and 537 ng/mL) and AUC (6520 and ≈ 5800–6100 ng/mL·h) values between HVs and cancer patients at a 40 mg dose [29]. For the second assumption, despite evidence that CYP enzyme activity (or abundance) may be altered in cancer patients [33], the contribution of CYP enzymes to the total asciminib clearance may not be substantial enough to see exposure differences. All in all, the interaction potential of asciminib is not expected to be substantially different between healthy and cancer patients.

## 5. Conclusions

The established asciminib PBPK model was applied in lieu of clinical studies to support the new drug application of asciminib. Victim and perpetrator DDIs, as well as the effects HI or RI, were predicted at asciminib therapeutic doses of 80 mg QD and 200 mg BID. PBPK predictions for DDI scenarios that were not tested clinically at the 40 mg dose level are also provided.

The PBPK model was well accepted by the U.S. FDA for its ability to predict the nonlinear PK of asciminib in wide dose ranges of 20–200 mg BID and 80–200 mg QD. Particularly for asciminib as a perpetrator of CYP and UGT enzymes, PBPK predictions were used in lieu of clinical trials and were included on the drug product label. At the victim and organ impairment levels, the complexity of asciminib elimination pathways prevented full acceptance of the model. However, the effects of CYP3A4 inhibitors on the PK of asciminib, based on PBPK, are reflected on the drug product label, and no dose adjustment or additional clinical studies were requested for HI or RI. Considering the complex and nonlinear nature of asciminib PK, which would have led to additional clinical DDI and organ impairment studies at 80 mg and 200 mg, the PBPK predictions are estimated to have replaced at least 10 clinical studies. Overall, this work demonstrates the potential of PBPK modeling and simulations in streamlining drug development, informing regulatory decisions, and supporting labeling recommendations. More importantly, this example underscores the value of robust PBPK modeling and simulation in lieu of clinical pharmacology studies.

## Figures and Tables

**Table 1:** Input parameters for the asciminib PBPK model.

Input Parameter	Description	Units	Mean Value (SD)	Reference
**Physicochemical and blood binding properties**				
MW	Molecular weight	g/mol	449.85	
Log P	Octanol–water partition (titration)/logD pH 6.8 (titration)	-	3.9	[8]
Compound type	Acid, base, or neutral	-	Monoprotic base	
pK_a_		-	4.0	[8]
B/P ratio				[8]
	Blood to plasma ratio	-	0.8 (0.18)	
		
fu_p_	Fraction unbound in plasma	-	0.027 (0.011)	[8]
fu_p RI_	Adjusted fraction unbound in renal impairment		0.018	PSA optimized to fit the PK of subjects with severe RI
**Absorption**				
Absorption model	First order absorption model			
Fasted state				
f_a_	Fraction available from dosage form	-	1	
%CV (f_a_)	Coefficient of variation (f_a_)	-	9.0	Preliminary popPK analysis (%CV for *k*_a_)
*k*_a_	Absorption rate constant	1/h	1.3	Optimized for PK fit
%CV (*k*_a_)	Coefficient of variation (*k*_a_)	-	9.0	Preliminary popPK analysis
tlag	Absorption lag time	h	0.374	Preliminary popPK analysis
%CV (tlag)	Coefficient of variation (tlag)	-	0.4	Preliminary popPK analysis
f_u,gut_	Unbound fraction in enterocytes	-	0.25	PSA on midazolam DDI
Q_gut_	Nominal flow in gut model	L/h	5.3	PSA on midazolam DDI
CV (Q_gut_)	Coefficient of variation (Q(gut))	%	30	Default
P_eff,man_	Effective permeability in man	10^−4^ cm/s	3.729 ^1^	User input (calculated from in-house calibration)
MDCK-LE permeability	Passive permeability (apical to basolateral)	10^−6^ cm/s	22.1 ^1^	In-house data
MDCK-LE reference permeability (negative control)	Passive permeability of aztreonam	10^−6^ cm/s	0.24 ^1^	In-house data
MDCK-LE reference permeability (positive control)	Passive permeability of propranolol	10^−6^ cm/s	36.04 ^1^	In-house data
**Distribution**				
Distribution model	Full PBPK model (permeability-limited liver)			
V_ss_ prediction method	Rodgers–Rowland (Method 2)			
V_ss_	Volume of distribution at steady state	L/kg	0.8	Predicted
K_p_ scalar	Scalar applied to all predicted tissue K_p_ values		0.025	Optimized for PK fit
**Enzyme/transporter phenotyping**				
**Enzyme (recombinant)**				
V_max_ (CYP3A4)	Maximum rate of elimination	pmol/min/pmol of CYP	3.8	Aim for f_m_ = 0.351
*K*_m,u_ (CYP3A4) ^3^	Michaelis–Menten constant	μM	15.7 (0.88)	[3]
V_max_ (CYP2C8)	Maximum rate of elimination	pmol/min/pmol CYP	0.136	Aim for f_m_ = 0.005
*K*_m,u_ (CYP2C8) ^4^	Michaelis–Menten constant	μM	7.6 (0.91)	[3]
V_max_ (CYP2D6)	Maximum rate of elimination	pmol/min/pmol CYP	0.736	Aim for f_m_ = 0.002
*K*_m,u_ (CYP2D6) ^5^	Michaelis–Menten constant	μM	30.7 (3.6)	[3]
V_max_ (CYP2J2)	Maximum rate of elimination	pmol/min/pmol CYP	0.355	Aim for f_m_ = 0.0076
*K*_m,u_ (CYP2J2) ^6^	Michaelis–Menten constant	μM	0.694 (0.051)	[3]
V_max_ (UGT1A3)	Maximum rate of elimination	pmol/min/pmol UGT	1.73	Aim for f_m_ = 0.033 ^2^
*K*_m,u_ (UGT1A3) ^7^	Michaelis–Menten constant	μM	12.9 (2.1)	In-house data
V_max_ (UGT1A4)	Maximum rate of elimination	pmol/min/pmol UGT	0.73	Aim for f_m_ = 0.033 ^2^
*K*_m,u_ (UGT1A4) ^7^	Michaelis–Menten constant	μM	12.9 (2.1)	In-house data
V_max_ (UGT2B7)	Maximum rate of elimination	pmol/min/pmol UGT	2.04	Aim for f_m_ = 0.131 ^2^
*K*_m,u_ (UGT2B7) ^7^	Michaelis–Menten constant	μM	12.7 (2.0)	In-house data
V_max_ (UGT2B17)	Maximum rate of elimination	pmol/min/pmol UGT	17.7	Aim for f_m_ = 0.076 ^2^
*K*_m,u_ (UGT2B17) ^7^	Michaelis–Menten constant	μM	9.41 (1.8)	In-house data
**Transporter (Liver)**				
CL_PD_	Passive diffusion clearance	mL/min/10^6^ hepatocytes	0.06	Optimized for PK fit
J_max_ (BCRP)	In vitro maximum rate of transporter mediated efflux	pmol/min/10^6^ cells	0.2782	Optimized for PK fit
*K*_m_ (BCRP)	Michaelis–Menten constant	μM	0.0070865	Optimized for PK fit In vitro intracellular K_m_ (BCRP) = 0.142 μM (In-house data)
**Other distribution and elimination properties**				
In vivo CL				
CL_r_	Renal clearance in 20–30-year-old healthy male	mL/min/1.73 m^2^	1.8	Aim for f_e_ = 0.025 (equal to 0.108 L/h)
In vitro CL				
HLM CL_int_, liver (unbound)	Additional undefined HLM Clint, liver	µL/min/mg	0.65	Aim for f_m_ = 0.0071 (hydrolysis)
CV HLM CL_int_, liver	% Coefficient of variation HLM Clint, liver	-	30	Default
**Interaction**				
**CYP/UGT inhibition (reversible)**				
IC_50,u_ (CYP1A2)	Unbound ABL001 concentrations estimated to inhibit probe substrate reaction by 50%	µM	20.8 ^8^	In-house data
K_i,u_ (CYP1A2)	Unbound inhibition constant	µM	10.4	as K_i,u_ = IC_50,u_/2
IC_50,u_ (CYP2A6)	Unbound ABL001 concentrations estimated to inhibit probe substrate reaction by 50%	µM	87.1 ^8^	In-house data
K_i,u_ (CYP2A6)	Unbound inhibition constant	µM	43.6	as K_i,u_ = IC_50,u_/2
K_i,u_ (CYP2B6)	Unbound inhibition constant	µM	2.62 ^9^ (0.438)	In-house data
K_i,u_ (CYP2C8)	Unbound inhibition constant	µM	0.466 ^9^ (0.0866)	In-house data
K_i,u_ (CYP2C9)	Unbound inhibition constant	µM	0.03	Optimized based on PSA with Warfarin DDI AUC and C_max_ ratios (initial value 0.407 +/− 0.0595)
IC_50,u_ (CYP2C19)	Unbound ABL001 concentrations estimated to inhibit probe substrate reaction by 50%	µM	3 ^8^	In-house data
K_i,u_ (CYP2C19)	Unbound inhibition constant	µM	1.5	as K_i,u_ = IC_50,u_/2
IC_50,u_ (CYP2D6)	Unbound ABL001 concentrations estimated to inhibit probe substrate reaction by 50%	µM	17 ^8^	In-house data
K_i,u_ (CYP2D6)	Unbound inhibition constant	µM	8.5	as K_i,u_ = IC_50,u_/2
IC_50,u_ (CYP2E1)	Unbound ABL001 concentrations estimated to inhibit probe substrate reaction by 50%	µM	75 ^8^	In-house data
K_i,u_ (CYP2E1)	Unbound inhibition constant	µM	37.5	as K_i,u_ = IC_50,u_/2
K_i,u_ (CYP3A4/5)	Unbound inhibition constant	µM	0.348 ^9^ (0.146)	In-house data
IC_50,u_ (UGT1A1)	Unbound ABL001 concentrations estimated to inhibit probe substrate reaction by 50%	µM	0.56 ^10^	In-house data
K_i,u_ (UGT1A1)	Unbound inhibition constant	µM	0.35	K_i,u_ = IC_50,u_/(1 + S/K_m_) ^11^
IC_50,u_ (UGT2B7)	Unbound ABL001 concentrations estimated to inhibit probe substrate reaction by 50%	µM	7.28 ^10^	In-house data
K_i,u_ (UGT2B7)	Unbound inhibition constant	µM	7.28	as K_i,u_ = IC_50,u_
**CYP induction**				
IndC_50_ (CYP1A2)	Induction constant	µM	0.59	In-house data
CV IndC_50_ (1A2)	% Coefficient of variation (IndC_50_)	-	30	Default
Ind_max_ (CYP1A2)	Maximum fold induction	-	4.5	In-house data
CV Ind_max_ (1A2)	% Coefficient of variation (Ind_max_)	-	30	Default
IndC_50_ (CYP3A4)	Induction constant (calibrated)	µM	2.057	Non-calibrated IndC_50_ = 2.7 µM In-house data
CV IndC_50_ (3A4)	% Coefficient of variation (IndC_50_)	-	30	Default
Ind_max_ (CYP3A4)	Maximum fold induction (calibrated)	-	1.53	Non-calibrated E_max_ = 4.4 In-house data
CV Ind_max_ (3A4)	% Coefficient of variation (Ind_max_)	-	30	
**Transporter inhibition**				
K_i_ P-gp	Inhibition constant (total)	µM	21.7	In-house data
K_i_ BCRP	Inhibition constant (total)	µM	0.088	In-house data
K_i_ OATP1B1	Inhibition constant (total)	µM	2.46	In-house data
K_i_ OATP1B3	Inhibition constant (total)	µM	1.92	In-house data
K_i_ OAT1	Inhibition constant (total)	µM	6.90	In-house data
K_i_ OAT3	Inhibition constant (total)	µM	1.01	In-house data
K_i_ OCT1	Inhibition constant (total)	µM	3.41	In-house data
K_i_ OCT2	Inhibition constant (total)	µM	8.22	In-house data
K_i_ MATE1 (MATE2K)	Inhibition constant (total)	µM	6.22 (2.36)	In-house data
K_i_ BSEP	Inhibition constant (total)	µM	No inhibition	In-house data

^1^ For information only; values are not used from the current model as the latter implements a first-order absorption model with user input values for f_a_, *k_a_*, and tlag. ^2^ The f_m_ values correspond to the final (actual) values after consideration of the contribution of BCRP-mediated biliary secretion to the overall elimination. Note that the Simcyp f_m_ output will deviate from these values, as the contribution of the efflux transporter is not included in the output pie chart. ^3^ Unbound *K*_m_ or *K*_m,u_ = *K*_m_*fu_mic_; fu_mic_ for asciminib was determined to be 0.160 for a protein concentration of 0.810 mg of protein/mL. ^4^ Unbound *K*_m_ or *K*_m,u_ (*K*_m,u_ = *K*_m_*fu_mic_); fu_mic_ for asciminib was determined to be 0.0829 for a protein concentration of 1.58 mg of protein/mL. ^5^ Unbound *K*_m_ or *K*_m,u_ (*K*_m,u_ = *K*_m_*fu_mic_); fu_mic_ for asciminib was determined to be 0.0680 for a protein concentration of 1.86 mg of protein/mL. ^6^ Unbound *K*_m_ or *K*_m,u_ (*K*_m,u_ = *K*_m_*fu_mic_); fu_mic_ for asciminib was determined to be 0.0925 for a protein concentration of 1.40 mg of protein/mL. ^7^ Unbound *K*_m_ or *K*_m,u_ (*K*_m,u_ = *K*_m_*fu_mic_); fu_mic_ for asciminib was determined to be 0.0811 for an incubation of 1.5 mg of protein/mL. ^8^ Unbound IC_50_ or IC_50,u_ (IC_50,u_ = IC_50_*fu_mic_); fu_mic_ for asciminib in CYP1A2, CYP2A6, CYP2C19, CYP2D6, and CYP2E1 was determined to be 0.208, 0.871, 0.208, 0.75, and 0.75 for 0.5, 0.025, 0.5, 0.05, and 0.05 mg of protein/mL, respectively. ^9^ Unbound fractions were 0.871, 0.750, and 0.208 for 0.025, 0.05, and 0.5 mg/mL of protein, respectively. ^10^ Unbound fractions were 0.871, 0.750, and 0.208 for 0.025, 0.05, and 0.5 mg/mL of protein, respectively. ^11^ Probe substrate (estradiol) *K*_m_ = 16.8 μM and S = 10 μM.

**Table 3:** Summary of observed and PBPK-simulated drug–drug interaction and organ impairment studies at asciminib 40 mg dose.

Trial	Drug-Drug Interaction or Organ Impairment Degree		Geometric Mean C_max_ Ratio (90% CI)			Geometric Mean AUC_inf_ Ratio (90% CI)		
	Perpetrator Dosing Regimen	Victim Dosing Regimen	Observed	Simulated	R_pred/obs_	Observed	Simulated	R_pred/obs_
[5]	Clarithromycin 500 mg BID for 8 days	Asciminib 40 mg single dose on day 5	1.19 (1.1, 1.3)	1.05 (1.04, 1.05)	0.882	1.36 (1.27, 1.46)	1.32 (1.30, 1.34)	0.971
[5]	Itraconazole capsule 200 mg QD for 8 days	Asciminib 40 mg single dose on day 5	1.04 (NA, NA)	1.05 (1.05, 1.06)	1.01	1.04 (NA, NA)	1.24 (1.22, 1.25)	1.19
[5]	Rifampicin 600 mg QD for 6 days	Asciminib 40 mg single dose on day 5	1.09 (0.996, 1.20)	0.838 (0.821, 0.855)	0.769	0.851 (0.804, 0.902)	0.566 (0.548, 0.584)	0.665
[6]	Imatinib 400 mg QD for 8 days	Asciminib 40 mg single dose on day 5	1.59 (1.45, 1.75)	1.15 (1.13, 1.17) [1.14 (1.12, 1.16)]	0.723	2.08 (1.93, 2.24)	1.99 (1.92, 2.07) [1.56 (1.52, 1.60)]	0.957
[4]	Asciminib 40 mg BID	Midazolam 4 mg on day 3	1.11 (0.957, 1.28)	1.18 (1.16, 1.19)	1.06	1.28 (1.15, 1.43)	1.23 (1.21, 1.25)	0.961
[4]	Asciminib 40 mg BID	*S*-Warfarin 2.5 mg on day 3	1.08 (1.04, 1.13)	1.03 (1.03, 1.04)	0.954	1.41 (1.37, 1.45)	1.40 (1.37, 1.42)	0.993
[4]	Asciminib 40 mg BID	Repaglinide 0.5 mg on day 3	1.14 (1.01, 1.28)	1.07 (1.07, 1.08)	0.939	1.08 (1.02, 1.14)	1.10 (1.09, 1.10)	1.02
[7]	Mild HI/HV control, 40 mg single dose		1.26 (1.05, 1.52)	0.966	0.767	1.22 (0.964, 1.54)	1.11	0.910
[7]	Moderate HI/HV control, 40 mg single dose		0.983 (0.819, 1.18)	0.908	0.924	1.03 (0.813, 1.30)	1.32	1.28
[7]	Severe HI/HV control, 40 mg single dose		1.29 (1.08, 1.55)	0.776	0.602	1.66 (1.30, 2.12)	1.28	0.771
[7]	Severe RI/HV control, 40 mg single dose		1.08 (0.719, 1.61)	1.14 [0.818] ^1^	1.06	1.56 (1.05, 2.30)	1.44 [0.970] ^1^	0.923
**GMFE**					**1.18**			**1.14**

GMFE: geometric mean fold error. ^1^ Values in brackets indicate the predicted ratios prior to the “top-down” adjustments.

**Table 4:** Summary of asciminib PBPK model applications, level of regulatory acceptance, and impacts on the drug product label.

Intended PBPK Model Application	Feeback by FDA	Rationale of FDA’s Assessment	Impact on Drug Product Label or Other
**Victim DDI**			
Extrapolation of the effects of strong CYP3A inhibitors on asciminib 80 and 200 mg dose	Not accepted yet supportive	Uncertainties in elimination pathways	Closely monitor for adverse reactions in patients treated with SCEMBLIX at 200 mg twice daily with concomitant use of strong CYP3A4 inhibitors.
Extrapolation of the effects of strong CYP3A inducers on asciminib 80 and 200 mg dose	Not accepted	Uncertainties in elimination pathways; overprediction of DDI with rifampin	Post-marketing requirement: Clinical study to assess the effect of the strong CYP3A inducer phenytoin on asciminib 200 mg single dose
Extrapolation of the effects of imatinib on asciminib 80 and 200 mg dose	Not accepted	Uncertainties in elimination pathways/IVIVE for UGTs, and BCRP not established	No mention about 80 mg QD; concomitant use of imatinib with SCEMBLIX at 200 mg twice daily has not been fully characterized.
Prediction of the effects of moderate CYP3A perpetrators on asciminib 40, 80 and 200 mg dose	Not accepted yet supportive	Uncertainties in elimination pathways	No dose adjustments or label restrictions for moderate CYP3A perpetrators.
**Perpetrator DDI**			
Extrapolation of asciminib effects at 80 mg QD and 200 mg BID on CYP3A-sensitive substrates	Accepted	PK and DDI with midazolam adequately predicted	PBPK simulation results for 80 mg QD and 200 mg BID for midazolam were reported in lieu of clinical data.
Extrapolation of asciminib effects at 80 mg QD and 200 mg BID on CYP2C9-sensitive substrates	Accepted	PK and DDI with warfarin adequately predicted	PBPK simulation results for 80 mg QD and 200 mg BID for warfarin were reported in lieu of clinical data.
Extrapolation of asciminib effects at 80 mg QD and 200 mg BID on CYP2C8-sensitive substrates	Accepted	PK and DDI with repaglinide adequately predicted	PBPK simulation results for 80 mg QD and 200 mg BID for repaglinide were reported in lieu of clinical data.
Prediction of asciminib effects at 40 mg BID, 80 mg QD, and 200 mg BID on dual CYP2C9 and CYP2C8 substrates	Accepted	PK and DDI with warfarin and repaglinide adequately predicted; additional PSA on CYP2C8 and CYP2C9 K_i,u_	PBPK simulation results for 40 mg BID, 80 mg QD, and 200 mg BID for rosiglitazone were reported in lieu of clinical data.
Prediction of asciminib effects at 40 mg BID, 80 mg QD, and 200 mg BID on CYP2C19-sensitive substrates	Accepted	Additional PSA down to twofold lower K_i,u_ indicated only a weak effect at 200 mg BID.	May reversibly inhibit CYP2C19 at concentrations reached at 200 mg twice daily dose.
Prediction of asciminib effects at 40 mg BID, 80 mg QD, and 200 mg BID on UGT1A1-sensitive substrates	Accepted	Additional PSA with a twofold lower K_i,u_, indicating potential for interaction	May reversibly inhibit UGT1A1 at plasma concentrations reached at a total daily dose of 80 mg and 200 mg twice daily.
Prediction of asciminib effects at 40 mg BID, 80 mg QD, and 200 mg BID on CYP1A2-sensitive substrates	Accepted	Additional PSA to explore the induction risk	No dose adjustments or label restrictions for CYP1A2 substrates
**Organ impairment**			
Extrapolation of the effect of hepatic impairment on asciminib 80 mg and 200 mg doses	Not accepted yet supportive	Uncertainties in elimination pathways	No dose adjustments or label restrictions for hepatic impairment
Extrapolation of the effect of renal impairment on asciminib 80 mg and 200 mg doses	Not accepted yet supportive	Uncertainties in elimination pathways	No dose adjustments or label restrictions for renal impairment

## Data Availability

The authors declare that all the data supporting the findings of this study are contained within the paper.

## Supplementary Materials

The following supporting information can be downloaded at: https://www.mdpi.com/article/10.3390/pharmaceutics17101266/s1, Table S1. Final fractional metabolic, excreted, or transported contributions of asciminib implemented in the PBPK model. Table S2. Relevant compartmental asciminib concentrations used for static DDI risk assessment after repeated doses of 40 mg BID, 80 mg QD, and 200 mg BID. Table S3. PK simulation trial design parameters in healthy volunteers and patients for the asciminib model establishment and validation. Table S4. Victim DDI simulation trial design parameters based on available clinical pharmacology studies on asciminib (at 40 mg). Table S5. Perpetrator DDI simulation trial design parameters based on available asciminib clinical pharmacology studies (at 40 mg BID). Table S6. Victim DDI simulation trial-design parameters of scenarios without available asciminib clinical pharmacology studies. Table S7. Perpetrator DDI simulation trial-design parameters of scenarios without available asciminib clinical pharmacology studies. Table S8 [7,10]. Hepatic impairment simulation trial design. Table S9 [7,11]. Renal impairment simulation trial design. Table S10. Observed and PBPK model predicted exposure changes in asciminib after administration of 40, 80, or 200 mg single or multiple doses (for 14 days) by the co-administration of CYP3A4 strong inhibitors or inducers in healthy subjects. Table S11 PBPK model predicted exposure changes in asciminib after administration of 40, 80, or 200 mg single or multiple doses (for 14 days) by co-administration of CYP3A4 moderate inhibitors or inducers in healthy subjects. Table S12. Observed and PBPK model predicted exposure changes in asciminib after administration of 40, 80, or 200 mg single or multiple doses (for 14 days) by co-administration of imatinib, a CYP3A4, UGT1A3/4, UGT2B17, and BCRP inhibitor, in healthy subjects. Table S13. Observed and PBPK model predicted exposure changes in CYP probe substrates (single dose on day 3) or multiple doses (for 14 days) by co-administration of asciminib 40 mg BID, 80 mg QD, or 200 mg BID in healthy subjects. Table S14. PBPK model predicted exposure changes in CYP, UGT, and transporter probe substrates (single dose on day 3) or multiple doses (for 14 days) by co-administration of asciminib 40 mg BID, 80 mg QD, or 200 mg BID in healthy subjects. Table S15. Summary of asciminib pharmacokinetics after oral administration of a 40, 80, or 200 mg single dose of asciminib in healthy adults and subjects with impaired hepatic function. Table S16. Summary of asciminib pharmacokinetics after oral administration of a 40, 80, or 200 mg single dose of asciminib in healthy adults and subjects with impaired renal function. Figure S1. Parameter sensitivity analysis of fu,gut and Qgut on the AUC and Cmax ratios of the midazolam–asciminib DDI. Figure S2. Parameter sensitivity analysis of CYP2C9 Ki on the warfarin AUC and Cmax ratios. Figure S3. Parameter sensitivity analysis of imatinib’s intestinal and hepatic BCRP Ki values on asciminib AUC and Cmax ratios. Figure S4. Simulated and observed plasma concentration–time profiles of asciminib (40 mg single dose) in healthy subjects and healthy subjects with mild, moderate, or severe hepatic impairment. Figure S5. Simulated and observed plasma concentration–time profiles of asciminib (40 mg single dose) in healthy subjects and healthy subjects with mild, moderate, or severe renal impairment. Figure S6. Parameter sensitivity analysis of the fraction unbound in plasma on Cmax and AUC for subjects with severe renal impairment.

## Significance Statement

This study presents the development, verification, and application of a nonlinear PBPK model for asciminib to predict untested clinical scenarios and bridge clinical pharmacology data across all the approved dosing regimens. The model supported regulatory decision making and product labeling and is estimated to have replaced approximately 10 clinical studies—exemplifying the value of PBPK modeling in streamlining drug development and regulatory submissions.

## List of Nonstandard Abbreviations

AAFE, absolute average fold error; ADME, absorption, distribution, metabolism, and excretion; AFE, average fold error; BCRP, breast-cancer-resistance protein; BID, twice daily; B/P, blood-to-plasma ratio; Caco-2, continuous heterogeneous human epithelial colorectal adenocarcinoma cell line; CI, confidence interval; CL, clearance; CL/F, apparent oral clearance; CL_int_, intrinsic clearance; CL_PD_, passive diffusion clearance in liver; CL_r_, renal clearance; C_max_, maximum concentration; CML, chronic myeloid leukemia; CML-CP, CML in the chronic phase; CP, Child–Pugh; CV, coefficient of variance; CYP cytochrome P450; DDI, drug–drug interaction; E_max_, maximum fold induction over the vehicle control; EMA, European Medicines Agency; F, oral bioavailability; f_a_, fraction of the dose absorbed; FaSSIF, fasted-state simulated intestinal fluid; FaSSGF, fasted-state simulated gastric fluid; fe, fraction excreted; F_g_, fraction of the drug that escapes intestinal first-pass metabolism; FDA, U.S. Food and Drug Administration; F_h_, fraction of the dose that escapes hepatic first-pass elimination; fm, fraction metabolized; FMI, final market image; ft, fraction transported; fu_gut_, fraction of the unbound drug in enterocytes; fu_mic_, fraction of the unbound drug in microsomes; fu_p_, fraction of the unbound drug in the plasma; GFR, glomerular filtration rate; GI, gastrointestinal; GMFE, geometric mean fold error; hADME, human ADME; HI, hepatic impairment; HLM, human liver microsomes; HV, healthy volunteer; IC_50_, concentration at half-maximal inhibition; IndC_50_ (or EC_50_), concentration at half-maximal induction; Ind_max_, maximum induction increase over the baseline; IQ, International Consortium for Innovation and Quality; J_max_, in vitro maximum rate of transporter-mediated efflux; *k*_a_, absorption rate constant; K_I_, concentration of inactivator at the half-maximal inactivation rate; *k*_inact_, rate of inactivation; *K*_i,u_, unbound reversible inhibition constant; K_p_, blood-to-tissue partition coefficient; LogP_o:w_, octanol:water partition coefficient; MDCK-LE, Madin–Darby Canine Kidney Low Efflux; MATE, multidrug and toxic compound extrusion transporter; MRP2, multidrug resistance protein 2; MW, molecular weight; NA, not applicable; NDA, new drug application; OAT, organic anion transporter; OATP, organic anion-transporting polypeptide; OCT, organic cation transporter; OI, organ impairment; PBPK, physiologically based pharmacokinetic; P_eff_, effective permeability; PerL, permeability liver model; P-gp, P-glycoprotein; PK, pharmacokinetic; pK_a_, dissociation constant; p.o., oral administration; PopPK, population pharmacokinetics; PSA, parameter sensitivity analysis; QD, once daily; Q_gut_, nominal flow through the gut; RI, renal impairment; SD, standard deviation; SF, scaling factor; TDI, time-dependent inhibition; TKI, tyrosine kinase inhibitor; tlag, lag time; T_max_, time at which C_max_ is reached; UGT, uridine 5′-diphospho (UDP)-glucuronosyltransferase; V_ss_, volume of distribution at steady state.
