# Supplementary material for: Physiologically Based Pharmacokinetic Modeling and Simulations in Lieu of Clinical Pharmacology Studies to Support the New Drug Application of Asciminib
Source: Pharmaceutics. 2025 Sep 26;17(10):1266. doi: 10.3390/pharmaceutics17101266 (PMC12567041; doi:10.3390/pharmaceutics17101266)
Supplement: Supplementary file 1 [file pharmaceutics-17-01266-s001.zip › pharmaceutics-3825015-supplementary.pdf]

Supplementary Material

Physiologically based pharmacokinetic modeling and simulations in lieu of clinical pharmacology studies to support the new drug application of asciminib

Ioannis Loisios-Konstantinidis[1*], Felix Huth[1], Matthias Hoch[1], Heidi J. Einolf[2]

[1] Pharmacokinetic Sciences, Translational Medicine, Biomedical Research, Novartis, Basel, Switzerland

[2] Pharmacokinetic Sciences, Translational Medicine, Biomedical Research, Novartis, East Hanover, New Jersey, USA

Supplementary Tables

Table S1. Final fractional metabolic, excreted, or transported contributions of asciminib implemented in the PBPK model

Pathway	*fm/fe* based on hADME	Aim for *fm*/fe/ft		PBPK output for *fm*/fe/ft	
		Simcyp output (without BCRP)[1,4]	Final *fm* (including hepatic BCRP)[3]	Simcyp output (without BCRP)	Final *fm* (including hepatic BCRP)
CYP3A4	0.351	0.509	0.351	0.525	0.36
CYP2C8	0.005	0.007	0.005	0.0076	0.0052
CYP2D6	0.002	0.003	0.002	0.004	0.0028
CYP2J2	0.0076	0.011	0.0076	0.0125	0.0086
UGT1A3	0.0705	0.048	0.033	0.0515	0.035
UGT1A4[2]	0.0705	0.048	0.033	0.0504	0.034
UGT2B7[2]	0.279	0.190	0.131	0.192	0.133
UGT2B17	0.163	0.110	0.076	0.113	0.078
Renal excretion	0.044	0.035	0.044	0.034	0.024
Hydrolysis	0.007	0.010	0.007	0.0103	0.0071
Hepatic BCRP	NA	NA	0.311	NA	0.311
Total	1.000	0.970434783	1.001	1.000	~1.00

NA: not available or not assumed.

[1]Simcyp output values are presented as arithmetic mean and correspond to the demographics with an age range of 22-55 and a female proportion of 0.286. Depending on the population and demographics, the above values might slightly vary.

[2] Sum of hepatic and renal UGT contributions.

[3] Simcyp v19.1 does not output the contribution of transporters. The aim for fm and fe in the pie chart obtained as output by Simcyp is expected to be 1.45-fold of the final fm/fe, meaning scaled by 1/(1-ft(BCRP)) (=1/0.69) to account for the contribution of BCRP (and its absence from the Simcyp output).

[4] Briefly, the contribution of BCRP was determined indirectly by comparing the simulated CL/F with (7.19 L/h) and without (4.96 L/h) the inclusion of BCRP after multiple BID dosing at the lowest clinically tested dose of 20 mg and under the assumption of no saturation at this dose level. The contribution of BCRP was deducted from the total contribution of UGTs, which gave a final total contribution of UGTs to the total asciminib elimination of about 27.3% (≃58.3-31). The relative contributions of UGT1A3/4, UGT2B7, and UGT2B17 to the total UGT contribution were 24.2%, 47.9%, and 27.9%, respectively. Therefore, the final fm values for UGT1A3/4, UGT2B7, and UGT2B17 are calculated to be about 6.6%, 13.1%, and 7.6%, respectively. On the other hand, the contributions of CYPs or renal elimination remain unchanged, with the major contribution from CYP3A4 of about 35.1% and minor contributions from the other CYPs (CYP2C8, CYP2D6, and CYP2J), hydrolysis, and renal elimination of about 1.5%, 0.7%, and 4.4%, respectively.

Table S2. Relevant compartmental asciminib concentrations used for static DDI risk assessment after repeated doses of 40 mg BID, 80 mg QD, and 200 mg BID

Dose	Parameter	Description	Assessment method	Unit	Value
**40 mg BID**	$C_{max,ss,tot}$	Total maximal steady-state plasma concentration	Measured (Study A2201)	μM	1.76
	$C_{max,ss,u}$	Unbound maximal steady-state plasma concentration	$=fu_p*C_{max,ss,tot}$	μM	0.0476
	$C_{h,in,tot}$	Total maximal hepatic inlet plasma concentration	$=C_{max,ss,tot}\cdot R_b+f_a\cdot F_g\cdot k_a\cdot D/Q_h$	μM	4.47
	$C_{h,in,u}$	Unbound maximal hepatic inlet plasma concentration at the steady state	$=fu_p\cdot C_{h,in,tot}$	μM	0.121
	C_{gut}	Maximal gut concentration	$=D/MW\cdot 4000$	μM	356
	$C_{gut,ent}$	Maximal enterocyte concentration	$=f_a\cdot k_a\cdot D/Q_{ent}$	μM	10.5
**80 mg QD**	$C_{max,ss,tot}$	Total maximal steady-state plasma concentration	Measured (Study A2201)	μM	3.96
	$C_{max,ss,u}$	Unbound maximal steady-state plasma concentration	$=fu_p\cdot C_{max,ss,tot}$	μM	0.107
	$C_{h,in,tot}$	Total maximal hepatic inlet plasma concentration	$=C_{max,ss,tot}\cdot R_b+f_a\cdot F_g\cdot k_a\cdot D/Q_h$	μM	9.38
	$C_{h,in,u}$	Unbound maximal hepatic inlet plasma concentration at the steady state	$=fu_p\cdot C_{h,in,tot}$	μM	0.253
	C_{gut}	Maximal gut concentration	$=D/MW\cdot 4000$	μM	711
	$C_{gut,ent}$	Maximal enterocyte concentration	$=f_a\cdot k_a\cdot D/Q_{ent}$	μM	20.9
**200 mg BID**	$C_{max,ss,tot}$	Total maximal steady-state plasma concentration	Measured (Study A2201)	μM	12.5
	$C_{max,ss,u}$	Unbound maximal steady-state plasma concentration	$=fu_p\cdot C_{max,ss,tot}$	μM	0.339
	$C_{h,in,tot}$	Total maximal hepatic inlet plasma concentration	$=C_{max,ss,tot}\cdot R_b+f_a\cdot F_g\cdot k_a\cdot D/Q_h$	μM	26.1
	$C_{h,in,u}$	Unbound maximal hepatic inlet plasma concentration at the steady state	$=fu_p\cdot C_{h,in,tot}$	μM	0.704
	C_{gut}	Maximal gut concentration	$=D/MW\cdot 4000$	μM	1778
	$C_{gut,ent}$	Maximal enterocyte concentration	$=f_a\cdot k_a\cdot D/Q_{ent}$	μM	52.4

fu_p: unbound fraction in plasma; k_a: first-order absorption rate constant, MW: molecular weight; Q_{ent}: enterocyte blood flow; Q_h: hepatic blood flow; R_b: blood cell-to-plasma concentration ratio.
Parameters used: fu_p (asciminib)=0.027 and R_b=0.80 [1], fa=1, Fg=1, k_a=0.0353 min-1, D=449.85 mg, Q_h=1.45 L/min [2], MW (asciminib)=449.85 g/mol, and Q_{ent}=0.3 L/min [3].

Table S3. PK simulation trial design parameters in healthy volunteers and patients for the asciminib model establishment and validation.

Description[1]	Population model	Administration and dose (mg)	Start–end days (duration)	No. of doses	PK-sampling duration (h)	Age range (y)	Proportion of females
**Healthy volunteers**							
[4]	NEur. Caucasian	40	1-5 (4)	1	96	27-55	0.167
[4]	NEur. Caucasian	40	1-5 (4)	1	96	22-55	0.286
[4]	NEur. Caucasian	40	1-5 (4)	1	96	23-54	0.20
[5]	NEur. Caucasian	40	1-4 (3)	1	72	47-61	0.25
[5]	NEur. Caucasian	40	1-4 (3)	1	72	49-71	0.333
**Cancer patients**							
First in human trial [1,6]							
20 mg BID	Cancer	20 mg BID	1-28 (27.5)	55	660	27-74	0.5714
40 mg BID	Cancer	40 mg BID	1-28 (27.5)	55	660	27-75	0.457
80 mg BID	Cancer	80 mg BID	1-28 (27.5)	55	660	23-66	0.333
160 mg BID	Cancer	160 mg BID	1-28 (27.5)	55	660	27-73	0.4545
200 mg BID	Cancer	200 mg BID	1-28 (27.5)	55	660	25-86	0.274
80 mg QD	Cancer	80 mg QD	1-29 (28)	28	672	30-86	0.555
120 mg QD	Cancer	120 mg QD	1-29 (28)	28	672	38-88	0.4545
200 mg QD	Cancer	200 mg QD	1-29 (28)	28	672	27-79	0.5
Phase III trial [7]							
40 mg BID	Cancer	40 mg BID	1-29 (28)	56	672	24-83	0.4807

[1]All the simulations were performed as 10 trials of 10 subjects (10 trials x 10 subjects, default scenario).

Table S4. Victim DDI simulation trial design parameters based on available clinical pharmacology studies on asciminib (at 40 mg).

Description	DDI simulation				
Reference		[4]			[8]
Compound	**Asciminib**	**Clarithromycin**[1]	**Itraconazole**[1]	**Rifampicin**[1]	**Imatinib**[4]
Victim or perpetrator	Victim	Perpetrator	Perpetrator	Perpetrator	Perpetrator
Dose (mg, p.o.)	40, 80, 200	500	200	600	400
Administration	Single	Multiple (BID)	Multiple (QD)	Multiple (QD)	Multiple (QD)
Start on day	5	1	1	1	1
Number of doses	1	16	8	6	8
Trial duration (days)		8	8	6	8
Dose (mg, p.o.)	40, 80, 200	500	200	600	400
Administration	BID, QD, BID	Multiple (BID)	Multiple (QD)	Multiple (QD)	Multiple (QD)
Start on day	1	1	1	1	1
Number of doses	28, 14, 28[5]	28	28	14	14
Trial duration (days)	14	14	28	14	14
Population	Simcyp North European Caucasian (Sim-NEurCaucasian)				
Trial size[3]	10 x 10				
Age range (years)		22-55	23-54	27-55	21-54
Female proportion		0.286	0.20	0.1666	0.0416

[1]Simcyp library compound files used.
[2]Reference [9].
[3]A total of 10 trials of 10 subjects (10 x 10), default scenario.
[4]The imatinib inhibitor file was developed using the input parameters of the published imatinib PBPK model by [10] and additional in-house inhibition parameters (Ki) for UGT1A3, UGT1A4, and UGT2B17.
[5]Numbers of doses of asciminib at 40 mg BID, 80 mg QD, and 200 mg BID for itraconazole DDI simulations were 56, 28, and 56, respectively.

Table S5. Perpetrator DDI simulation trial design parameters based on available asciminib clinical pharmacology studies (at 40 mg BID)

**Description**	**DDI simulation**			
Reference		[5]		
Compound	**Asciminib**	**Midazolam**[1] **(Part 1)**	***S*-Warfarin**[1] **(Part 1)**	**Repaglinide**[1] **(Part 2)**
Victim or perpetrator	Perpetrator	Victim	Victim	Victim
**Single dose**				
Dose (mg, p.o.)	40, 80, 200	4	2.5	0.5
Administration	BID, QD, BID	Single	Single	Single
Start day	1	3	3	3
Number of doses (Part 1/ Part 2)	10, 5, 10 / 6, 3, 6	1	1	1
Trial duration (days)		5	8	3
**Multiple dosing**				
Dose (mg, p.o.)	40, 80, 200	4	2.5	0.5
Administration	BID, QD, BID	Multiple (QD)	Multiple (QD)	Multiple (QD)
Start day	1	1	1	1
Number of doses (with Midazolam, Repaglinide/ *S*-Warfarin)	28, 14, 28 / 70, 35, 126	14	35,35,63	14
Trial duration (days)		14	35,35,63	14
Population	Simcyp North European Caucasian (NEurCaucasian)			
Trial size[2]	10 x 10			
Female proportion		0	0	0.016
Age range (years)		23-54	23-54	26-55

[1]Simcyp library compound files used.
[2]A total of 10 trials of 10 subjects (10 x 10), default scenario.

Table S6. Victim DDI simulation trial-design parameters of scenarios without available asciminib clinical pharmacology studies.

Description	DDI simulation			
Compound	**Asciminib**	**Efavirenz[1]**	**Fluconazole[1]**	**Erythromycin[1]**
Victim or perpetrator	Victim	Perpetrator	Perpetrator	Perpetrator
**Single dose**				
Dose (mg, p.o.)	40, 80, 200	600	200	500
Administration	Single	Multiple (QD)	Multiple (QD)	Multiple (QID)
Start day	5	1	1	1
Number of doses (Part 1/ Part 2)	1	8	8	32
Trial duration (days)		8	8	8
**Multiple dosing**				
Dose (mg, p.o.)	40, 80, 200	600	200	500
Administration	BID, QD, BID	Multiple (QD)	Multiple (QD)	Multiple (QID)
Start day	1	1	1	1
Number of doses	28, 14, 28	14	14	56
Trial duration (days)	14	14	14	14
Population	Simcyp North European Caucasian (NEurCaucasian)			
Trial size[2]	10 x 10			
Female proportion	0.5			
Age range (years)	20-55			

[1]Simcyp library compound files used.
[2]A total of 10 trials of 10 subjects (10 x 10), default scenario.

Table S7. Perpetrator DDI simulation trial-design parameters of scenarios without available asciminib clinical pharmacology studies.

Description	DDI simulation			
Compound	**Asciminib**	**Caffeine**[1]	**Raltegravir**[1]	**Omeprazole**[1]
Victim or perpetrator	Perpetrator	Victim	Victim	Victim
**Single dose**				
Dose (mg, p.o.)	40, 80, 200	150	400	20
Administration	BID, QD, BID	Single	Single	Single
Start day	1	3	3	3
Number of doses	8, 4, 8	1	1	1
Trial duration (days)		5	5 [3]	5
**Multiple dosing**				
Dose (mg, p.o.)	40, 80, 200	150	400	20
Administration	BID, QD, BID	Multiple (QD)	Multiple (BID)	Multiple (BID)
Start day	1	1	1	1
Number of doses	28, 14, 28	14	28	28
Trial duration (days)		14	14 [3]	14
Population	Simcyp North European Caucasian (NEurCaucasian)			
Trial size[2]	10 x 10			
Female proportion	0.5			
Age range (years)	20-55			

[1]Simcyp library compound files used.
[2]A total of 10 trials of 10 subjects (10 x 10), default scenario.
[3]Victim drug was administered on day 3, one hour after the administration of the perpetrator, as this was observed to maximize the interaction effect (data not shown). In these cases, the number of administered perpetrator doses is increased by one.

Table S8. Hepatic impairment simulation trial design.

**Description**	**Hepatic Impairment**			
Reference	[5]			
Compound	Asciminib			
Level of hepatic impairment	**Healthy (control)**	**Mild**	**Moderate**	**Severe**
Dose (mg, p.o.)	40, 80, 200	40, 80, 200	40, 80, 200	40, 80, 200
Administration	Single	Single	Single	Single
Start day	1	1	1	1
Number of doses	1	1	1	1
Trial duration (days)	3	3	3	3
Trial size[1]	10 x 10			
Population	NEurCaucasian_Cirrhosis_control[2]	Sim-CirrhosisCP-A[3]	Sim-CirrhosisCP-B[3]	Sim-CirrhosisCP-C[3]/Cirrhosis CP-C_modified UGT1A4_2B7[4]
Female proportion[5]	0.25	0.125	0.25	0.156
Age range (years)[5]	47-61	48-68	48-65	45-65

[1] A total of 10 trials of 10 subjects (10 x 10), default scenario.
[2] NEurCaucasian matched for the demographics of the hepatic impairment population.
[3] Simcyp library population files.
[4] Modified Sim-CirrhosisCP-C population to account for the reduced activities of UGT1A4 and UGT2B7 in CP-C patients according to Prasad et al. [11].
[5] Actual female proportion and age range of the respective clinical trial cohort.

Table S9. Renal impairment simulation trial design.

**Description**	**Renal Impairment**			
Reference	[5]			
Compound	Asciminib			
Level of renal impairment	**Healthy (control)**	**Mild**	**Moderate**	**Severe**
Dose (mg, p.o.)	40, 80, 200	40, 80, 200	40, 80, 200	40, 80, 200
Administration	Single	Single	Single	Single
Start day	1	1	1	1
Number of doses	1	1	1	1
Trial duration (days)	3	3	3	3
Trial size[1]	10 x 10			
Population	NEurCaucasian_Renal impair_control [2]	Mild RI_RenalGFR_60-90[6]	Sim-RenalGFR_30-60[3]	Sim-RenalGFR_less_30[3]
Female proportion[5]	0.333	0.375[4]	0.375[4]	0.375
Age range (years)[5]	49-71	46-66[4]	46-66[4]	46-66

[1]A total of 10 trials of 10 subjects (10 x 10), default scenario.
[2]NEurCaucasian matched for the demographics of the renal impairment population.
[3]Simcyp library population files.
[4]Proportion of females and age range assumed to be the same as those in severe renal impairment.
[5]Actual female proportion and age range of the respective clinical trial cohort.
[6]Virtual population for mild renal impairment (GFR=60-89 mL/min/1.73 m^2) is currently not available in the Simcyp Simulator v19.1. By adjusting the ReanlGFR_30-60 virtual population model, a mild renal impairment population was created and used. The GFR was set at 60-89 mL/min/1.73 m^2 and the serum creatinine level at 93-102 µmol/L, and the liver CYP3A4 abundance was equal to the abundance of healthy volunteers (liver CYP3A4 abundance = 137 pmol/mg) according to Heimbach et al. [12].

Table S10. Observed and PBPK model predicted exposure changes in asciminib after administration of 40, 80, or 200 mg single or multiple doses (for 14 days) by the co-administration of CYP3A4 strong inhibitors or inducers in healthy subjects.

Perpetrator (evaluated pathway)	Victim	Geometric Mean C_{max} ratio (90% CI)				Geometric Mean AUC_{inf} (single dose) or AUC_{tau} ratio (multiple doses) (90% CI)			
	Asciminib dose (mg)	**40**		**80**	**200**	**40**		**80**	**200**
	**Dose regimen of asciminib**[1]	Observed[2]	Simulated	Simulated	Simulated	Observed[2]	Simulated	Simulated	Simulated
Clarithromycin (CYP3A4 inhibition)	Single dose on Day 5	**1.19 (1.1, 1.3)**	1.05 (1.04, 1.05)	1.05 (1.04, 1.05)	1.05 (1.04, 1.05)	**1.36 (1.27, 1.46)**	1.32 (1.30, 1.34)	1.40 (1.37, 1.42)	1.50 (1.46, 1.53)
%PE[3]			*%PE=-11.8*				*%PE=-2.94*		
Guest criteria limits (no variability)		1.026 - 1.38				1.075 - 1.72			
Guest criteria limits (20% variability)		0.87 - 1.63				0.94 – 1.97			
	Multiple dosing[2] (for 14 days)	NA	1.34 (1.31, 1.37)	1.20 (1.19, 1.22)	1.49 (1.46, 1.53)	NA	1.57 (1.53, 1.61)	1.56 (1.51, 1.60)	1.77 (1.71, 1.82)
Itraconazole capsule (CYP3A4 inhibition)	Single dose on Day 5	**1.04 (NA, NA)**	1.05 (1.05, 1.06)	1.05 (1.05, 1.06)	1.05 (1.04, 1.05)	**1.04 (NA, NA)**	1.24 (1.22, 1.25)	1.28 (1.26, 1.30)	1.34 (1.32, 1.36)
%PE[3]			*%PE=0.96*				*%PE=19.2*		
Guest criteria limits (no variability)		1.00 – 1.08				1.00 – 1.08			
Guest criteria limits (20% variability)		0.81 – 1.33				0.81 – 1.33			
	Multiple dosing (for 14 days)	NA	1.24 (1.22, 1.26)	1.20 (1.18, 1.22)	1.36 (1.33, 1.39)	NA	1.37 (1.34, 1.40)	1.48 (1.45, 1.52)	1.52 (1.48, 1.57)
Rifampicin (CYP3A4 induction)	Single dose on Day 5	**1.09 (0.996, 1.20)**	0.838 (0.821, 0.855)	0.837 (0.821, 0.855)	0.838 (0.821, 0.855)	**0.851 (0.804, 0.902)**	0.566 (0.548, 0.584)	0.531 (0.514, 0.549)	0.492 (0.476, 0.510)
%PE[3]			*%PE=-23.1*				*%PE=-33.5*		
Guest criteria limits (no variability)		1.00 – 1.18				0.74 – 0.98			

Guest criteria limits (20% variability)		0.83 – 1.43				0.62 – 1.16			
	Multiple dosing (for 14 days)	NA	0.575 (0.556, 0.594)	0.765 (0.749, 0.783)	0.531 (0.513, 0.550)	NA	0.413 (0.396, 0.431)	0.480 (0.463, 0.497)	0.366 (0.349, 0.383)

NA, not available.

[1]The number of subjects, age range, and proportion of females in the study by Hoch et al. were used [4]. The simulated trials consisted of 10 trials of 10 subjects (n=100), with the age range and proportion of females matching the actual demographics of the respective clinical studies. The population model used was the North European Caucasian (NEC) model. Asciminib was administered as a single 40, 80, or 200 mg oral dose on Day 5, whereas the inhibitor was dosed from Day 1 until Day 9 and the inducer from Day 1 until Day 7. Asciminib was administered as multiple (Days 1-15) 40 or 200 mg BID and 80 QD doses for 14 days, with clarithromycin or rifampicin being administered for 14 days as well. For the simulation of the itraconazole DDI, the dosing was extended for both asciminib and itraconazole to 28 days.

[2]Observed values are reported as the adjusted geometric mean values.

[3]PE%, calculated prediction error (%) = [(predicted value – observed value)/observed value] x 100.

[4]The predicted geometric mean AUC and C_{max} ratios were within 30% of the actual values and within the Guest criteria.

Table S11 PBPK model predicted exposure changes in asciminib after administration of 40, 80, or 200 mg single or multiple doses (for 14 days) by co-administration of CYP3A4 moderate inhibitors or inducers in healthy subjects.

Perpetrator (evaluated pathway)	Victim	Geometric Mean C_{max} ratio (90% CI)			Geometric Mean AUC_{inf} (single dose) or AUC_{tau} ratio (multiple doses) (90% CI)		
	Asciminib dose (mg)	**40**	**80**	**200**	**40**	**80**	**200**
	**Dose regimen of asciminib[1]**	Simulated	Simulated	Simulated	Simulated	Simulated	Simulated
Erythromycin (CYP3A4 inhibition)	Single dose on Day 5	1.05 (1.04, 1.05)	1.04 (1.04, 1.05)	1.04 (1.04, 1.05)	1.34 (1.31, 1.36)	1.42 (1.39, 1.45)	1.53 (1.50, 1.56)
	Multiple dosing[2] (for 14 days)	1.36 (1.33, 1.39)	1.22 (1.20, 1.24)	1.50 (1.46, 1.53)	1.60 (1.56, 1.65)	1.59 (1.55, 1.63)	1.77 (1.72, 1.83)
Fluconazole (CYP3A4 and UGT2B7 inhibition)	Single dose on Day 5	1.03 (1.02, 1.03)	1.03 (1.02, 1.03)	1.02 (1.02, 1.03)	1.18 (1.17, 1.19)	1.21 (1.20, 1.23)	1.26 (1.24, 1.27)
	Multiple dosing (for 14 days)	1.24 (1.23, 1.26)	1.15 (1.13, 1.16)	1.32 (1.30, 1.34)	1.40 (1.38, 1.42)	1.41 (1.39, 1.43)	1.49 (1.47, 1.52)
Fluconazole (CYP3A4 inhibition only)	Single dose on Day 5	1.02 (1.02, 1.03)	1.02 (1.02, 1.02)	1.02 (1.02, 1.02)	1.14 (1.13, 1.15)	1.16 (1.15, 1.17)	1.20 (1.18, 1.21)
	Multiple dosing (for 14 days)	1.21 (1.19, 1.22)	1.12 (1.12, 1.13)	1.27 (1.26, 1.29)	1.34 (1.32, 1.36)	1.35 (1.33, 1.37)	1.42 (1.40, 1.44)
Efavirenz (CYP3A4 induction)	Single dose on Day 5	0.978 (0.975, 0.982)	0.979 (0.976, 0.982)	0.979 (0.977, 0.982)	0.783 (0.766, 0.799)	0.752 (0.734, 0.770)	0.718 (0.699, 0.736)
	Multiple dosing (for 14 days)	0.821 (0.807, 0.835)	0.911 (0.901, 0.920)	0.772 (0.757, 0.787)	0.674 (0.655, 0.693)	0.676 (0.658, 0.694)	0.620 (0.601, 0.640)

[1]The simulated trials consisted of 10 trials of 10 subjects (n=100), with an age range of 20-55 years and a proportion of females of 0.5. The population model used was the North European Caucasian (NEC) population.

[2]Asciminib was administered as a single 40, 80, or 200 mg oral dose on Day 5, whereas the perpetrator was dosed from Day 1 until Day 9. Asciminib was administered as multiple (Days 1-15) 40 or 200 mg BID and 80 QD doses for 14 days, with the perpetrator being administered for 14 days as well.

Table S12. Observed and PBPK model predicted exposure changes in asciminib after administration of 40, 80, or 200 mg single or multiple doses (for 14 days) by co-administration of imatinib, a CYP3A4, UGT1A3/4, UGT2B17, and BCRP inhibitor, in healthy subjects.

**Perpetrator (evaluated pathway)**	**Perpetrator**	**Geometric Mean C_{max} ratio (90%CI)**				**Geometric Mean AUC_{inf} (single dose) or AUC_{tau} ratio (multiple doses) (90% CI)**			
	**Asciminib dose (mg)**	**40**		**80**	**200**	**40**		**80**	**200**
	**Dose regimen of asciminib [1]**	Observed [2]	Simulated	Simulated	Simulated	Observed	Simulated	Simulated	Simulated
Imatinib [3] (CYP3A4, UGT1A3/4, UGT2B17 and BCRP inhibition)	Single dose on Day 5	**1.59 (1.45, 1.75)**	1.15 (1.13, 1.17)[4]	1.17 (1.15, 1.19)	1.13 (1.12, 1.15)	**2.08 (1.93, 2.24)**	1.99 (1.92, 2.07)	1.82 (1.76, 1.89)	1.91 (1.85, 1.98)
%PE[5]			*%PE=-27.7*				*%PE=-4.33*		
	Multiple dosing [6] (for 14 days)	NA	1.72 (1.66, 1.78)	1.46 (1.42, 1.50)	1.81 (1.75, 1.87)	NA	2.09 (2.01, 2.17)	2.08 (2.00, 2.16)	2.14 (2.06, 2.22)
Imatinib (BCRP inhibition only)	Single dose on Day 5	NA	[1.01 (1.01, 1.01)]	[1.01 (1.01, 1.01)]	1.00 (1.00, 1.00)	NA	[1.20 (1.18, 1.21)]	[1.13 (1.12, 1.14)]	1.06 (1.06, 1.07)
	Multiple dosing (for 14 days)	NA	1.08 (1.07, 1.09)	1.04 (1.03, 1.04)	1.01 (1.01, 1.01)	NA	1.12 (1.11, 1.14)	1.11 (1.10, 1.12)	1.01 (1.01, 1.01)
Imatinib (CYP3A4 inhibition only)	Single dose on Day 5	NA	1.04 (1.03, 1.04)	1.04 (1.03, 1.04)	1.04 (1.03, 1.04)	NA	1.26 (1.24, 1.27)	1.32 (1.30, 1.35)	1.42 (1.39, 1.45)
	Multiple dosing (for 14 days)	NA	1.28 (1.25, 1.30)	1.16 (1.14, 1.18)	1.46 (1.43, 1.50)	NA	1.44 (1.40, 1.48)	1.43 (1.40, 1.47)	1.67 (1.62, 1.72)

Perpetrator (evaluated pathway)	Perpetrator	Geometric Mean C_{max} ratio (90%CI)				Geometric Mean AUC_{inf} (single dose) or AUC_{tau} ratio (multiple doses) (90% CI)			
	**Asciminib dose (mg)**	**40**		**80**	**200**	**40**		**80**	**200**
Imatinib (UGT1A3/4, UGT2B17 inhibition only)	Single dose on Day 5	NA	1.09 (1.08, 1.11)	1.09 (1.07, 1.10)	1.08 (1.07, 1.10)	NA	1.14 (1.12, 1.16)	1.15 (1.12, 1.17)	1.15 (1.13, 1.17)
	Multiple dosing (for 14 days)	NA	1.11 (1.09, 1.13)	1.11 (1.09, 1.12)	1.12 (1.10, 1.14)	NA	1.14 (1.12, 1.16)	1.16 (1.13, 1.18)	1.14 (1.12, 1.17)

NA, not available.

[1]The number of subjects, age range, and proportion of females in the study reported by [8] were used. The simulated trials consisted of 10 trials of 10 subjects (n=100), with the age range and proportion of females matching the actual demographics of the respective clinical studies. The population model used was the North European Caucasian (NEC) model. Asciminib and Imatinib were administered with a low-fat meal and a glass of water.

[2]Observed values are reported as the adjusted geometric mean values.

[3]The Imatinib inhibitor file was developed using the input parameters of the published Imatinib PBPK model by [10] and additional in-house inhibition parameters (K_i) for UGT1A3, UGT1A4. and UGT2B7.

[4]All the values correspond to the predictions using the adjusted Imatinib in vitro IC50 (BCRP) value (intestinal and hepatic) of 0.094 µM, which was optimized based on PSA to the Imatinib clinical DDI and supportive literature. The Imatinib in vitro IC50 (BCRP) value initially reported by [10] was 0.94 µM.

[5]PE%, calculated prediction error (%) = [(predicted value – observed value)/observed value] x 100.

[6]Asciminib was administered as a single 40, 80, or 200 mg oral dose on Day 5, whereas the perpetrator was dosed from Day 1 until Day 9. Asciminib was administered as multiple (Days 1-15) 40 or 200 mg BID and 80 QD doses for 14 days, with the perpetrator being administered for 14 days as well.

Table S13. Observed and PBPK model predicted exposure changes in CYP probe substrates (single dose on day 3) or multiple doses (for 14 days) by co-administration of asciminib 40 mg BID, 80 mg QD, or 200 mg BID in healthy subjects.

Probe Substrate (Evaluated Pathway)	Perpetrator	Geometric Mean C_{max} ratio (90%CI)				Geometric Mean AUC_{inf} (single dose) or AUC_{tau} ratio (multiple dose) (90% CI)			
	**Asciminib dose (mg)**	**40**		**80**	**200**	**40**		**80**	**200**
	**Dose regimen of victim[1]**	Observed[2]	Simulated	Simulated	Simulated	Observed	Simulated	Simulated	Simulated
Midazolam (CYP3A4 inhibition)	Single dose on day 3[3]	**1.11 (0.957, 1.28)**	1.18 (1.16, 1.19) [1.19 (1.17, 1.21)][4]	1.17 (1.15, 1.18)	1.58 (1.53, 1.62)	**1.28 (1.15, 1.43)**	1.23 (1.21, 1.25) [1.25 (1.23, 1.27)]	1.24 (1.22, 1.26)	1.88 (1.82, 1.95)
%PE [5]			*%PE= 6.31* *[%PE= 7.21]*				*%PE=- 3.91* *[%PE= -2.34]*		
	Multiple dosing[6] (for 14 days)	NA	1.17 (1.16, 1.19)	1.16 (1.15, 1.18)	1.58 (1.53, 1.63)	NA	1.23 (1.21, 1.25)	1.24 (1.22, 1.26)	1.86 (1.80, 1.94)
S-Warfarin (CYP2C9 inhibition)	Single dose on Day 3	**1.08 (1.04, 1.13)**	1.03 (1.03, 1.04)	1.04 (1.04, 1.04)	1.07 (1.07, 1.08)	**1.41 (1.37, 1.45)**	1.40 (1.38, 1.43)	1.52 (1.49, 1.55)	4.14 (3.94, 4.35)
%PE			*%PE=- 4.63*				*%PE=- 0.71*		
	Multiple dosing (for 14 days)[7]	NA	1.39 (1.36, 1.43)	1.37 (1.34, 1.41)	3.38 (3.19, 3.57)	NA	1.57 (1.53, 1.60)	1.57 (1.53, 1.60)	4.41 (4.20, 4.63)

Probe Substrate (Evaluated Pathway)	Perpetrator	Geometric Mean C_{max} ratio (90%CI)				Geometric Mean AUC_{inf} (single dose) or AUC_{tau} ratio (multiple dose) (90% CI)			
	**Asciminib dose (mg)**	**40**		**80**	**200**	**40**		**80**	**200**
	**Dose regimen of victim[1]**	Observed [2]	Simulated	Simulated	Simulated	Observed	Simulated	Simulated	Simulated
Repaglinide (CYP2C8, CYP3A4, OATP1B1 inhibition)	Single dose on Day 3	**1.14 (1.01, 1.28)**	1.07 (1.07, 1.08)	1.08 (1.07, 1.08)	1.25 (1.24, 1.26)	**1.08 (1.02, 1.14)**	1.10 (1.09, 1.10)	1.12 (1.11, 1.13)	1.42 (1.39, 1.44)
%PE			*%PE=- 6.14*				*%PE= 1.85*		
Repaglinide (CYP2C8, CYP3A4, OATP1B1 inhibition)	Multiple dosing (for 14 days)[8]	NA	1.07 (1.07, 1.08)	1.08 (1.07, 1.08)	1.25 (1.24, 1.27)	NA	1.10 (1.09, 1.10)	1.12 (1.11, 1.13)	1.42 (1.39, 1.45)
Repaglinide (CYP2C8 inhibition)	Single dose on Day 3	NA	1.01 (1.01, 1.01)	1.00 (1.00, 1.01)	1.05 (1.05, 1.05)	NA	1.02 (1.02, 1.02)	1.02 (1.02, 1.02)	1.10 (1.09, 1.11)
Repaglinide (CYP2C8 inhibition)	Multiple dosing (for 14 days)	NA	1.01 (1.01, 1.01)	1.00 (1.00, 1.01)	1.05 (1.05, 1.06)	NA	1.02 (1.02, 1.02)	1.02 (1.02, 1.02)	1.11 (1.10, 1.11)
Repaglinide (OATP1B1 inhibition)	Single dose on Day 3	NA	1.01 (1.01, 1.01)	1.02 (1.02, 1.02)	1.06 (1.06, 1.07)	NA	1.02 (1.02, 1.02)	1.03 (1.03, 1.03)	1.09 (1.09, 1.10)

Probe Substrate (Evaluated Pathway)	Perpetrator	Geometric Mean C_{max} ratio (90%CI)				Geometric Mean AUC_{inf} (single dose) or AUC_{tau} ratio (multiple dose) (90% CI)			
	**Asciminib dose (mg)**	**40**		**80**	**200**	**40**		**80**	**200**
	**Dose regimen of victim[1]**	Observed[2]	Simulated	Simulated	Simulated	Observed	Simulated	Simulated	Simulated
Repaglinide (OATP1B1 inhibition)	Multiple dosing (for 14 days)	NA	1.01 (1.01, 1.01)	1.02 (1.02, 1.02)	1.07 (1.06, 1.07)	NA	1.02 (1.02, 1.02)	1.03 (1.02, 1.03)	1.10 (1.09, 1.10)
Repaglinide (CYP3A4 inhibition+ induction)	Single dose on Day 3	NA	1.05 (1.05, 1.06)	1.05 (1.05, 1.06)	1.12 (1.11, 1.12)	NA	1.06 (1.06, 1.07)	1.07 (1.07, 1.08)	1.16 (1.15, 1.17)
Repaglinide (CYP3A4 inhibition+ induction)	Multiple dosing (for 14 days)	NA	1.05 (1.05, 1.06)	1.05 (1.05, 1.06)	1.12 (1.11, 1.12)	NA	1.06 (1.06, 1.07)	1.07 (1.06, 1.08)	1.16 (1.14, 1.17)
Repaglinide (CYP3A4 inhibition)	Single dose on Day 3	NA	1.05 (1.05, 1.06)	1.06 (1.05, 1.06)	1.12 (1.11, 1.13)	NA	1.06 (1.06, 1.07)	1.07 (1.07, 1.08)	1.16 (1.15, 1.18)
Repaglinide (CYP3A4 inhibition)	Multiple dosing (for 14 days)	NA	1.05 (1.05, 1.06)	1.06 (1.05, 1.06)	1.12 (1.11, 1.13)	NA	1.06 (1.06, 1.07)	1.07 (1.07, 1.08)	1.17 (1.15, 1.18)

NA, not available.

[1]The number of subjects, age range, and proportion of females in the study by Hoch et al. were used [13]. The simulated trials consisted of 10 trials of 10 subjects (n=100), with the age range and proportion of females matching the actual demographics of the respective clinical studies. The population model used was the North European Caucasian (NEC) model.

[2]Observed values are reported as the adjusted geometric mean values.

[3]In the single-dose DDI, the substrate was administered on Day 3. For repaglinide, the ratios are based on the geometric mean AUC_{last} value, as AUCinf was not outputted from the model.

[4]Values within brackets correspond to the predictions with CYP3A4/5 inhibition only (*i.e.,* without CYP3A4 induction). As the CYP3A4 induction potential of asciminib is very low, it was not further considered as relevant.

[5]PE%, calculated prediction error (%) = [(predicted value – observed value)/observed value] x 100.

[6]Midazolam was administered for 14 days (d1-d15), whereas asciminib was administered as 40 or 200 mg BID and 80 mg QD doses for 14 days (d1-15) as well.

[7]*S*-Warfarin and asciminib were administered for 35 days (d1-d36) at 40 mg BID and 80 mg QD or for 63 days (d1-d64) at 200 mg BID.

[8]Repaglinide was administered for 14 days (d1-d15) and asciminib was also administered for 14 days (d1-d15) at 40 or 200 mg BID and 80 mg QD doses.

Table S14. PBPK model predicted exposure changes in CYP, UGT, and transporter probe substrates (single dose on day 3) or multiple doses (for 14 days) by co-administration of asciminib 40 mg BID, 80 mg QD, or 200 mg BID in healthy subjects.

Probe Substrate (evaluated pathway)	Perpetrator	Geometric Mean C_{max} ratio (90%CI)			Geometric Mean AUC_{inf} (single dose) or AUC_{tau} ratio (multiple doses) (90% CI)		
	**Asciminib dose (mg)**	**40**	**80**	**200**	**40**	**80**	**200**
	**Dose regimen of victim drug [1]**	Simulated	Simulated	Simulated	Simulated	Simulated	Simulated
Caffeine (CYP1A2 inhibition and induction)	Single dose on Day 3	0.989 (0.988, 0.991)	0.989 (0.988, 0.990)	0.949 (0.944, 0.954)	0.945 (0.939, 0.950)	0.941 (0.934, 0.947)	0.777 (0.760, 0.794)
	Multiple dosing [2] (for 14 days)	0.964 (0.961, 0.968)	0.965 (0.961, 0.968)	0.863 (0.853, 0.873)	0.901 (0.891, 0.910)	0.901 (0.892, 0.910)	0.645 (0.625, 0.665)
Caffeine (CYP1A2 induction only)	Single dose on Day 3	0.989 (0.988, 0.990)	0.989 (0.987, 0.990)	0.947 (0.941, 0.952)	0.942 (0.937, 0.948)	0.938 (0.932, 0.944)	0.766 (0.749, 0.783)
	Multiple dosing (for 14 days)	0.964 (0.960, 0.968)	0.964 (0.960, 0.968)	0.859 (0.849, 0.869)	0.899 (0.889, 0.908)	0.898 (0.889, 0.908)	0.635 (0.616, 0.655)
Caffeine (CYP1A2 inhibition only)	Single dose on Day 3	1.00 (1.00, 1.00)	1.00 (1.00, 1.00)	1.00 (1.00, 1.00)	1.00 (1.00, 1.00)	1.00 (1.00, 1.00)	1.01 (1.01, 1.02)
	Multiple dosing (for 14 days)	1.00 (1.00, 1.00)	1.00 (1.00, 1.00)	1.00 (1.00, 1.01)	1.00 (1.00, 1.00)	1.00 (1.00, 1.00)	1.02 (1.01, 1.02)
Omeprazole (CYP2C19 inhibition)	Single dose on Day 3	1.03 (1.03, 1.03)	1.04 (1.03, 1.04)	1.12 (1.11, 1.13)	1.04 (1.04, 1.05)	1.06 (1.06, 1.06)	1.22 (1.20, 1.23)
	Multiple dosing (for 14 days)	1.04 (1.04, 1.05)	1.04 (1.03, 1.04)	1.23 (1.21, 1.24)	1.07 (1.07, 1.08)	1.06 (1.05, 1.06)	1.41 (1.38, 1.45)
Raltegravir (UGT1A1 inhibition)	Single dose on Day 3 [3]	1.15 (1.14, 1.17)	1.21 (1.19, 1.22)	1.51 (1.48, 1.54)	1.16 (1.14, 1.17)	1.22 (1.20, 1.24)	1.61 (1.57, 1.64)[4]
	Multiple dosing (for 14 days) [3]	1.16 (1.15, 1.17)	1.09 (1.08, 1.10)	1.54 (1.51, 1.57)	1.16 (1.15, 1.17)	1.09 (1.08, 1.10)	1.62 (1.58, 1.66)

Probe Substrate (evaluated pathway)	Perpetrator	Geometric Mean C_{max} ratio (90%CI)			Geometric Mean AUC_{inf} (single dose) or AUC_{tau} ratio (multiple doses) (90% CI)		
	**Asciminib dose (mg)**	**40**	**80**	**200**	**40**	**80**	**200**

[1] The simulated trials consisted of 10 trials of 10 subjects (n=100), with an age range of 20-55 years and a proportion of females of 0.5. The population model used was the North European Caucasian (NEC) population.
[2] The probe substrate was administered for 14 days (d1-d15), and asciminib was also administered for 14 days (d1-d15) at 40 or 200 mg BID and 80 mg QD doses.
[3] Asciminib was administered on Day 3 one hour prior to the administration of the probe substrate to maximize the interaction effect.
[4] Values correspond to the AUClast ratio, as the model did not output the AUC_{inf} ratio.

Table S15. Summary of asciminib pharmacokinetics after oral administration of a 40, 80, or 200 mg single dose of asciminib in healthy adults and subjects with impaired hepatic function.

**Trial**[1] [5]	**Geometric Mean C_{max} (ng/mL)**				**Geometric Mean AUC_{inf} (ng·h/mL)**			
**Asciminib dose (mg)**	**40**		**80**	**200**	**40**		**80**	**200**
	Observed[2]	Simulated	Simulated	Simulated	Observed[2]	Simulated	Simulated	Simulated
Healthy volunteers (HV, control)	578	643	1297	3301	4910	5974	13247	37691
Mild hepatic impairment	731	621	1253	3189	5980	6657	14978	43273
**Geometric Mean Ratio, Mild/HV (90% CI)**[3]	**1.26 (1.05, 1.52)**	**0.966**	0.965	0.966	**1.22 (0.964, 1.54)**	**1.11**	1.13 [1.18]	1.15 [1.19]
%PE[4]		*%PE=-23.3*				*%PE=-9.02*		
Moderate hepatic impairment	568	584	1177	2989	5050	7857	18028	53089
**Geometric Mean Ratio, Moderate/HV (90% CI)**[3]	**0.983 (0.819, 1.18)**	**0.908**	0.907	0.905	**1.03 (0.813, 1.30)**	**1.32**	1.36	1.41
%PE[4]		*%PE=-7.63*				*%PE=28.2*		
Severe hepatic impairment	746	499	1003	2542	8160	7650	17875	53614
**Geometric Mean Ratio, Severe/HV (90% CI)**[3]	**1.29 (1.08, 1.55)**	**0.776**	0.773	0.770	**1.66 (1.3, 2.12)**	**1.28**	1.35	1.42
%PE[4]		*%PE=-39.8*				*%PE=-22.9*		

**Trial**[1] [5]	**Geometric Mean C_{max} (ng/mL)**				**Geometric Mean AUC_{inf} (ng·h/mL)**			
**Asciminib dose (mg)**	**40**		**80**	**200**	**40**		**80**	**200**
	Observed[2]	Simulated	Simulated	Simulated	Observed[2]	Simulated	Simulated	Simulated

[1]The number of subjects, age range, and proportion of females in the study by [5] were used. The simulated trials consisted of 10 trials of 10 subjects (n=100), with the age range and proportion of females matching the actual demographics of the respective clinical studies. The population model used for the HV (control), mild, moderate, and severe hepatic impairment simulations was the North European Caucasian (NEC), with matched demographics for the hepatic impairment populations, the Sim-CP-A, the Sim-CP-B, and the Sim-CP-C model, respectively. Especially for the severe hepatic impairment, a modified Simcyp CP-C population with reduced UGT2B7 and UGT1A4 abundances (according to [11]) was used.
[2]Observed values are reported as the adjusted geometric mean values.
[3]The predicted geometric mean AUC and Cmax ratios were within 40% of the actual values.
[4]PE%, calculated prediction error (%) = [(predicted value – observed value)/observed value] x 100.

Table S16. Summary of asciminib pharmacokinetics after oral administration of a 40, 80, or 200 mg single dose of asciminib in healthy adults and subjects with impaired renal function.

**Trial**[1] [5]	**Geometric Mean C_{max} (ng/mL)**				**Geometric Mean AUC_{inf} (ng·h/mL)**			
**Asciminib dose (mg)**	**40**		**80**	**200**	**40**		**80**	**200**
	Observed[2]	Simulated	Simulated	Simulated	Observed[2]	Simulated	Simulated	Simulated
Healthy volunteers (HV, control)	564	682	1377	3510	5550	6550	14616	41777
Mild renal impairment[3]	–	627 [869][6]	1265 [1755]	3222 [4479]	–	6004 [8877]	13442 [19890]	38555 [57187]
**Geometric Mean Ratio, Mild/HV (90% CI)**	–	**0.919 [1.27]**	**0.919 [1.27]**	**0.918 [1.28]**	–	**0.917 [1.36]**	**0.920 [1.36]**	**0.923 [1.37]**
Moderate renal impairment	–	631 [875]	1274 [1766]	3242 [4503]	–	6466 [9585]	14654 [21746]	42636 [63447]
**Geometric Mean Ratio, Moderate/HV (90% CI)**	–	**0.925 [1.28]**	**0.924 [1.28]**	**0.924 [1.28]**	–	**0.987 [1.46]**	**1.00 [1.49]**	**1.02 [1.52]**
Severe renal impairment	607	558 [776]	1125 [1566]	2864 [3991]	8630	6355 [9425]	14854 [22066]	44949 [67009]
**Geometric Mean Ratio, Severe/HV (90% CI)**	**1.08 (0.719, 1.61)**	**0.818 [1.14]**	**0.817 [1.14]**	**0.816 [1.14]**	**1.56 (1.05, 2.30)**	**0.970 [1.44]**	**1.02 [1.51]**	**1.08 [1.60]**
%PE[4]		*%PE=-24.3 [%PE=5.56]*				*%PE=-37.8 [%PE=-7.69]*		

Trial[1] [5]	Geometric Mean C_{max} (ng/mL)				Geometric Mean AUC_{inf} (ng·h/mL)			
**Asciminib dose (mg)**	**40**		**80**	**200**	**40**		**80**	**200**
	Observed[2]	Simulated	Simulated	Simulated	Observed[2]	Simulated	Simulated	Simulated

--, not available.

[1]The number of subjects, age range, and proportion of females in the study by [5] were used. The simulated trials consisted of 10 trials of 10 subjects (n=100), with the age range and proportion of females matching the actual demographics of the respective clinical studies. Simulations in mild and moderate renal impairments were performed using the same demographics as those in the severe renal impairment, as observed data/demographics were not available. The population model used for the HV (control), moderate, and severe renal impairment simulations was the North European Caucasian (NEC), with matched demographics for the renal impairment populations, the RenalGFR_30-60, and the RenalGFR_less_30 models, respectively.

[2]Observed values are reported as the adjusted geometric mean values.

[3]Virtual populations for mild renal impairment (GFR=60-89 mL/min/1.73 m^2) are currently not available in the Simcyp Simulator v19.1. By adjusting the ReanlGFR_30-60 virtual population model, a mild renal impairment population was created and used. The GFR was set at 60-89 mL/min/1.73 m^2 and the serum creatinine at 93-102 μmol/L, and the liver CYP3A4 abundance was equal to the abundance of healthy volunteers (liver CYP3A4 abundance = 137 pmol/mg) according to [12].

[4]The predicted geometric mean AUC and Cmax ratios were within 40% of the actual values.

[5]PE%, calculated prediction error (%) = [(predicted value – observed value)/observed value] x 100.

[6]Values within brackets correspond to the predictions with the ‘top-down’ fraction unbound in plasma for the severe renal impairment ($fu_{p,adj}$=0.018).

Supplementary Figures

Figure S1. Parameter sensitivity analysis of $f_{u,gut}$ and Q_{gut} on the AUC and C_{max} ratios of the midazolam–asciminib DDI.

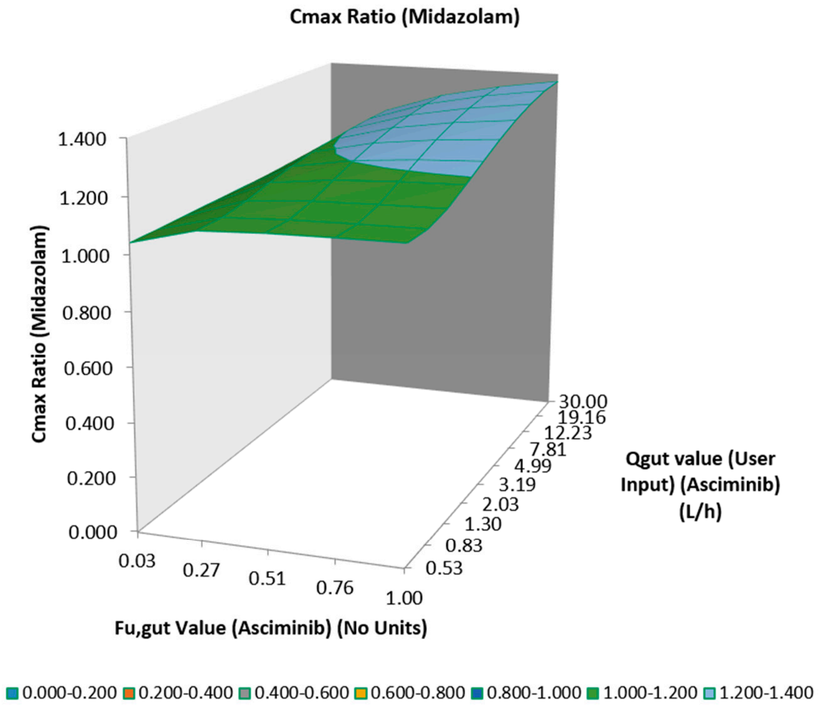

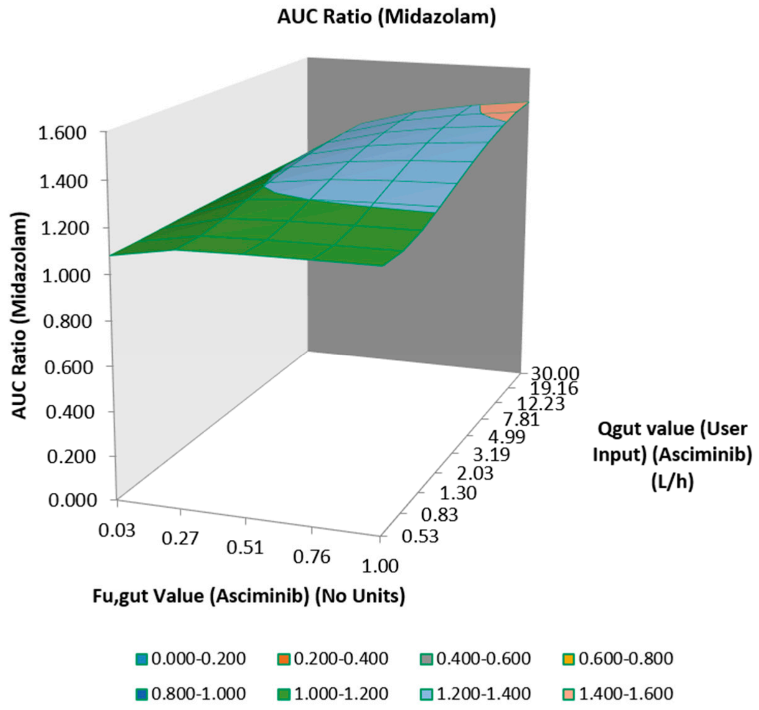

Figure S2. Parameter sensitivity analysis of CYP2C9 *Ki* on the warfarin AUC and C_{max} ratios.

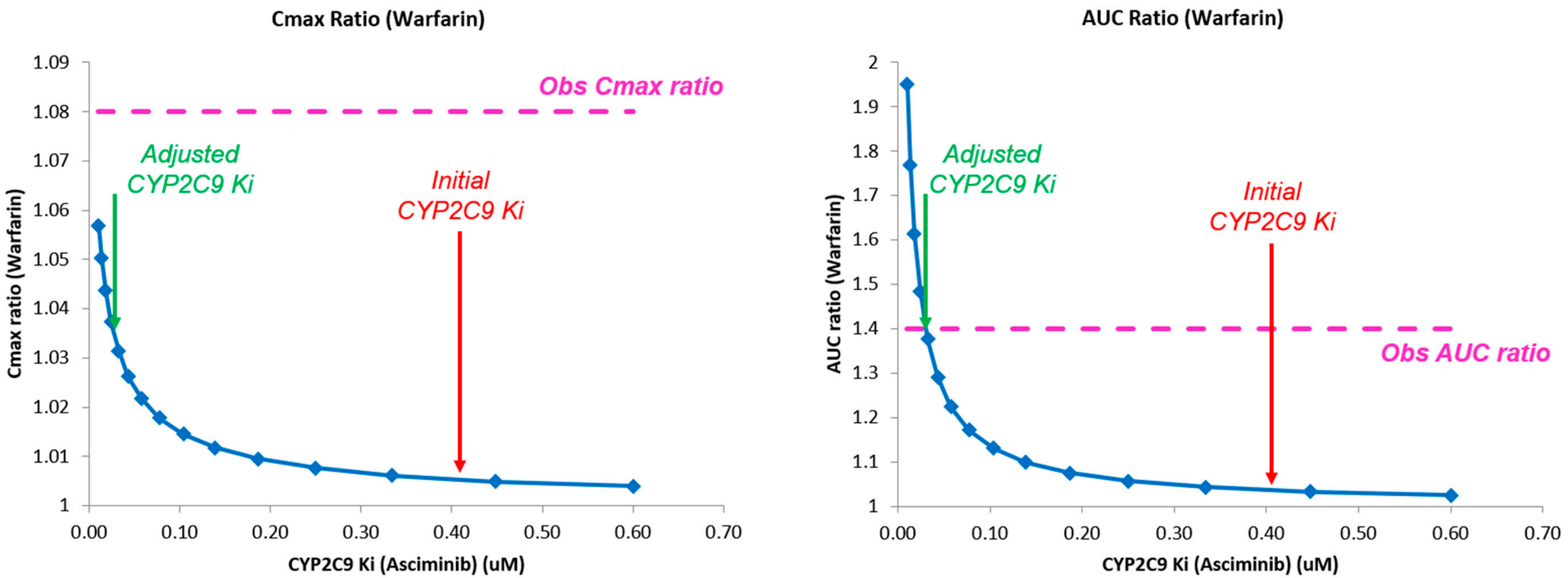

Figure S3. Parameter sensitivity analysis of imatinib's intestinal and hepatic BCRP K_i values on asciminib AUC and C_{max} ratios.

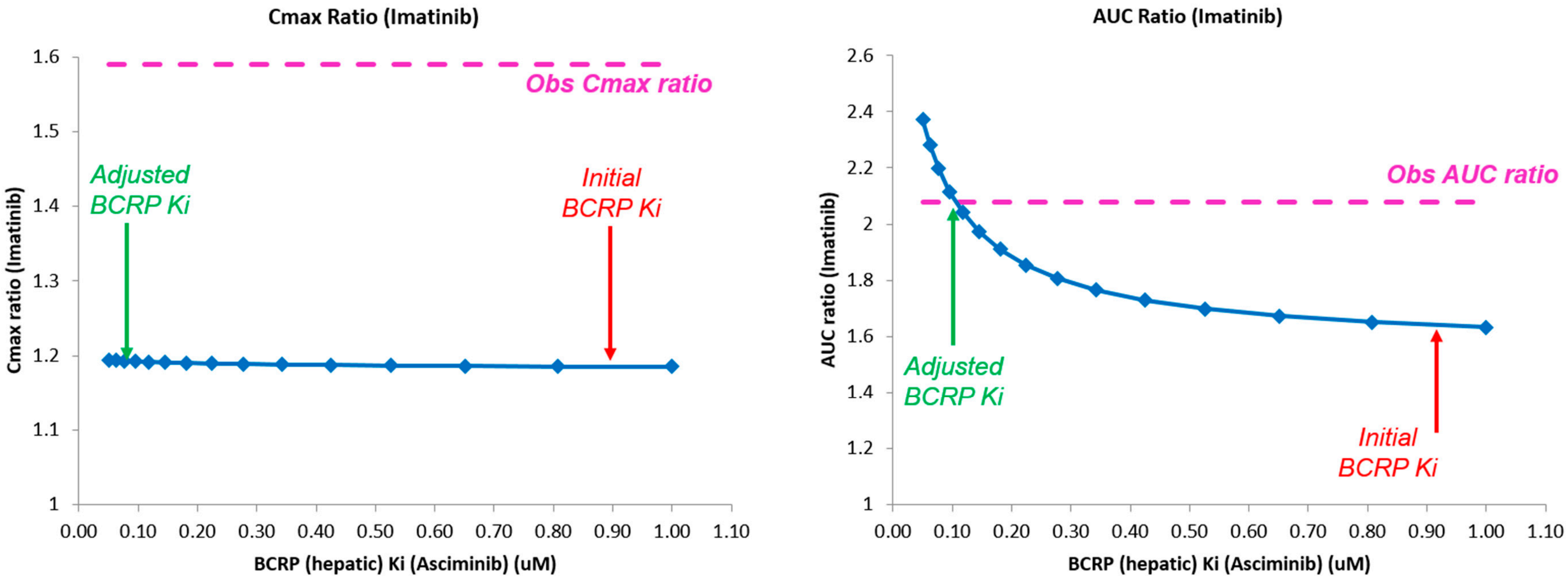

Figure S4. Simulated and observed plasma concentration–time profiles of asciminib (40 mg single dose) in healthy subjects and healthy subjects with mild, moderate, or severe hepatic impairment.

Blue solid lines and triangles represent the arithmetic mean simulated population PK profile and the mean clinically observed PK data with error bars, respectively. The light-blue shaded area covers from the 5th to the 95th percentiles of the simulated PK. PBPK simulations are presented on linear (**(a)**, **(c)**, and **(e)**) as well as on semi-logarithmic scales (**(b)**, **(d)**, and **(f)**).

(a), **(b)**, **(c)**, **(d)**, **(e)**, **(f)**, **(g),** and **(h)**: Population simulation results using the established asciminib PBPK model.

(g) and **(h)**: Population simulation results using a modified CP-C population, accounting for the reduced activities of UGT1A4 and UGT2B7 [11].

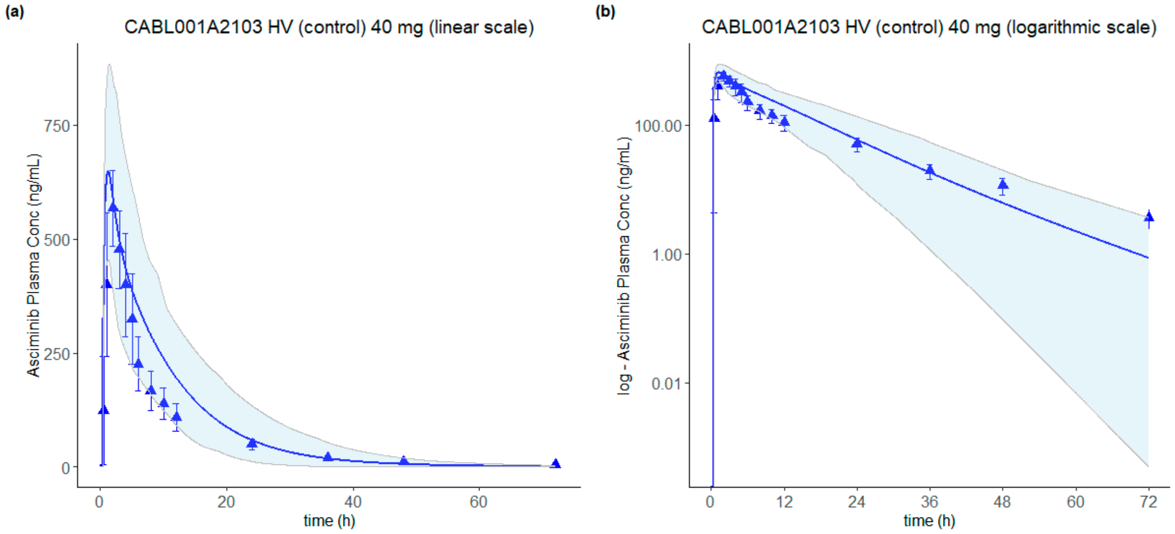

(c)

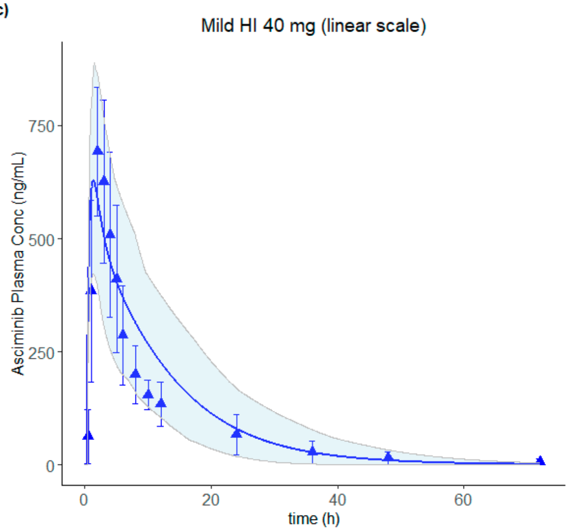

Mild HI 40 mg (linear scale)
Asciminib Plasma Conc (ng/mL)
750
500
250
0
0
20
40
60
time (h)

(d)

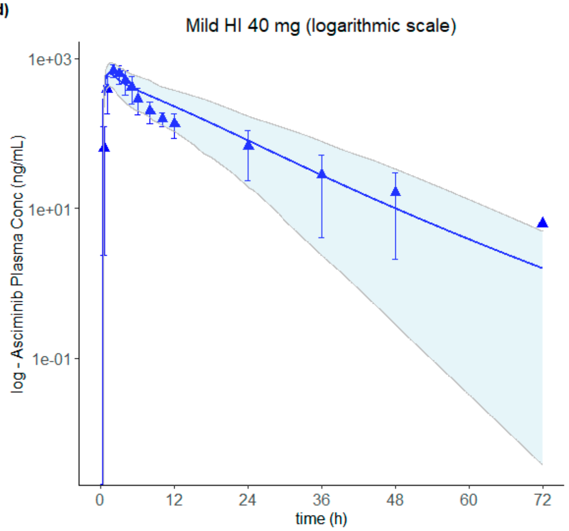

Mild HI 40 mg (logarithmic scale)
log - Asciminib Plasma Conc (ng/mL)
1e+03
1e+01
1e-01
0
12
24
36
48
60
72
time (h)

(e)

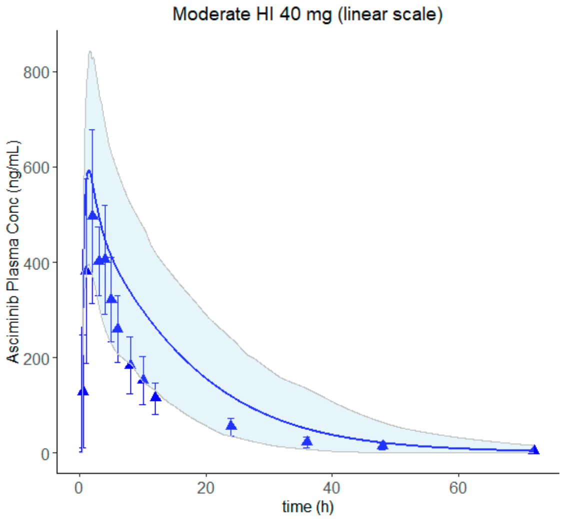
Moderate HI 40 mg (linear scale)
Asciminib Plasma Conc (ng/mL)
800
600
400
200
0
0
20
40
60
time (h)

(f)

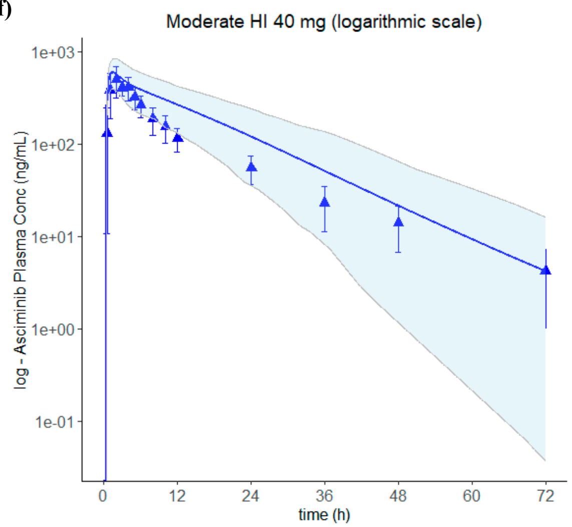
Moderate HI 40 mg (logarithmic scale)
log - Asciminib Plasma Conc (ng/mL)
1e+03
1e+02
1e+01
1e+00
1e-01
0
12
24
36
48
60
72
time (h)

(g)

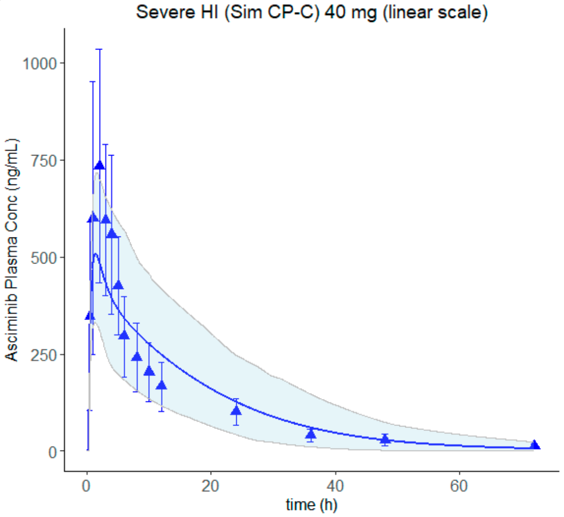
Severe HI (Sim CP-C) 40 mg (linear scale)
Asciminib Plasma Conc (ng/mL)
1000
750
500
250
0
0
20
40
60
time (h)

(h)

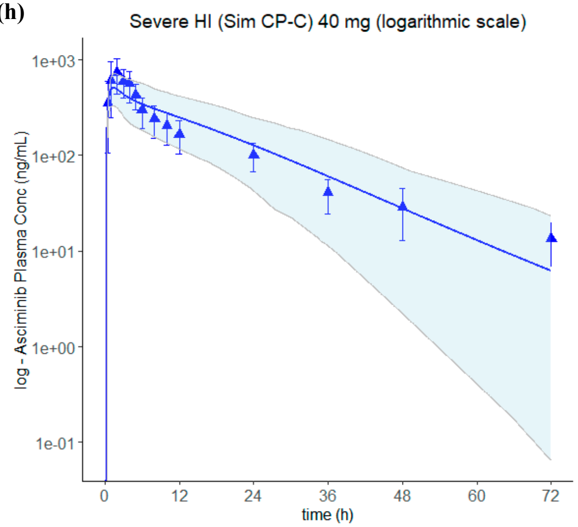
Severe HI (Sim CP-C) 40 mg (logarithmic scale)
log - Asciminib Plasma Conc (ng/mL)
1e+03
1e+02
1e+01
1e+00
1e-01
0
12
24
36
48
60
72
time (h)

Figure S5. Simulated and observed plasma concentration–time profiles of asciminib (40 mg single dose) in healthy subjects and healthy subjects with mild, moderate, or severe renal impairment.

Blue solid lines and triangles represent the arithmetic mean simulated population PK profile and the mean clinically observed PK data with error bars, respectively. The light-blue shaded area covers from the 5th to the 95th percentiles of the simulated PK. PBPK simulations are presented on linear **(a)** and **(c)** as well as on semi-logarithmic scales **(b)** and **(d)**.

(a)

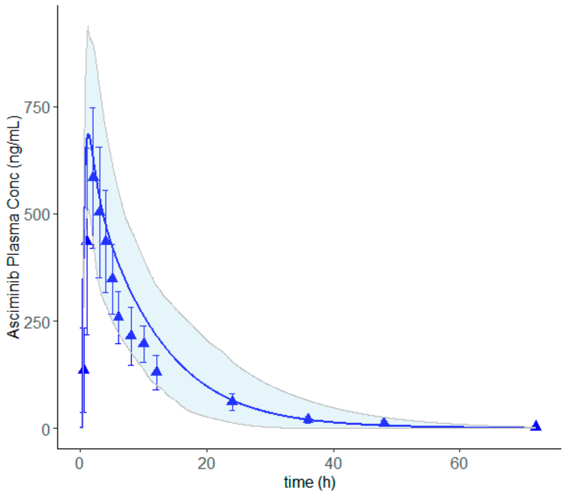

(b)

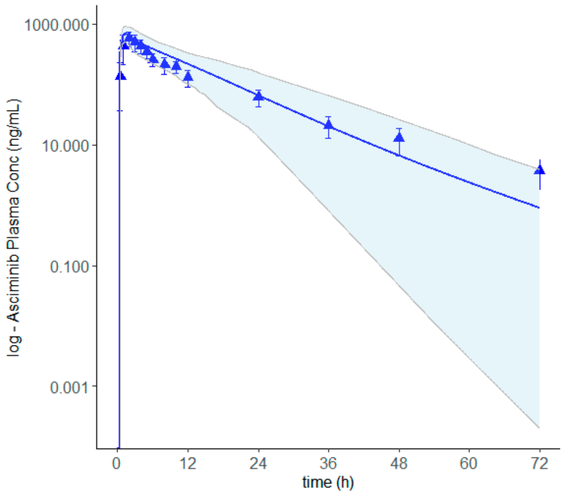

(c)

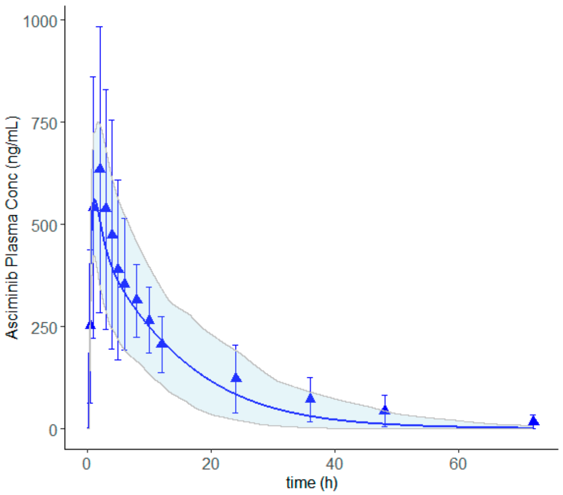

1000
750
500
250
0
Asciminib Plasma Conc (ng/mL)
0
20
40
60
time (h)

(d)

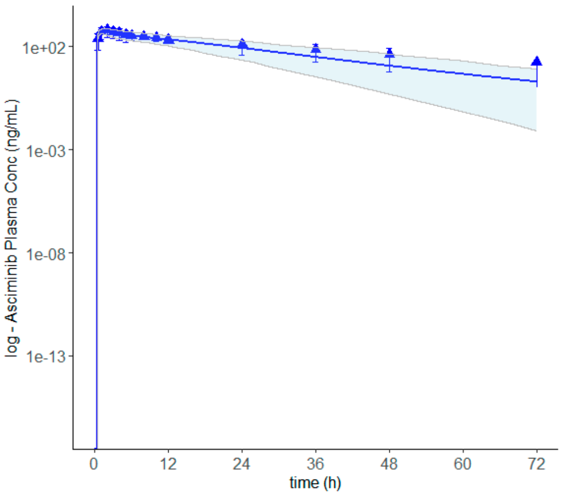

1e+02
1e-03
1e-08
1e-13
log - Asciminib Plasma Conc (ng/mL)
0
12
24
36
48
60
72
time (h)

Figure S6. Parameter sensitivity analysis of the fraction unbound in plasma on C_{max} and AUC for subjects with severe renal impairment.

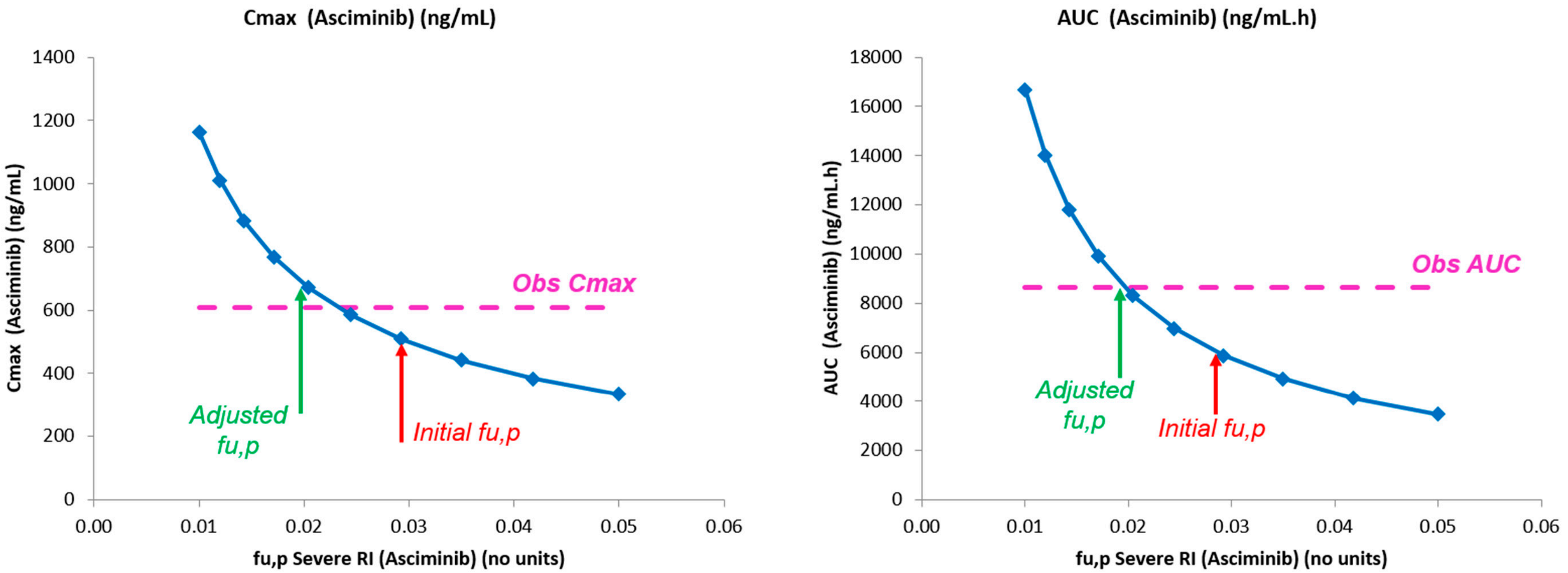

References

1. Hoch, M.; Huth, F.; Manley, P.W.; Loisios-Konstantinidis, I.; Combes, F.P.; Li, Y.F.; Fu, Y.; Sy, S.K.B.; Obourn, V.; Chakraborty, A.; et al. Clinical Pharmacology of Asciminib: A Review. *Clin Pharmacokinet* **2024**, *63*, doi:10.1007/S40262-024-01428-6.
2. Davies, B.; Morris, T. Physiological Parameters in Laboratory Animals and Humans. *Pharm Res* **1993**, *10*, 1093–1095, doi:10.1023/A:1018943613122/METRICS.
3. Yang, J.; Jamei, M.; Yeo, K.; Tucker, G.; Rostami-Hodjegan, A. Prediction of Intestinal First-Pass Drug Metabolism. *Curr Drug Metab* **2007**, *8*, 676–684, doi:10.2174/138920007782109733.
4. Hoch, M.; Huth, F.; Sato, M.; Sengupta, T.; Quinlan, M.; Dodd, S.; Kapoor, S.; Hourcade-Potelleret, F. Pharmacokinetics of Asciminib in the Presence of CYP3A or P-Gp Inhibitors, CYP3A Inducers, and Acid-Reducing Agents. *Clin Transl Sci* **2022**, *15*, 1698–1712, doi:10.1111/cts.13285.
5. Hoch, M.; Sato, M.; Zack, J.; Quinlan, M.; Sengupta, T.; Allepuz, A.; Aimone, P.; Hourcade-Potelleret, F. Pharmacokinetics of Asciminib in Individuals With Hepatic or Renal Impairment. *J Clin Pharmacol* **2021**, *61*, 1454–1465, doi:10.1002/jcph.1926.
6. Hughes, T.P.; Mauro, M.J.; Cortes, J.E.; Minami, H.; Rea, D.; DeAngelo, D.J.; Breccia, M.; Goh, Y.-T.; Talpaz, M.; Hochhaus, A.; et al. Asciminib in Chronic Myeloid Leukemia after ABL Kinase Inhibitor Failure. *New England Journal of Medicine* **2019**, *381*, 2315–2326, doi:10.1056/nejmoa1902328.
7. Réa, D.; Mauro, M.J.; Boquimpani, C.; Minami, Y.; Lomaia, E.; Voloshin, S.; Turkina, A.; Kim, D.W.; Apperley, J.F.; Abdo, A.; et al. A Phase 3, Open-Label, Randomized Study of Asciminib, a STAMP Inhibitor, vs Bosutinib in CML after 2 or More Prior TKIs. *Blood* **2021**, *138*, 2031–2041, doi:10.1182/BLOOD.2020009984.
8. Hoch, M.; Zack, J.; Quinlan, M.; Huth, F.; Forte, S.; Dodd, S.; Aimone, P.; Hourcade-Potelleret, F. Pharmacokinetics of Asciminib When Taken With Imatinib or With Food. *Clin Pharmacol Drug Dev* **2022**, *11*, 207–219, doi:10.1002/cpdd.1019.
9. Chen, Y.; Cabalu, T.D.; Callegari, E.; Einolf, H.; Liu, L.; Parrott, N.; Peters, S.A.; Schuck, E.; Sharma, P.; Tracey, H.; et al. Recommendations for the Design of Clinical Drug–Drug Interaction Studies With Itraconazole Using a Mechanistic Physiologically-Based Pharmacokinetic Model. *CPT Pharmacometrics Syst Pharmacol* **2019**, *8*, 685–695, doi:10.1002/PSP4.12449.
10. Filppula, A.M.; Neuvonen, M.; Laitila, J.; Neuvonen, P.J.; Backman, J.T. Autoinhibition of CYP3A4 Leads to Important Role of CYP2C8 in Imatinib Metabolism: Variability in CYP2C8 Activity May Alter Plasma Concentrations and Response. *Drug Metabolism and Disposition* **2013**, *41*, 50–59, doi:10.1124/DMD.112.048017/-/DC1.
11. Prasad, B.; Bhatt, D.K.; Johnson, K.; Chapa, R.; Chu, X.; Salphati, L.; Xiao, G.; Lee, C.; Hop, C.E.C.A.; Mathias, A.; et al. Abundance of Phase 1 and 2 Drug-Metabolizing Enzymes in Alcoholic and Hepatitis C Cirrhotic Livers: A Quantitative Targeted Proteomics Study. *Drug Metabolism and Disposition* **2018**, *46*, 943–952, doi:10.1124/DMD.118.080523/-/DC1.

12. Heimbach, T.; Chen, Y.; Chen, J.; Dixit, V.; Parrott, N.; Peters, S.A.; Poggesi, I.; Sharma, P.; Snoeys, J.; Shebley, M.; et al. Physiologically-Based Pharmacokinetic Modeling in Renal and Hepatic Impairment Populations: A Pharmaceutical Industry Perspective. *Clin Pharmacol Ther* **2021**, *110*, 297–310, doi:10.1002/CPT.2125.
13. Hoch, M.; Sengupta, T.; Hourcade-Potelleret, F. Pharmacokinetic Drug Interactions of Asciminib with the Sensitive Cytochrome P450 Probe Substrates Midazolam, Warfarin, and Repaglinide in Healthy Participants. *Clin Transl Sci* **2022**, *15*, 1406–1416, doi:10.1111/cts.13252.
